# Timing and dose of acupuncture as adjuncts to assisted reproduction: a meta-analysis and model-based network meta-analysis

**DOI:** 10.3389/fendo.2026.1880225

**Published:** 2026-07-09

**Authors:** Jiale Wei, Yue Wang, Kailin Zhang, Zheng Shen, Jialin Li, Hong Guan, Jian Wang

**Affiliations:** 1 Shandong University of Traditional Chinese Medicine, Jinan, China; 2 Department of Acupuncture, Shandong University of Traditional Chinese Medicine Affiliated Hospital, Jinan, China

**Keywords:** acupuncture, assisted reproductive technology, dose-response relationship, *in vitro* fertilization, network meta-analysis

## Abstract

**Objective:**

Acupuncture is widely used as an adjunct to assisted reproductive technology (ART), but its effects and the influence of treatment timing, modality, and dose remain uncertain. This study evaluated acupuncture-related interventions in ART and explored potential timing- and dose-response patterns.

**Methods:**

PubMed, Embase, Web of Science, Scopus, the Cochrane Library, and Ovid MEDLINE were searched from inception to January 19, 2026, with additional screening of eligible study and review reference lists. Randomized controlled trials of women undergoing *in vitro* fertilization, intracytoplasmic sperm injection, or embryo transfer were included when manual acupuncture, electroacupuncture, or transcutaneous electrical acupoint stimulation was used as an adjunctive ART intervention. Pairwise random-effects meta-analysis estimated overall effects; network meta-analysis compared intervention timing, modality, and needle-retention time; and model-based network meta-analysis examined dose-response associations by treatment sessions. Risk of bias was assessed using RoB 2, and certainty of evidence was rated with GRADE.

**Results:**

Forty-two trials including 9,390 women were included. Acupuncture was associated with higher clinical pregnancy rate [risk ratio (RR) = 1.25; 95% confidence interval (CI), 1.14-1.37] and implantation rate (RR = 1.16; 95% CI, 1.02-1.31), whereas the effect on live birth was uncertain (RR = 1.14; 95% CI, 0.98-1.32). Certainty of evidence for all overall major outcomes was very low. Timing analyses suggested higher estimates for acupuncture during controlled ovarian hyperstimulation, or during both controlled ovarian hyperstimulation and embryo transfer, than for acupuncture limited to embryo transfer; however, timing was closely correlated with cumulative treatment sessions, and these findings should be interpreted as exploratory. Dose-response models suggested possible nonlinear associations between treatment sessions and reproductive outcomes, but these model-based estimates were limited by sparse data, clinical heterogeneity, and model uncertainty and should not be used as treatment-dose recommendations.

**Conclusions:**

Acupuncture as an adjunct to ART may improve clinical pregnancy and implantation rates, although the certainty of evidence is very low. The observed timing- and dose-response patterns provide exploratory, hypothesis-generating evidence regarding potentially beneficial treatment strategies but are insufficient to establish the superiority of a specific timing or number of sessions. Further well-designed trials are needed to confirm these findings.

**Systematic review registration:**

https://www.crd.york.ac.uk, identifier CRD420261291016.

## 1 Introduction

With the continued development of assisted reproductive technology (ART), IVF, ICSI, embryo cryopreservation, and frozen-thawed embryo transfer have become important components of infertility treatment (1, 2). Recent registry data from the United States and Europe show continuing increases in ART treatment cycles and ART-conceived live births, together with wider use of single embryo transfer and frozen-thawed embryo transfer (3, 4). Nevertheless, ART does not fully overcome the risk of reproductive failure; treatment outcomes remain influenced by maternal age, oocyte and embryo quality, endometrial status, embryo-endometrial synchrony, previous treatment response, and variation across treatment centers (5, 6). ART treatment is also complex and costly and is associated with substantial psychological burden (7–9). Acupuncture has therefore attracted sustained interest as a potentially safe, minimally invasive adjunct that can be integrated into treatment cycles and may improve both peri-treatment well-being and reproductive outcomes.

Acupuncture, a commonly used nonpharmacologic adjunctive intervention, has been evaluated in IVF/ICSI and embryo transfer settings for improving pregnancy outcomes, relieving discomfort related to oocyte retrieval or embryo transfer, and reducing anxiety and pain during treatment (10, 11). Previous systematic reviews and meta-analyses have suggested that acupuncture may improve intermediate outcomes, such as clinical pregnancy, biochemical pregnancy, or implantation rates, but its effects on live birth, ongoing pregnancy, and miscarriage rates remain inconsistent. Reviews have also differed in how they handled sham acupuncture, no-treatment controls, and routine-care controls (12–15). Some studies further suggest that acupuncture effects may depend not only on whether acupuncture is used, but also on the ART stage at which treatment is delivered, acupuncture modality, course length, treatment frequency, and total number of sessions (16, 17). From a clinical implementation perspective, acupuncture during controlled ovarian hyperstimulation, around embryo transfer, during the luteal phase, or across multiple stages may correspond to different physiological contexts and therapeutic aims. Manual acupuncture, electroacupuncture, and transcutaneous electrical acupoint stimulation also differ in stimulation intensity, delivery method, and acceptability. Combining these heterogeneous regimens into a single acupuncture category may therefore dilute true effects.

Several methodologic limitations remain in the existing evidence synthesis. First, most systematic reviews have focused on pairwise comparisons of acupuncture versus control interventions, with limited evaluation of different intervention timings as network nodes that can be compared with one another. This limits the direct usefulness of the findings for selecting clinical regimens. Second, previous studies of acupuncture dose have often categorized the number of sessions as high, medium, or low dose. Although convenient for description, this approach may sacrifice continuous dose information and obscure nonlinear dose-response associations. Too few sessions may be insufficient to produce an effect, whereas excessive sessions may increase burden and reduce completion rates (18, 19). Conventional linear or categorical dose models are poorly suited to identify potential nonlinear dose ranges or to build dose-response curves across acupuncture modalities and ART timing nodes. Therefore, based on a systematic review and pairwise meta-analysis, we further used network meta-analysis to compare the relative effects of different acupuncture timing nodes and modalities. We then applied model-based network meta-analysis (MBNMA), treating the number of acupuncture sessions as a continuous dose variable (20), to explore dose-response relationships and generate hypotheses about session ranges associated with larger model-based estimates for acupuncture as an adjunct to ART.

## 2 Materials and methods

### 2.1 Protocol and registration

This study was designed, analyzed, and reported in accordance with PRISMA 2020 and the PRISMA-NMA extension statement and was prospectively registered in PROSPERO (CRD420261291016). All data were derived from published studies; Therefore, additional ethics approval was not required.

### 2.2 Search strategy

We systematically searched PubMed, Embase, Web of Science, Scopus, the Cochrane Library, and Ovid MEDLINE from inception to January 19, 2026. Search terms combined controlled vocabulary and free-text terms related to assisted reproductive technology, IVF/ICSI, embryo transfer, acupuncture, electroacupuncture, and transcutaneous electrical acupoint stimulation. No language restrictions were applied. References of included studies and relevant reviews were also screened. The full search strategy is provided in **Supplementary File 1**.

### 2.3 Eligibility criteria

Prospective randomized controlled trials were eligible if they enrolled women with infertility undergoing IVF, ICSI, or embryo transfer. Eligible interventions included manual acupuncture, electroacupuncture, or transcutaneous electrical stimulation used as adjunctive ART interventions. Comparators included sham acupuncture, no acupuncture, wait-list control, or routine ART care. Studies were required to report at least 1 reproductive outcome, such as live birth rate, clinical pregnancy rate, ongoing pregnancy rate, biochemical pregnancy rate, implantation rate, or miscarriage rate. Nonrandomized studies, reviews, case reports, animal studies, duplicate publications, studies combining other adjunctive therapies, and studies without extractable data were excluded. These comparator conditions were combined as non-verum or no-verum-acupuncture controls for the primary synthesis, but they were not considered physiologically equivalent; comparator class was therefore examined in subgroup analyses and retained as a potential source of indirectness and intransitivity.

### 2.4 Study selection and data extraction

After deduplication in EndNote X9, records were imported into ASReview (version 2.2) to assist title and abstract screening. ASReview was used only to improve screening efficiency (21); final inclusion decisions were made independently by 2 reviewers. The stopping rule, initial training records, manual verification scope, and procedures to reduce missed studies are described in **Supplementary File 2**. Full texts judged potentially eligible were then imported into Zotero. Two authors independently screened full texts and extracted data, with disagreements resolved by discussion or adjudication by a third author. Extracted items included study characteristics, patient characteristics, cycle type, acupuncture modality, intervention timing, number of treatment sessions, needle-retention time, comparator type, blinding, and reproductive outcomes. To address heterogeneity more transparently, we additionally summarized patient, ART-cycle, and intervention-level effect modifiers, including diagnostic restriction, transfer type, modality, timing, dose, frequency, acupoints, stimulation parameters, and comparator type (**Supplementary File 3**).

### 2.5 Risk of bias and certainty of evidence

Two authors assessed risk of bias in included studies using RoB 2 (22), and certainty of evidence was evaluated with GRADE (23). RoB 2 judgments focused on the randomization process, deviations from intended interventions, missing outcome data, outcome measurement, and selective reporting. For no-treatment or wait-list controls, special attention was given to limitations in blinding, adherence, crossover, cointerventions, and information on intention-to-treat analyses. GRADE downgrading considered risk of bias, inconsistency, indirectness, imprecision, and publication bias. For outcomes with at least 10 studies, funnel plots and Egger tests were used to assess small-study effects, with trim-and-fill analysis performed as an exploratory correction when needed. Detailed operational criteria for applying RoB 2 are provided in **Supplementary File 4**.

### 2.6 Statistical analysis

#### 2.6.1 Pairwise meta-analysis

Pairwise meta-analyses were performed using the R package meta (version 8.2-1). Given clinical heterogeneity across populations, cycle types, acupuncture protocols, and comparator types, random-effects models were used for the main analyses; fixed-effect models were used for sensitivity or robustness comparisons. Heterogeneity was assessed with I² and the Cochran’s Q test. Leave-one-out sensitivity analyses were conducted using metainf.

Subgroup analyses and meta-regression were conducted for categorical variables, including transfer type, intervention timing, acupuncture modality, and comparator type, and for continuous variables, including mean age, BMI, proportion with primary infertility, and infertility duration. We also used a flexible approach integrating meta-classification and regression trees (meta-CART). This method has been shown to improve identification of influential covariates (24), particularly when multiple moderators are present. Missing covariate data were handled with multiple imputation by chained equations (20 imputations) (25). Moderator analyses were interpreted as exploratory; variables with sparse or inconsistent reporting were summarized descriptively rather than forced into multivariable models. Given the correlation between intervention timing and cumulative treatment exposure, timing-related subgroup results were interpreted together with session number rather than as isolated timing effects.

#### 2.6.2 Network meta-analysis

##### 2.6.2.1 Conventional network meta-analysis

Network meta-analyses were conducted separately for acupuncture modality, needle-retention time, and intervention timing. Definitions of the intervention timing nodes are provided in **Supplementary File 5**. Analyses were performed using the R package netmeta (version 3.3-1). In addition to reporting network connectivity, we evaluated the distribution of major effect modifiers across nodes and used node-splitting to explore agreement between direct and indirect evidence. A nonsignificant node-splitting P value was not interpreted as proof of consistency. Network nodes were ranked using the surface under the cumulative ranking curve (SUCRA), and relative effects across nodes were presented in league tables. To strengthen the assessment of transitivity, we examined whether prespecified clinical and methodological effect modifiers were similarly distributed across network nodes. These modifiers included infertility diagnosis or diagnostic restriction, embryo-transfer type, acupuncture modality, intervention timing, treatment intensity, and comparator class. We further explored potential timing-dose confounding using study-level random-effects meta-regression based on log risk ratios for clinical pregnancy and live birth. Intervention timing, number of acupuncture sessions, and comparator type were entered sequentially as covariates to assess whether timing-related estimates were attenuated after accounting for cumulative treatment exposure.

##### 2.6.2.2 Dose-response model-based network meta-analysis

We used random-effects Bayesian MBNMA (26) to explore dose-response relationships between the number of acupuncture sessions and reproductive outcomes. The number of acupuncture sessions was defined as the dose variable, and binary outcomes were entered as event counts and total sample sizes for each study arm. Analyses were conducted at the overall acupuncture level, by acupuncture modality, and by specific intervention timing node to explore outcome trends across modalities and timing strategies. We assessed key assumptions of network meta-analysis, including connectivity, consistency, and transitivity, using model-fit comparisons between consistency and UME models, node-splitting diagnostics, and effect-modifier distributions; these diagnostics were interpreted jointly with DIC, residual deviance, between-study SD, and network sparsity rather than as proof that assumptions were fully satisfied (**Supplementary File 6**). Because convergence of binomial models with a log link was unstable, dose-response analyses primarily used a logit link and reported ORs; these effect estimates should not be directly compared with RRs from pairwise meta-analysis or conventional network meta-analysis. In the dose-response model, control type, including sham acupuncture, sham TEAS, no-treatment, wait-list, and routine-care controls, was not included as a covariate, which may have affected the precision of effect estimates. Therefore, dose-response findings should be interpreted cautiously.

To identify nonlinear dose-response relationships, we fitted and compared multiple dose functions, including Emax models, restricted cubic spline models, quadratic polynomial models, and linear polynomial models. Model selection was based mainly on the deviance information criterion (DIC), residual deviance, between-study heterogeneity SD, and model convergence. For analyses by acupuncture modality, different dose-response functions were allowed across modalities when supported by the data to improve fit to intervention heterogeneity. When a nonlinear dose-response model was fitted, deviation from linearity was assessed with the Wald test. Models were considered to have acceptable convergence when the potential scale reduction factor Rhat was ≤ 1.05. All analyses were performed using the R package MBNMAdose (version 0.5.0).

#### 2.6.3 Sensitivity analyses

Sensitivity analyses were performed to examine the robustness of the main findings. First, leave-one-out sensitivity analyses were conducted for each main pairwise meta-analysis using the metainf function in the R package meta, in which one study was sequentially omitted at a time to assess whether the pooled effect estimate was driven by any single trial. Second, to examine the potential influence of selective reporting, we performed risk-of-bias-based sensitivity analyses by excluding studies judged as having “some concerns” in RoB 2 domain 5, namely selection of the reported result. The main pairwise meta-analyses were repeated for outcomes with sufficient available studies after exclusion of these studies. For network meta-analysis and model-based network meta-analysis, similar sensitivity analyses were conducted when the exclusion of these studies did not compromise network connectivity or dose coverage. When such analyses were not feasible because of sparse data, unstable estimates, or disconnected networks, this was reported descriptively and considered when interpreting the certainty and robustness of the findings.

## 3 Results

### 3.1 Study selection and characteristics of included trials

**Figure 1:**
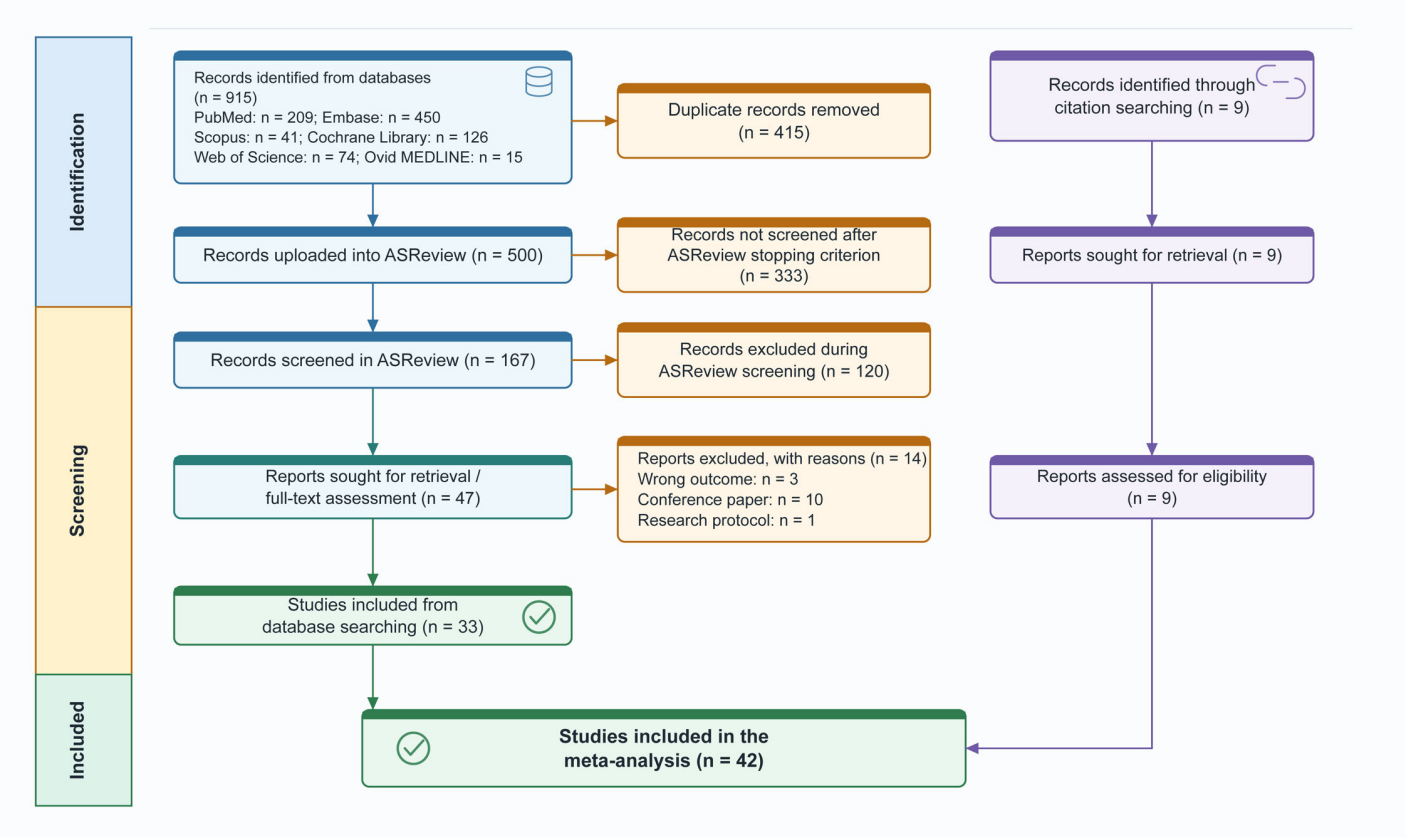
Flow diagram showing the study selection process.

Database searches identified 915 records. After removal of 415 duplicates, 500 unique records were imported into ASReview. The ASReview stopping criterion was reached after 167 records had been screened, of which 120 were excluded during title and abstract screening. Forty-seven full-text reports were subsequently assessed for eligibility, and 14 were excluded because of irrelevant outcomes, conference abstract status, or publication as study protocols only. Thirty-three studies were included from database searches. Citation searching identified 9 additional eligible studies, resulting in 42 studies included in the meta-analysis. The selection process is shown in **Figure 1**.

Forty-two randomized trials (27–68) involving 9,390 women were included. Most trials were conducted in China (25/42). Twenty-six studies evaluated fresh embryo transfer, 3 evaluated frozen-thawed embryo transfer, and 13 included both cycle types. Interventions included manual acupuncture, electroacupuncture, and transcutaneous electrical acupoint stimulation. Comparator groups included routine care/no treatment, sham acupuncture, or sham TEAS. Clinical pregnancy rate and live birth rate were the most frequently assessed outcomes. Characteristics of included studies are provided in **Supplementary File 7**.

### 3.2 Quality assessment

**Figure 2:**
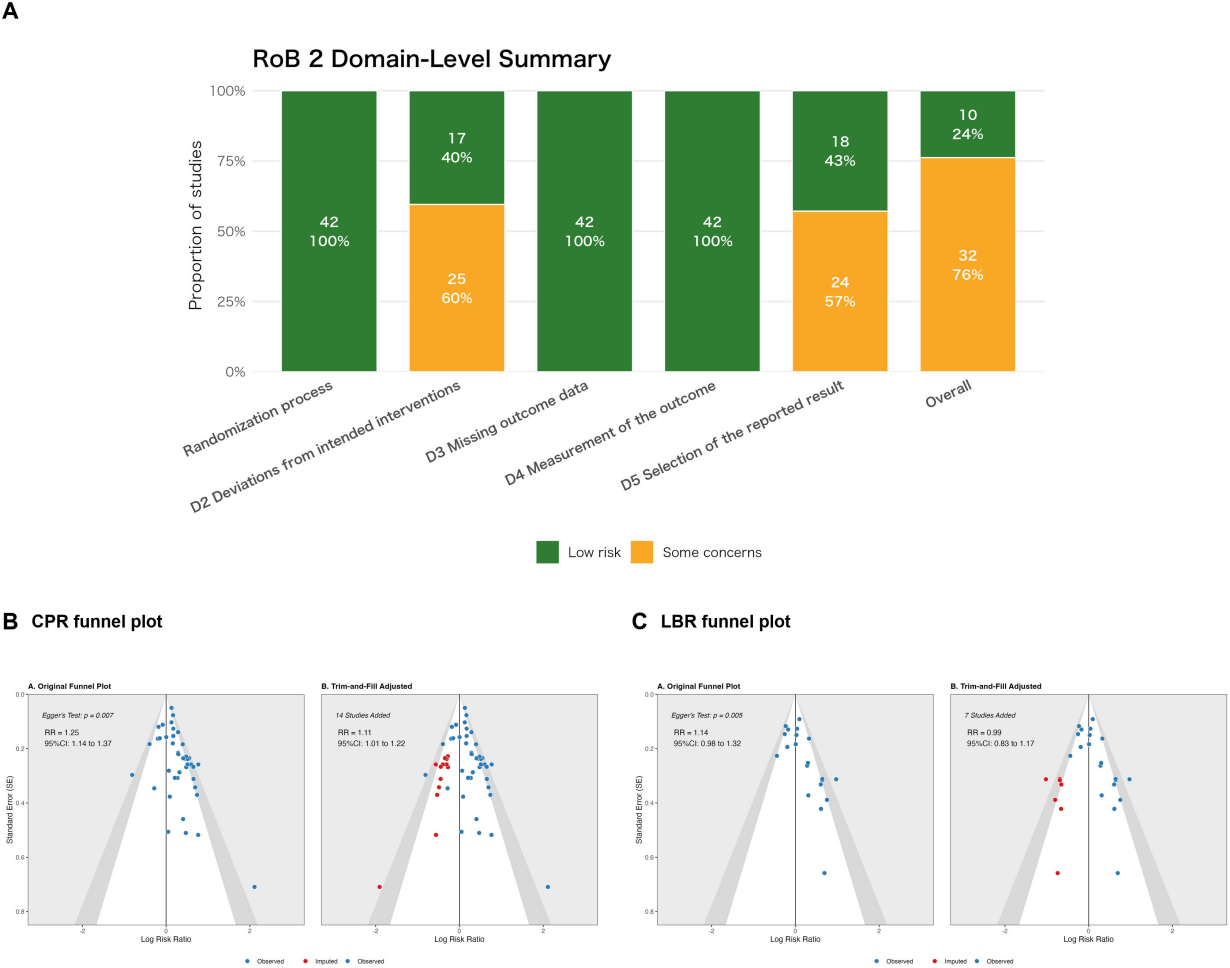
Risk of bias and publication bias assessments for core outcomes. **(A)** RoB 2 domain-level summary across included studies. **(B)** Funnel plot for clinical pregnancy rate (CPR). **(C)** Funnel plot for live birth rate (LBR).

Risk-of-bias assessments are provided in **Supplementary File 8** and **Figure 2A**. RoB 2 assessment indicated that 10 studies were at overall low risk of bias and 32 had some concerns; no study was judged to be at overall high risk. Concerns mainly arose from deviations from intended interventions (domain 2) and selective reporting (domain 5). Domain 2 concerns were commonly related to no-treatment or wait-list controls, limited blinding, and insufficient information on adherence, crossover, and cointerventions. Domain 5 concerns were mainly related to lack of clear prospective registration or prespecified protocols.

### 3.3 Pairwise meta-analysis

**Figure 3:**
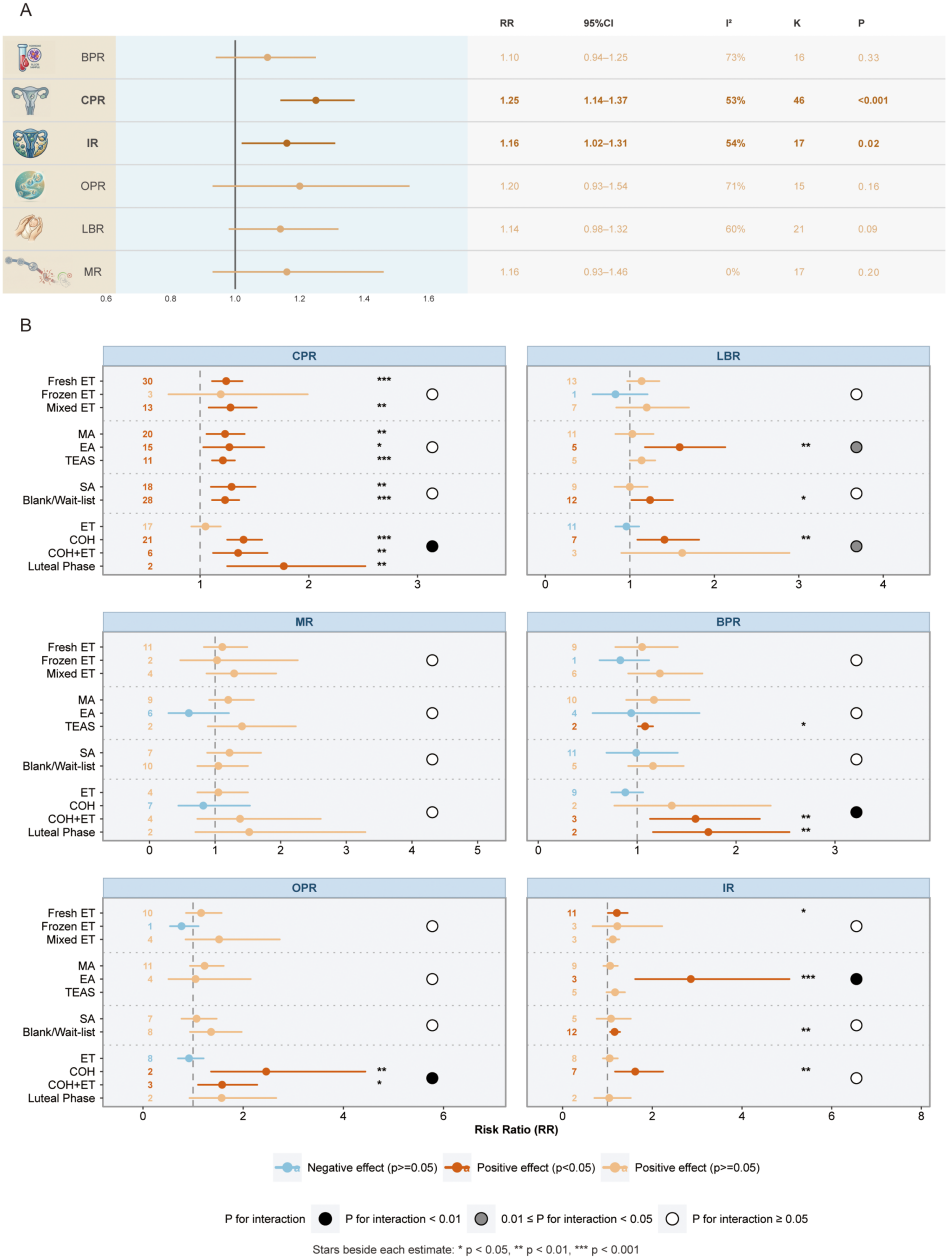
Pairwise meta-analysis and subgroup analyses. **(A)** Forest plot of the overall pairwise meta-analysis for BPR, CPR, IR, OPR, LBR, and MR. **(B)** Forest plot of subgroup analyses stratified by embryo transfer type, acupuncture modality, comparator type, and intervention timing. *P < 0.05; **P < 0.01. RR, risk ratio; CI, confidence interval. *** indicates p < 0.001.

As shown in **Figures 2B, C** and **Supplementary Files 9**, **10**, funnel plots and Egger tests suggested possible publication bias or small-study effects for CPR and LBR. After trim-and-fill adjustment, the CPR effect was attenuated but remained statistically significant, whereas the LBR effect no longer suggested benefit. These findings support cautious interpretation of the results. Acupuncture was associated with higher clinical pregnancy rate (RR = 1.25, 95% CI: 1.14-1.37) and implantation rate (RR = 1.16, 95% CI: 1.02-1.31), as shown in **Figure 3A** and **Supplementary Files 9**, **10**. However, because many studies were judged as having some concerns, heterogeneity was high, and publication bias was possible, the certainty of evidence according to GRADE was rated as very low. Current evidence did not support a statistically significant improvement in live birth rate (RR = 1.14, 95% CI: 0.98-1.32), ongoing pregnancy rate (RR = 1.20, 95% CI: 0.93-1.54), or biochemical pregnancy rate (RR = 1.10, 95% CI: 0.94-1.25). There was also insufficient evidence that acupuncture increased miscarriage rate (RR = 1.16, 95% CI: 0.93-1.46). A concise summary of subgroup analyses and univariable meta-regression is provided in **Supplementary File 11**. Intervention timing emerged as the most consistent potential effect modifier, whereas transfer type and comparator type did not show clear or consistent evidence of effect modification. After adjustment for the number of acupuncture sessions, timing-related estimates for COH and COH + ET compared with ET-only acupuncture were attenuated for both clinical pregnancy and live birth (**Supplementary Material 12**). This attenuation suggests that timing-related differences may partly reflect greater cumulative treatment exposure rather than an independent timing effect.

Intervention timing may be an important modifier of the effects of acupuncture as an adjunct to ART, as shown in **Figure 3B** and **Supplementary File 10**. Acupuncture during COH was associated with higher clinical pregnancy rate (RR = 1.40, 95% CI: 1.25-1.57), live birth rate (RR = 1.41, 95% CI: 1.09-1.82), ongoing pregnancy rate (RR = 2.46, 95% CI: 1.36-4.44), and implantation rate (RR = 1.62, 95% CI: 1.17-2.24). Acupuncture during COH + ET was associated with higher clinical pregnancy rate (RR = 1.35, 95% CI: 1.12-1.62), ongoing pregnancy rate (RR = 1.58, 95% CI: 1.10-2.28), and biochemical pregnancy rate (RR = 1.59, 95% CI: 1.13-2.24). Acupuncture during the luteal phase was associated with higher clinical pregnancy rate (RR = 1.77, 95% CI: 1.25-2.52) and biochemical pregnancy rate (RR = 1.72, 95% CI: 1.16-2.54). By contrast, acupuncture limited to ET did not show a clear association with benefit. Acupuncture modality may influence live birth and implantation rates; electroacupuncture was associated with higher live birth rate (RR = 1.56, 95% CI: 1.18-2.13) and implantation rate (RR = 2.86, 95% CI: 1.62-5.06). GRADE assessment indicated that the certainty of subgroup evidence remained limited overall, with most evidence rated as low or very low. Meta-CART analysis of continuous variables (**Supplementary File 13**) suggested that mean age and infertility duration may influence live birth rate and that BMI may influence miscarriage rate. No clear moderating effect of continuous variables was identified for clinical pregnancy rate, and no meaningful continuous-variable Meta-CART results were found for ongoing pregnancy, implantation, or biochemical pregnancy rates. Sensitivity analysis restricted to studies at low risk in RoB 2 Domain 5 (**Supplementary File 14**) showed generally consistent findings with the primary analysis. CPR (RR = 1.39, 95% CI: 1.23–1.57) and IR (RR = 1.18, 95% CI: 1.05–1.32) remained statistically significant. Notably, LBR became statistically significant after exclusion of studies with some concerns (RR = 1.29, 95% CI: 1.03–1.61), whereas BPR, MR, and OPR remained non-significant.

### 3.4 Network meta-analysis

**Figure 4:**
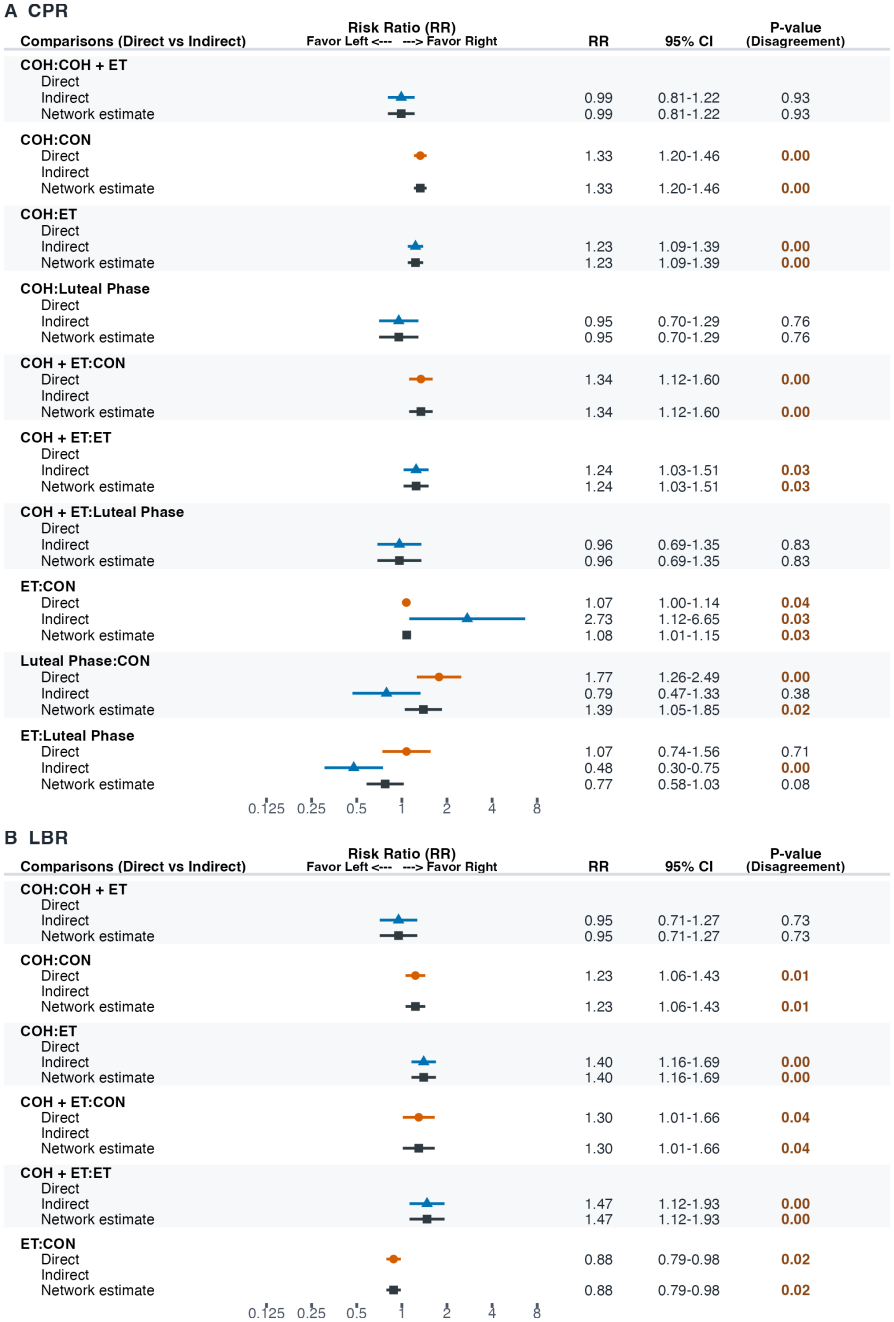
Key consistency diagnostics for intervention timing nodes in network meta-analysis. **(A)** Clinical pregnancy rate (CPR). **(B)** Live birth rate (LBR). Direct and indirect evidence were compared to evaluate potential disagreement in the timing-node network.

The key assumptions of network meta-analysis are summarized in **Figure 4** and **Supplementary File 6**. **Supplementary Files 15**–**17** show the results of network connectivity, consistency, and transitivity assessments. Inconsistency signals were mainly observed in acupuncture-modality networks for CPR, LBR, and IR, and in timing-node networks for CPR, OPR, and BPR. No clear inconsistency signal was detected for MR or for the retention-time networks. The expanded transitivity assessment showed that major clinical and methodological effect modifiers were not fully balanced across timing nodes. The COH node included fresh, mixed fresh/frozen, and frozen-thawed embryo-transfer settings, whereas the ET and COH + ET nodes were dominated by fresh or mixed embryo-transfer studies and the luteal-phase node was supported by sparse evidence. Comparator classes also differed across nodes, including inactive controls, nonpenetrating sham acupuncture, superficial sham acupuncture, and sham TEAS.

**Figure 5:**
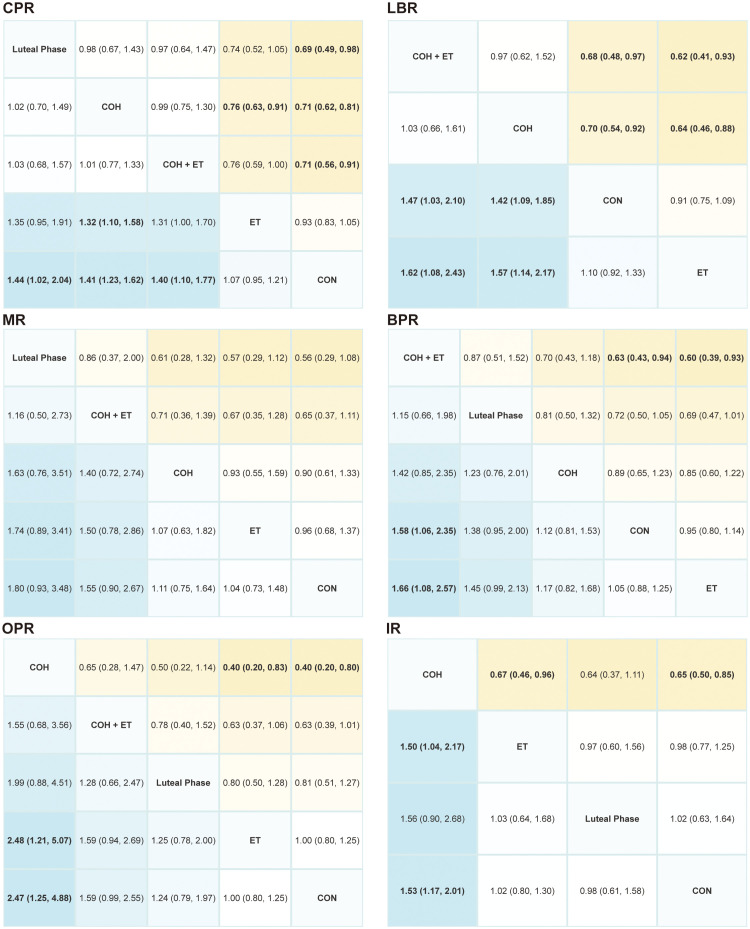
Network meta-analysis league tables of relative effects among acupuncture timing nodes across reproductive outcomes.

As shown in **Figure 5** and **Supplementary Files 18**–**20**, for clinical pregnancy rate, acupuncture during COH showed a higher relative effect than acupuncture limited to ET (RR = 1.32, 95% CI: 1.10-1.58), and acupuncture during COH + ET also favored benefit (RR = 1.31, 95% CI: 1.00-1.70). No clear differences were observed among the luteal phase, COH, and COH + ET nodes. For live birth rate, acupuncture during COH + ET (RR = 1.62, 95% CI: 1.08-2.43) and acupuncture during COH (RR = 1.57, 95% CI: 1.14-2.17) were superior to acupuncture limited to ET. For ongoing pregnancy rate, acupuncture during COH had a higher relative effect than acupuncture limited to ET (RR = 2.48, 95% CI: 1.21-5.07), but its advantage over COH + ET or luteal-phase acupuncture was unclear. For biochemical pregnancy rate, acupuncture during COH + ET was superior to acupuncture limited to ET (RR = 1.66, 95% CI: 1.08-2.57), whereas differences between COH or luteal-phase acupuncture and ET-only acupuncture were unclear. For implantation rate, acupuncture during COH was superior to acupuncture limited to ET (RR = 1.50, 95% CI: 1.04-2.17). Miscarriage rate showed no clear relative differences across intervention timing nodes.

**Supplementary Files 18**–**21** show the network meta-analysis results when acupuncture modalities were analyzed as network nodes. Electroacupuncture improved live birth rate compared with manual acupuncture (RR = 1.53, 95% CI: 1.02-2.31). For implantation rate, electroacupuncture showed higher relative effects than TEAS (RR = 2.20, 95% CI: 1.07-4.52) and manual acupuncture (RR = 2.67, 95% CI: 1.36-5.23). For other outcomes, no stable or clear relative differences were observed among electroacupuncture, TEAS, and manual acupuncture. Analysis of needle-retention time did not identify a consistently optimal node across outcomes. For live birth rate, 25-minute electroacupuncture was superior to 25-minute manual acupuncture (RR = 1.76, 95% CI: 1.04-2.97). For ongoing pregnancy rate, 30-minute manual acupuncture was superior to 25-minute manual acupuncture (RR = 1.33, 95% CI: 1.05-1.69). For implantation rate, 30-minute electroacupuncture was superior to 25-minute manual acupuncture (RR = 3.01, 95% CI: 1.59-5.70), 30-minute manual acupuncture (RR = 2.22, 95% CI: 1.11-4.45), and 30-minute TEAS (RR = 2.28, 95% CI: 1.16-4.51).

### 3.5 Dose-response relationship

**Figure 6:**
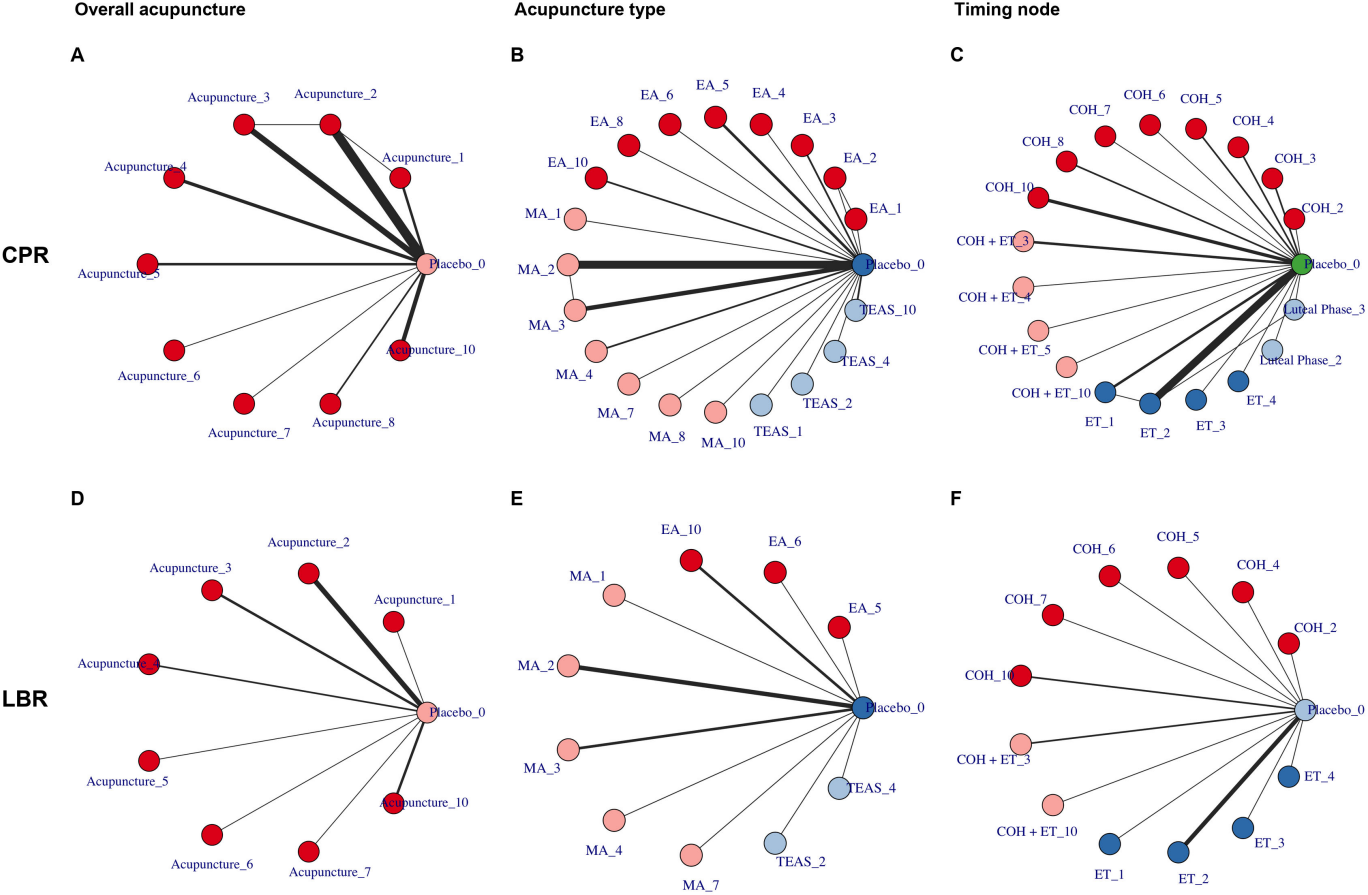
MBNMA network connectivity for CPR and LBR. Panels **(A–C)** show CPR networks for overall acupuncture, acupuncture type, and timing node; panels **(D–F)** show LBR networks.

**Figure 7:**
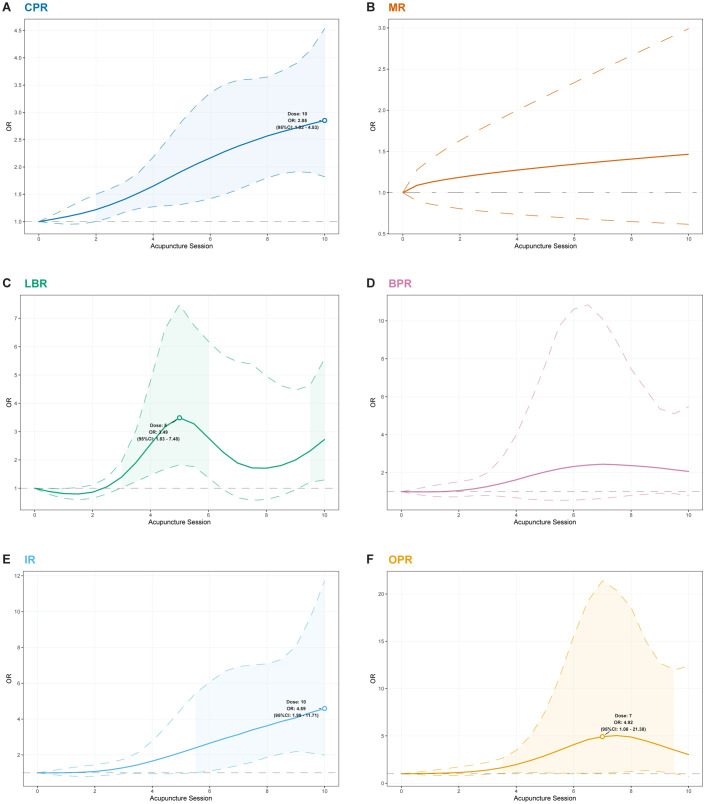
Overall dose-response curves by number of acupuncture sessions. Curves are exploratory model-based estimates and should not be interpreted as treatment recommendations. **(A)** CPR; **(B)** MR; **(C)** LBR; **(D)** BPR; **(E)** IR; **(F)** OPR. Solid lines indicate posterior median ORs, and dashed lines indicate 95% credible intervals, with no acupuncture as the reference. Wald tests for nonlinearity were CPR, P < 0.001; LBR, P = 0.005; BPR, P = 0.17; IR, P < 0.001; and OPR, P = 0.03.

The core outcome networks for CPR and LBR were connected across the relevant dose and treatment-node structures. Connectivity plots for overall acupuncture, acupuncture modality, and intervention timing are presented in **Figure 6**. However, evidence was unevenly distributed across the dose range and became relatively sparse at higher session numbers. **Figure 7** presents the model-predicted dose-response relationships between the number of acupuncture sessions and reproductive outcomes. The predicted effects on clinical pregnancy, live birth, ongoing pregnancy, and implantation rates generally increased with session number. Within the evaluated dose range, the highest model-predicted effects occurred at 10 sessions for clinical pregnancy rate (OR = 2.85, 95% CrI: 1.82–4.53), approximately 5 sessions for live birth rate (OR = 3.49, 95% CrI: 1.83–7.48), approximately 7 sessions for ongoing pregnancy rate (OR = 5.04, 95% CrI: 1.12–20.37), and 10 sessions for implantation rate (OR = 4.59, 95% CrI: 1.99–11.71). These session numbers indicate the highest model-predicted effects rather than confirmed optimal treatment doses. The predicted dose-response pattern for biochemical pregnancy rate was unstable, with most 95% credible intervals including the null. Miscarriage rate showed a possible increase at lower doses, but the estimates were imprecise.

**Figure 8:**
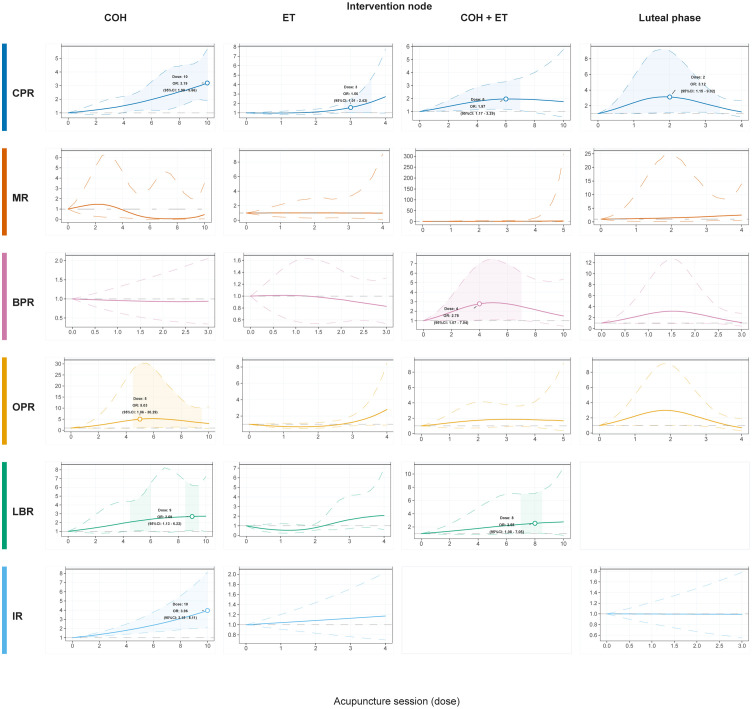
Timing-stratified dose-response curves by number of acupuncture sessions. Curves are exploratory model-based estimates and should not be interpreted as treatment recommendations. Rows show CPR, MR, BPR, OPR, LBR, and IR; columns show COH, ET, COH + ET, and luteal phase. Wald tests for nonlinearity were: CPR, P < 0.001 for COH, P = 0.128 for ET, P = 0.032 for COH + ET, and P = 0.054 for luteal phase; BPR, P = 0.293 for COH, P = 0.705 for ET, P = 0.098 for COH + ET, and P = 0.164 for luteal phase; OPR, P = 0.029 for COH, P = 0.137 for ET, P = 0.170 for COH + ET, and P = 0.154 for luteal phase; LBR, P = 0.030 for COH, P = 0.182 for ET, and P = 0.130 for COH + ET.

**Figure 8** presents the model-predicted dose-response relationships according to intervention timing. For clinical pregnancy rate, all four timing nodes included at least one dose with a 95% credible interval excluding the null. The highest model-predicted effects occurred at 10 sessions during COH (OR = 3.19, 95% CrI: 1.90–5.66), 2 sessions during the luteal phase (OR = 3.12, 95% CrI: 1.15–9.02), 6 sessions during COH + ET (OR = 1.97, 95% CrI: 1.17–3.29), and 3 sessions during ET alone (OR = 1.56, 95% CrI: 1.01–2.43). For live birth rate, the highest predicted effects occurred at 9 COH sessions (OR = 2.69, 95% CrI: 1.13–6.22) and 8 COH + ET sessions (OR = 2.55, 95% CrI: 1.08–7.05). For ongoing pregnancy rate, the highest predicted effect occurred at 5 COH sessions (OR = 5.03, 95% CrI: 1.06–30.29), although the wide credible interval indicated substantial uncertainty. Estimates for the other timing nodes generally favored benefit but remained imprecise. For biochemical pregnancy rate, only the estimate at 4 COH + ET sessions excluded the null (OR = 2.78, 95% CrI: 1.07–7.04), whereas estimates for the other timing nodes were uncertain. For implantation rate, an increasing predicted effect was observed during COH, with the highest estimate at 10 sessions (OR = 3.96, 95% CrI: 2.15–8.11).

**Supplementary Files 22**–**24** present the model-predicted dose-response analyses by acupuncture modality. For clinical pregnancy rate, the highest predicted effects occurred at 10 EA sessions (OR = 3.97, 95% CrI: 1.70–8.83), 7 MA sessions (OR = 3.40, 95% CrI: 1.66–7.43), and 7 TEAS sessions (OR = 2.51, 95% CrI: 1.44–4.63). For live birth rate, the highest predicted effect for MA occurred at 7 sessions (OR = 4.10, 95% CrI: 1.68–11.29), whereas for implantation rate, the highest predicted effect for EA occurred at 10 sessions (OR = 6.38, 95% CrI: 2.42–16.19). No clear modality-specific dose advantage was identified for biochemical pregnancy, ongoing pregnancy, or miscarriage rates.

## 4 Discussion

This study evaluated acupuncture timing nodes with network meta-analysis and explored model-based timing-dose signals across session numbers. In conventional pairwise meta-analysis, acupuncture was associated with statistically higher clinical pregnancy and implantation rates; however, there was insufficient evidence that acupuncture was associated with improvements in live birth, ongoing pregnancy, or biochemical pregnancy rates. Subgroup analyses showed higher estimates for clinical pregnancy, live birth, ongoing pregnancy, and implantation rates with acupuncture during COH, and higher estimates for clinical pregnancy, ongoing pregnancy, and biochemical pregnancy rates with continuous acupuncture during COH and ET. Acupuncture limited to ET showed no clear benefit. Network meta-analysis showed a similar pattern: compared with ET-only acupuncture, acupuncture during COH or COH + ET showed higher relative effect estimates for several reproductive outcomes, although these estimates should be interpreted considering transitivity limitations and timing-dose correlation. Mechanistically, COH can cause supraphysiologic hormonal exposure, embryo-endometrial asynchrony, displacement of the implantation window, and reduced endometrial receptivity (69). Preclinical and indirect clinical evidence has proposed that acupuncture during COH could be related to endometrial receptivity and implantation through possible effects on estrogen-progesterone balance, the implantation window, VEGF/FGF-2-mediated endometrial angiogenesis, and immune or circadian signals (70–74). Nevertheless, these mechanistic explanations should be regarded as biologically plausible rather than definitive clinical evidence, and they provide a rationale for further exploring treatment timing rather than a basis for strong clinical recommendations. The clinical interpretation of these timing findings should therefore remain cautious because the apparent timing effect may partly reflect cumulative treatment exposure; more complex statistical models cannot compensate for low or very-low certainty in the underlying trials.

In this context, the dose-response analysis adds an exploratory layer to the timing findings by examining whether cumulative acupuncture exposure may be associated with reproductive outcomes. The overall MBNMA suggested possible nonlinear associations between session number and some reproductive outcomes, but the apparent higher-effect session ranges varied by outcome and intervention timing and were not consistently supported by dense direct evidence. As the number of sessions increased, estimated effects for clinical pregnancy, live birth, ongoing pregnancy, and implantation rates generally increased. The model produced higher posterior median estimates at selected session numbers, but these model-derived points should be regarded as imprecise predictions rather than treatment-dose recommendations or treatment targets. Timing-specific session patterns also differed across nodes; for example, COH-based protocols generally involved more sessions, whereas luteal-phase evidence was sparse and supported by few comparisons. Because the available data were sparse in several dose ranges and timing and dose were closely related across protocols, curve shapes and estimated maxima may partly reflect model extrapolation and should be interpreted as hypothesis-generating signals rather than clinically established treatment regimens.

A similar level of caution is needed when interpreting differences among acupuncture modalities. We also analyzed acupuncture modality as a network node to explore its influence on reproductive outcomes. Network meta-analysis suggested that electroacupuncture had higher relative effect estimates than manual acupuncture and TEAS for live birth and implantation rates; however, no relative advantage of any acupuncture modality was observed for other reproductive outcomes. Dose-response analyses showed dose-related signals for electroacupuncture, manual acupuncture, and TEAS for clinical pregnancy rate, although the model-predicted higher-effect session ranges differed: approximately 10 sessions for electroacupuncture and approximately 7 sessions for manual acupuncture and TEAS. Electroacupuncture showed a higher model-predicted estimate for implantation rate at approximately 10 sessions, whereas manual acupuncture showed a higher model-predicted effect estimate on live birth rate at approximately 7 sessions. Comparator design is another important source of potential intransitivity. Sham acupuncture, sham TEAS, superficial needling, usual-care controls, and no-treatment controls are not interchangeable, because they differ in physiological stimulation, expectancy effects, patient-practitioner contact, and blinding. These differences may influence relative effects estimated through common-comparator pathways; accordingly, rankings across timing or modality nodes should be regarded as exploratory rather than confirmatory. The comparator subgroup results also showed that estimates could differ between sham or superficial controls and inactive controls, reinforcing the need to interpret pooled and network estimates as average effects across heterogeneous control conditions.

Recent evidence suggests that acupuncture may improve clinical pregnancy rate among women undergoing IVF-ET, but the methodologic quality of included reviews has generally been low, GRADE certainty has been suboptimal, and substantial heterogeneity exists across intervention regimens, treatment timing, comparator type, and outcome definitions (12). An updated meta-analysis by Fu et al. in 2025 showed that acupuncture improved biochemical and clinical pregnancy rates and helped relieve oocyte retrieval-related pain and anxiety, but did not significantly improve live birth rate and identified a potential signal for increased early miscarriage (11). These findings are consistent with the overall results of the present study, in which acupuncture may improve intermediate reproductive outcomes, including clinical pregnancy and implantation rates, whereas evidence for live birth remains uncertain. In addition, the present study found insufficient evidence that acupuncture increased miscarriage rate. We further explored the influence of intervention timing and number of sessions in acupuncture-assisted reproduction. A meta-analysis by Wang et al. on acupuncture timing and dose showed that, in fresh IVF cycles, acupuncture during COH was more likely than acupuncture on the day of embryo transfer to improve clinical pregnancy rate, and high-dose acupuncture was associated with a more favorable live birth rate. That study also noted that the specific relationship between the number of acupuncture sessions and treatment effect remained unclear and that future research should clarify the optimal number and course of treatments (16). A network meta-analysis by Yang et al. also suggested that the timing, course length, and frequency of acupuncture treatment plans affect ART outcomes and that longer courses and more treatment sessions may yield better results (17). A Bayesian network meta-analysis by Bin et al. further showed that acupuncture-related therapies delivered 1–3 cycles before oocyte retrieval were more likely to improve clinical pregnancy rate than limited interventions around embryo transfer (75). Compared with these previous studies, the present analysis extends the evidence by modeling session number as a continuous dose variable and by separately comparing COH, ET, COH + ET, and luteal-phase nodes, while maintaining a conservative interpretation of live birth, comparator heterogeneity, and dose-response findings.

From a methodological perspective, this study has several strengths that help address clinically relevant questions in acupuncture-assisted reproduction. By combining pairwise meta-analysis, network meta-analysis, and model-based dose-response analysis, we evaluated not only the overall effects of acupuncture-related interventions but also the potential influence of intervention timing, acupuncture modality, and number of treatment sessions. Treating session number as a continuous dose variable allowed a more flexible exploratory assessment of nonlinear dose-response patterns than conventional categorical dose comparisons. In addition, the inclusion of multiple reproductive outcomes, including clinical pregnancy, live birth, ongoing pregnancy, implantation, biochemical pregnancy, and miscarriage rates, enabled a more balanced interpretation of potential benefits across early pregnancy establishment and later pregnancy maintenance. These strengths support the value of the analysis as a structured evidence synthesis, but they do not eliminate the limitations inherent in the primary trials and network structure, and they should not be interpreted as increasing the certainty of indirect comparisons or model-based dose estimates beyond that of the underlying evidence.

Several limitations should be considered when interpreting these findings. Although all included studies were randomized controlled trials, several trials had methodological concerns related to blinding, deviations from intended interventions, selective reporting, and the absence of prospectively available protocols, leading to RoB 2 judgments of “some concerns.” More importantly, the certainty of evidence for several clinically important reproductive outcomes, including CPR, LBR, OPR, and MR, was rated as low or very low according to the GRADE assessment. Accordingly, the NMA and MBNMA findings should not be interpreted as definitive clinical recommendations. Rather, the timing-, modality-, dose-response-, ranking-, and adjustment-based results should be regarded as exploratory and hypothesis-generating, because advanced statistical modeling cannot overcome fundamental limitations in the underlying evidence base. Substantial clinical and methodological heterogeneity also remained across ART populations, embryo-transfer types, comparator settings, and acupuncture protocols. Although subgroup analyses, meta-regression, and summaries of potential effect modifiers were performed, these aggregate study-level analyses could not fully account for incompletely reported or unevenly distributed factors, such as infertility etiology, ovarian reserve, IVF versus ICSI treatment, fresh versus frozen embryo transfer, acupoint prescription, treatment frequency, stimulation intensity, acupuncture modality, and comparator type. Because transitivity is a core network assumption that cannot be conclusively verified using aggregate data, residual intransitivity may have influenced indirect comparisons, treatment rankings, and model-based estimates. Possible residual inconsistency in the dose-response MBNMA represents an additional limitation. Furthermore, sparse evidence in certain network nodes and dose ranges reduced the stability of estimates, particularly for live birth, ongoing pregnancy, and miscarriage outcomes. Timing-dose collinearity should also be acknowledged. COH-based protocols generally involved more treatment sessions than ET-only protocols, and the exploratory meta-regression could not reliably disentangle the effects of treatment timing from cumulative acupuncture exposure. Therefore, the observed timing-related associations may reflect timing, dose, or both. Mechanistic interpretations should likewise be made cautiously, because many proposed biological pathways were derived from preclinical studies or non-ART populations rather than from direct clinical evidence in ART settings. The evidentiary basis and interpretive status of the main findings are summarized in **Supplementary File 25**. Finally, although ASReview was used to improve the efficiency of title and abstract screening, AI-assisted screening remains a relatively new approach and may introduce uncertainty regarding the completeness of study identification. To minimize this risk, AI-assisted screening was supplemented by manual verification procedures, full-text review, and additional checks of reference lists and relevant reports. Thus, the likelihood of missing eligible studies was considered low, although it cannot be completely excluded. Future adequately powered and prospectively registered randomized trials should use live birth as the primary endpoint and prespecify acupuncture timing, modality, comparator type, treatment frequency, total number of sessions, and stimulation parameters to determine whether the exploratory timing and dose-response signals observed in this review are clinically meaningful.

## 5 Conclusion

This study suggests that acupuncture as an adjunct to ART may be associated with improvements in selected reproductive outcomes, particularly clinical pregnancy and implantation rates, but evidence for live birth, ongoing pregnancy, and biochemical pregnancy remains uncertain, and no clear increase in miscarriage rate was observed. Timing- and dose-related findings, including more favorable model-based estimates for some COH-related protocols and session ranges, should be regarded as exploratory and hypothesis-generating. Current evidence does not show that any specific treatment timing is preferable and does not support a specific treatment-dose recommendation. Adequately powered, well-designed multicenter randomized trials using live birth as the core outcome are needed to evaluate whether timing, cumulative dose, and modality independently modify clinically meaningful outcomes.

## Data Availability

The original contributions presented in the study are included in the article/**Supplementary Material**. Further inquiries can be directed to the corresponding author.
