# Supplementary material for: Timing and dose of acupuncture as adjuncts to assisted reproduction: a meta-analysis and model-based network meta-analysis
Source: Front Endocrinol (Lausanne). 2026 Jul 9;17:1880225. doi: 10.3389/fendo.2026.1880225 (PMC13391303; doi:10.3389/fendo.2026.1880225)
Supplement: Supplementary file 1 [file DataSheet1.docx]

**Identification of studies via other methods**

**Previous studies**

**Identification of studies via databases and registers**

Previous studies included (n = 0)

Previous reports included (n = 0)

Records identified from*:

Databases (n = 915)

Registers (n = 0)

Records removed before screening:

Duplicate records removed (n = 415)

Marked ineligible by ASReview (n = 333)

Records removed for other reasons (n = 0)

Records identified from:

Websites (n = 0)

Organisations (n = 0)

Citation searching (n = 9)

**Identification**

Total studies included in review

(n = 42)

Reports of total included studies

(n = 42)

Reports assessed for eligibility

(n = 47)

Reports sought for retrieval

(n = 47)

Records screened

(n = 167)

Records excluded**

(n = 120)

Reports not retrieved

(n = 0)

Reports sought for retrieval

(n = 9)

Reports not retrieved

(n = 0)

**Screening**

Reports excluded:

None (n = 0)

Reports excluded:

Wrong outcome (n = 3)

Conference abstract/paper (n = 10)

Research protocol (n = 1)

Reports assessed for eligibility

(n = 9)

New studies included in review

(n = 42)

Reports of new included studies

(n = 42)

**Included**

*Consider, if feasible to do so, reporting the number of records identified from each database or register searched (rather than the total number across all databases/registers).

**If automation tools were used, indicate how many records were excluded by a human and how many were excluded by automation tools.

Source: Page MJ, et al. BMJ 2021;372:n71. doi: 10.1136/bmj.n71.

This work is licensed under CC BY 4.0. To view a copy of this license, visit <https://creativecommons.org/licenses/by/4.0/>
