# Supplementary material for: Timing and dose of acupuncture as adjuncts to assisted reproduction: a meta-analysis and model-based network meta-analysis
Source: Front Endocrinol (Lausanne). 2026 Jul 9;17:1880225. doi: 10.3389/fendo.2026.1880225 (PMC13391303; doi:10.3389/fendo.2026.1880225)
Supplement: Supplementary file 2 [file DataSheet2.docx]

Contents:

[1 Supplemental File 1. Search strategies 3](#_Toc232024966)

[2 Supplemental File 2. Details on the ASReview 10](#_Toc232024967)

[3 Supplemental File 3. Effect modifiers and reporting completeness across included studies 11](#_Toc232024968)

[4 Supplemental File 4. Operational criteria for applying the Cochrane Risk of Bias 2 tool 26](#_Toc232024969)

[**Assessment framework** 26](#_Toc232024970)

[**Bias arising from the randomization process** 26](#_Toc232024971)

[**Bias due to deviations from intended interventions** 26](#_Toc232024972)

[**Bias due to missing outcome data** 26](#_Toc232024973)

[**Bias in measurement of the outcome** 27](#_Toc232024974)

[5 Supplemental File 5. Definition of acupuncture timing nodes 29](#_Toc232024975)

[6 Supplemental File 6. Key assumptions of Network Meta-Analysis 30](#_Toc232024976)

[**Connectivity** 30](#_Toc232024977)

[**Consistency** 37](#_Toc232024978)

[**Transitivity** 40](#_Toc232024979)

[7 Supplemental File 7. Characteristics of the studies included in the meta-analysis. 53](#_Toc232024980)

[8 Supplemental File 8. Risk of bias assessment. 61](#_Toc232024981)

[9 Supplemental File 9. GRADE summary of findings. 62](#_Toc232024982)

[10 Supplemental File 10. Publication bias assessment 69](#_Toc232024983)

[11 Supplemental File 11. Subgroup and meta-regression summary for potential effect modifiers 71](#_Toc232024984)

[12 Supplemental File 12. Exploratory timing-dose meta-regression 78](#_Toc232024985)

[13 Supplemental File 13. Continuous moderator analysis using Meta-CART 83](#_Toc232024986)

[14 Supplemental File 14. Sensitivity analyses for conventional pairwise meta-analysis 85](#_Toc232024987)

[15 Supplemental File 15. Connectivity plots for network meta-analysis by acupuncture type, needle retention time, and acupuncture timing node. 89](#_Toc232024988)

[16 Supplemental File 16. Consistency assessment for network meta-analysis by acupuncture type, needle retention time, and acupuncture timing node. 92](#_Toc232024989)

[17 Supplemental File 17. Transitivity assumption assessment for network meta-analysis by acupuncture type, needle retention time, and acupuncture timing node. 96](#_Toc232024990)

[18 Supplemental File 18. Funnel plots for network meta-analysis by acupuncture type, needle retention time, and acupuncture timing node. 105](#_Toc232024991)

[19 Supplemental File 19. SUCRA ranking plots for network meta-analysis by acupuncture type, needle retention time, and acupuncture timing node. 108](#_Toc232024992)

[20 Supplemental File 20. Forest plots for network meta-analysis by acupuncture type, needle retention time, and acupuncture timing node. 111](#_Toc232024993)

[21 Supplemental File 21. League tables for network meta-analysis by acupuncture type and needle retention time. 117](#_Toc232024994)

[22 Supplemental File 22. SUCRA ranking plots for dose-response network meta-analysis by acupuncture type and acupuncture timing node. 123](#_Toc232024995)

[23 Supplemental File 23. League tables for dose-response network meta-analysis by acupuncture type and acupuncture timing node. 129](#_Toc232024996)

[24 Supplemental File 24. Dose-response curves for dose-response network meta-analysis by acupuncture type. 135](#_Toc232024997)

[25 Supplemental File 25 Classification of principal findings according to evidential basis. 141](#_Toc232024998)

1. **Supplemental File 1. Search strategies**

Supplemental Table 1. PubMed

| Number | Query |
| --- | --- |
| 17 | ((((((Acupuncture Point[Title/Abstract]) OR (Point, Acupuncture[Title/Abstract]) OR (Points, Acupuncture[Title/Abstract]) OR (Acupoints[Title/Abstract]) OR (Acupoint[Title/Abstract])) OR ("Acupuncture Points"[Mesh])) OR (("Acupuncture"[Mesh]) OR (Pharmacopuncture[Title/Abstract]))) OR (((Acupuncture Treatment[Title/Abstract]) OR (Acupuncture Treatments[Title/Abstract]) OR (Treatment, Acupuncture[Title/Abstract]) OR (Therapy, Acupuncture[Title/Abstract]) OR (Pharmacoacupuncture Treatment[Title/Abstract]) OR (Treatment, Pharmacoacupuncture[Title/Abstract]) OR (Pharmacoacupuncture Therapy[Title/Abstract]) OR (Therapy, Pharmacoacupuncture[Title/Abstract]) OR (Acupotomy[Title/Abstract]) OR (Acupotomies[Title/Abstract])) OR ("Acupuncture Therapy"[Mesh]))) AND (("Reproductive Techniques, Assisted"[Mesh]) OR ((Assisted Reproductive Technique[Title/Abstract]) OR (Reproductive Technique, Assisted[Title/Abstract]) OR (Technique, Assisted Reproductive[Title/Abstract]) OR (Techniques, Assisted Reproductive[Title/Abstract]) OR (Assisted Reproductive Technics[Title/Abstract]) OR (Assisted Reproductive Technic[Title/Abstract]) OR (Reproductive Technic, Assisted[Title/Abstract]) OR (Reproductive Technics, Assisted[Title/Abstract]) OR (Technic, Assisted Reproductive[Title/Abstract]) OR (Technics, Assisted Reproductive[Title/Abstract]) OR (Assisted Reproductive Techniques[Title/Abstract]) OR (Reproductive Technology, Assisted[Title/Abstract]) OR (Assisted Reproductive Technologies[Title/Abstract]) OR (Assisted Reproductive Technology[Title/Abstract]) OR (Reproductive Technologies, Assisted[Title/Abstract]) OR (Technologies, Assisted Reproductive[Title/Abstract]) OR (Technology, Assisted Reproductive[Title/Abstract])))) AND ((((((clinical trials as topic[mesh]) OR (clinical trial[pt])) OR (random*[tiab])) OR (random allocation[mesh])) OR (therapeutic use[sh])) OR ((clinical[tiab]) AND (trial[tiab]))) |
| 16 | ((((Acupuncture Point[Title/Abstract]) OR (Point, Acupuncture[Title/Abstract]) OR (Points, Acupuncture[Title/Abstract]) OR (Acupoints[Title/Abstract]) OR (Acupoint[Title/Abstract])) OR ("Acupuncture Points"[Mesh])) OR (("Acupuncture"[Mesh]) OR (Pharmacopuncture[Title/Abstract]))) OR (((Acupuncture Treatment[Title/Abstract]) OR (Acupuncture Treatments[Title/Abstract]) OR (Treatment, Acupuncture[Title/Abstract]) OR (Therapy, Acupuncture[Title/Abstract]) OR (Pharmacoacupuncture Treatment[Title/Abstract]) OR (Treatment, Pharmacoacupuncture[Title/Abstract]) OR (Pharmacoacupuncture Therapy[Title/Abstract]) OR (Therapy, Pharmacoacupuncture[Title/Abstract]) OR (Acupotomy[Title/Abstract]) OR (Acupotomies[Title/Abstract])) OR ("Acupuncture Therapy"[Mesh])) |
| 15 | (((((clinical trials as topic[mesh]) OR (clinical trial[pt])) OR (random*[tiab])) OR (random allocation[mesh])) OR (therapeutic use[sh])) OR ((clinical[tiab]) AND (trial[tiab])) |
| 14 | (clinical[tiab]) AND (trial[tiab]) |
| 13 | ((Acupuncture Point[Title/Abstract]) OR (Point, Acupuncture[Title/Abstract]) OR (Points, Acupuncture[Title/Abstract]) OR (Acupoints[Title/Abstract]) OR (Acupoint[Title/Abstract])) OR ("Acupuncture Points"[Mesh]) |
| 12 | (Acupuncture Point[Title/Abstract]) OR (Point, Acupuncture[Title/Abstract]) OR (Points, Acupuncture[Title/Abstract]) OR (Acupoints[Title/Abstract]) OR (Acupoint[Title/Abstract]) |
| 11 | "Acupuncture Points"[Mesh] |
| 10 | ("Acupuncture"[Mesh]) OR (Pharmacopuncture[Title/Abstract]) |
| 9 | ("Reproductive Techniques, Assisted"[Mesh]) OR ((Assisted Reproductive Technique[Title/Abstract]) OR (Reproductive Technique, Assisted[Title/Abstract]) OR (Technique, Assisted Reproductive[Title/Abstract]) OR (Techniques, Assisted Reproductive[Title/Abstract]) OR (Assisted Reproductive Technics[Title/Abstract]) OR (Assisted Reproductive Technic[Title/Abstract]) OR (Reproductive Technic, Assisted[Title/Abstract]) OR (Reproductive Technics, Assisted[Title/Abstract]) OR (Technic, Assisted Reproductive[Title/Abstract]) OR (Technics, Assisted Reproductive[Title/Abstract]) OR (Assisted Reproductive Techniques[Title/Abstract]) OR (Reproductive Technology, Assisted[Title/Abstract]) OR (Assisted Reproductive Technologies[Title/Abstract]) OR (Assisted Reproductive Technology[Title/Abstract]) OR (Reproductive Technologies, Assisted[Title/Abstract]) OR (Technologies, Assisted Reproductive[Title/Abstract]) OR (Technology, Assisted Reproductive[Title/Abstract])) |
| 8 | ((Acupuncture Treatment[Title/Abstract]) OR (Acupuncture Treatments[Title/Abstract]) OR (Treatment, Acupuncture[Title/Abstract]) OR (Therapy, Acupuncture[Title/Abstract]) OR (Pharmacoacupuncture Treatment[Title/Abstract]) OR (Treatment, Pharmacoacupuncture[Title/Abstract]) OR (Pharmacoacupuncture Therapy[Title/Abstract]) OR (Therapy, Pharmacoacupuncture[Title/Abstract]) OR (Acupotomy[Title/Abstract]) OR (Acupotomies[Title/Abstract])) OR ("Acupuncture Therapy"[Mesh]) |
| 7 | (Acupuncture Treatment[Title/Abstract]) OR (Acupuncture Treatments[Title/Abstract]) OR (Treatment, Acupuncture[Title/Abstract]) OR (Therapy, Acupuncture[Title/Abstract]) OR (Pharmacoacupuncture Treatment[Title/Abstract]) OR (Treatment, Pharmacoacupuncture[Title/Abstract]) OR (Pharmacoacupuncture Therapy[Title/Abstract]) OR (Therapy, Pharmacoacupuncture[Title/Abstract]) OR (Acupotomy[Title/Abstract]) OR (Acupotomies[Title/Abstract]) |
| 6 | "Acupuncture Therapy"[Mesh] |
| 5 | Pharmacopuncture[Title/Abstract] |
| 4 | "Acupuncture"[Mesh] |
| 2 | (Assisted Reproductive Technique[Title/Abstract]) OR (Reproductive Technique, Assisted[Title/Abstract]) OR (Technique, Assisted Reproductive[Title/Abstract]) OR (Techniques, Assisted Reproductive[Title/Abstract]) OR (Assisted Reproductive Technics[Title/Abstract]) OR (Assisted Reproductive Technic[Title/Abstract]) OR (Reproductive Technic, Assisted[Title/Abstract]) OR (Reproductive Technics, Assisted[Title/Abstract]) OR (Technic, Assisted Reproductive[Title/Abstract]) OR (Technics, Assisted Reproductive[Title/Abstract]) OR (Assisted Reproductive Techniques[Title/Abstract]) OR (Reproductive Technology, Assisted[Title/Abstract]) OR (Assisted Reproductive Technologies[Title/Abstract]) OR (Assisted Reproductive Technology[Title/Abstract]) OR (Reproductive Technologies, Assisted[Title/Abstract]) OR (Technologies, Assisted Reproductive[Title/Abstract]) OR (Technology, Assisted Reproductive[Title/Abstract]) |
| 1 | "Reproductive Techniques, Assisted"[Mesh] |

Supplemental Table 2. Web of Science

| # | Query |
| --- | --- |
| 1 | "TS=(Acupuncture OR Electroacupuncture OR Pharmacopuncture) |
| 2 | "TS=(Acupuncture Therapy OR Acupuncture Treatment OR Acupuncture Treatments OR Treatment, Acupuncture OR Therapy, Acupuncture OR Pharmacoacupuncture Treatment OR Treatment, Pharmacoacupuncture OR Pharmacoacupuncture Therapy OR Therapy, Pharmacoacupuncture) |
| 3 | "TS=(Acupuncture Points OR Acupuncture Point OR Point, Acupuncture OR Points, Acupuncture OR Acupoints OR Acupoint) |
| 4 | "#1 OR #2 OR #3 |
| 5 | "TS=(Reproductive Techniques, Assisted) |
| 6 | "TS=((Assisted Reproductive Technique) OR (Reproductive Technique, Assisted) OR (Technique, Assisted Reproductive) OR (Techniques, Assisted Reproductive) OR (Assisted Reproductive Technics) OR (Assisted Reproductive Technic) OR (Reproductive Technic, Assisted) OR (Reproductive Technics, Assisted) OR (Technic, Assisted Reproductive) OR (Technics, Assisted Reproductive) OR (Assisted Reproductive Techniques) OR (Reproductive Technology, Assisted) OR (Assisted Reproductive Technologies) OR (Assisted Reproductive Technology) OR (Reproductive Technologies, Assisted) OR (Technologies, Assisted Reproductive) OR (Technology, Assisted Reproductive)) |
| 7 | "#6 OR #5 |
| 8 | "TS=((Randomized Controlled Trials as Topic OR Clinical Trials, Randomized OR Trials, Randomized Clinical OR Controlled Clinical Trials, Randomized)) |
| 9 | "TS=(Randomized Controlled Trial) |
| 10 | "#9 OR #8 |
| 11 | "#10 AND #7 AND #4 |

Supplemental Table 3. Embase

| No. | Query |
| --- | --- |
| #19 | #3 AND #12 AND #18 |
| #18 | #13 OR #14 OR #15 OR #16 OR #17 |
| #17 | 'trial':ti |
| #16 | 'randomized controlled trial'/exp OR 'randomized controlled trial' OR 'randomized controlled trial':ab,ti |
| #15 | 'controlled clinical trial'/exp OR 'controlled clinical trial' OR 'controlled clinical trial':ab,ti |
| #14 | 'clinical trial'/exp OR 'clinical trial' OR 'clinical trial':ti |
| #13 | random*:ab |
| #12 | #4 OR #5 OR #6 OR #7 OR #8 OR #9 OR #10 OR #11 |
| #11 | 'deqi':ab,ti |
| #10 | meridian:ab,ti OR meridian?:ab,ti |
| #9 | acupoint:ab,ti OR acupoint?:ab,ti |
| #8 | electroacupuncture:ab,ti |
| #7 | 'acupuncture':ab,ti |
| #6 | 'electroacupuncture'/exp OR 'electroacupuncture' OR 'meridians'/exp OR 'meridians' |
| #5 | 'acupuncture therapy'/exp OR 'acupuncture therapy' OR 'acupuncture treatment' OR 'acupuncture treatments' OR 'treatment, acupuncture' OR 'therapy, acupuncture' |
| #4 | 'acupuncture'/exp OR 'acupuncture' OR 'acupuncture points'/exp OR 'acupuncture points' OR 'points, acupuncture' |
| #3 | #1 OR #2 |
| #2 | 'extracorporeal fertilization':ab,ti OR 'fertilization in vitro':ab,ti OR 'in vitro fertilisation':ab,ti OR 'ivf (in vitro fertilization)':ab,ti OR 'testtube baby':ab,ti OR 'in vitro fertilization' |
| #1 | 'in vitro fertilization'/exp |

Supplemental Table 4. Cochrane Library

| D | Search |
| --- | --- |
| #1 | MeSH descriptor: [Reproductive Techniques, Assisted] explode all trees |
| #2 | (Technologies, Assisted Reproductive) OR ( Assisted Reproductive Technologies) OR ( Reproductive Technology, Assisted) OR ( Assisted Reproductive Technology) OR ( Technology, Assisted Reproductive) OR ( Reproductive Technologies, Assisted) OR ( Technics, Assisted Reproductive) OR ( Technique, Assisted Reproductive) OR ( Reproductive Technics, Assisted) OR ( Techniques, Assisted Reproductive) OR ( Reproductive Technique, Assisted) OR ( Assisted Reproductive Techniques) OR ( Assisted Reproductive Technique) OR ( Technic, Assisted Reproductive) OR ( Assisted Reproductive Technic) OR ( Reproductive Technic, Assisted) OR ( Assisted Reproductive Technics):ti,ab,kw |
| #3 | #1 OR #2 |
| #4 | MESH DESCRIPTOR Acupuncture EXPLODE ALL TREES |
| #5 | MESH DESCRIPTOR Acupuncture Therapy EXPLODE ALL TREES |
| #6 | MESH DESCRIPTOR Electroacupuncture EXPLODE ALL TREES |
| #7 | MESH DESCRIPTOR Acupuncture Points EXPLODE ALL TREES |
| #8 | (Acupuncture OR Acupoint* OR Meridian*):ab,ti,kw |
| #9 | (Electroacupuncture OR Electro-acupuncture):ab,ti,kw |
| #10 | (Acupunctur* OR Needling OR Acup* point*):ab,ti,kw |
| #11 | #4 OR #5 OR #6 OR #7 OR #8 OR #9 OR #10 |
| #12 | #3 AND #11 |

Supplemental Table 5. Ovid Medline

| 1 | Reproductive Techniques, Assisted / |
| --- | --- |
| 2 | (“Assisted Reproductive Technique” OR “Reproductive Technique, Assisted” OR “Technique, Assisted Reproductive” OR “Techniques, Assisted Reproductive” OR “Assisted Reproductive Technics” OR “Assisted Reproductive Technic” OR “Reproductive Technic, Assisted” OR “Reproductive Technics, Assisted” OR “Technic, Assisted Reproductive” OR “Technics, Assisted Reproductive” OR “Assisted Reproductive Techniques” OR “Reproductive Technology, Assisted” OR “Assisted Reproductive Technologies” OR “Assisted Reproductive Technology” OR “Reproductive Technologies, Assisted” OR “Technologies, Assisted Reproductive” OR “Technology, Assisted Reproductive”).ab,ti,kw. |
| 3 | 1 or 2 |
| 4 | Acupuncture Points/ or Acupuncture/ or Acupuncture, Ear/ or Acupuncture Therapy/ |
| 5 | ("Acupuncture Treatment" or "Acupuncture Treatments" or "Treatment, Acupuncture" or "Therapy, Acupuncture" or "Pharmacoacupuncture Treatment" or "Treatment, Pharmacoacupuncture" or "Pharmacoacupuncture Therapy" or "Therapy, Pharmacoacupuncture" or "Acupotomy" or "Acupotomies" or "Acupunctures, Ear" or "Ear Acupunctures" or "Acupuncture, Auricular" or "Acupunctures, Auricular" or "Auricular Acupunctures" or "Auricular Acupuncture" or "Ear Acupuncture" or "Acupuncture Point" or "Point, Acupuncture" or "Points, Acupuncture" or "Acupoints" or "Acupoin" or "Pharmacopuncture").ab,ti,kw. |
| 6 | 4 or 5 |
| 7 | (trial or random or randomized controlled trial or controlled clinical or clinical trial).ab,ti,kw. |
| 8 | 3 and 6 and 7 |

Supplemental Table 6. Scopus

| # | Search Query |
| --- | --- |
| 1 | TITLE-ABS-KEY ("Reproductive Techniques, Assisted" ) |
| 2 | TITLE-ABS-KEY ( "“Assisted Reproductive Technique” OR “Reproductive Technique, Assisted” OR “Technique, Assisted Reproductive” OR “Techniques, Assisted Reproductive” OR “Assisted Reproductive Technics” OR “Assisted Reproductive Technic” OR “Reproductive Technic, Assisted” OR “Reproductive Technics, Assisted” OR “Technic, Assisted Reproductive” OR “Technics, Assisted Reproductive” OR “Assisted Reproductive Techniques” OR “Reproductive Technology, Assisted” OR “Assisted Reproductive Technologies” OR “Assisted Reproductive Technology” OR “Reproductive Technologies, Assisted” OR “Technologies, Assisted Reproductive” OR “Technology, Assisted Reproductive”" ) |
| 3 | #1 OR #2 |
| 4 | TITLE-ABS-KEY ( "Acupuncture" OR "Acupuncture Therapy" OR "Acupuncture, Ear" OR "Acupuncture Points" ) |
| 5 | TITLE-ABS-KEY ( "Acupuncture Treatment" OR "Acupuncture Treatments" OR "Treatment, Acupuncture" OR "Therapy, Acupuncture" OR "Pharmacoacupuncture Treatment" OR "Treatment, Pharmacoacupuncture" OR "Pharmacoacupuncture Therapy" OR "Therapy, Pharmacoacupuncture" OR "Acupotomy" OR "Acupotomies" OR "Acupunctures, Ear" OR "Ear Acupunctures" OR "Acupuncture, Auricular" OR "Acupunctures, Auricular" OR "Auricular Acupunctures" OR "Auricular Acupuncture" OR "Ear Acupuncture" OR "Acupuncture Point" OR "Point, Acupuncture" OR "Points, Acupuncture" OR "Acupoints" OR "Acupoin" OR "Pharmacopuncture" ) |
| 6 | #4 OR #5 |
| 7 | TITLE-ABS-KEY ( "Randomized Controlled Trials as Topic" OR "Clinical Trials, Randomized" OR "Trials, Randomized Clinical" OR "Controlled Clinical Trials, Randomized" ) |
| 8 | TITLE-ABS-KEY ( "Randomized Controlled Trial" ) |
| 9 | #7 OR #8 |
| 10 | #3 AND #6 AND #9 |

1. **Supplemental File 2. Details on the ASReview**

ASReview is an AI-powered literature screening tool that integrates active learning and machine learning algorithms. Its efficacy has been validated by developers and implemented in multiple systematic reviews. This tool enhances screening efficiency by dynamically prioritizing records and surfacing the most relevant entries during title/abstract review. The workflow employs a knowledge-guided initialization protocol: human reviewers first label a subset of relevant and irrelevant articles as training data. Machine learning algorithms then iteratively optimize the prioritization sequence based on reviewers' relevance assessments (relevant/irrelevant). Developers validated the tool's performance through four simulation studies, wherein AI screening was retrospectively applied to datasets with pre-identified eligible studies. Results demonstrated that screening only 8–33% of records could identify 95% of eligible studies, with remaining records classified as low-relevance by AI^[1]^. Given the absence of consensus on optimal stopping criteria, we adopted a heuristic strategy: "terminate screening upon sequential identification of a predefined number of irrelevant records," constraining screening to 8–33% of the total. Following initial deduplication in May 2025, 2,642 records were retained, with a stopping threshold set at 1% sequential irrelevance (n=26). A 2024 simulation study (n=27 systematic reviews) reported 85% sensitivity and 69% specificity when applying this 1% threshold across 30% of screened records^[2]^. To ensure comprehensiveness, researcher WJL conducted a manual review of AI-excluded records.

Reference:

[1] Van De Schoot R, De Bruin J, Schram R, et al. An open source machine learning framework for efficient and transparent systematic reviews[J/OL]. Nature Machine Intelligence, 2021, 3(2): 125-133. DOI: 10.1038/s42256-020-00287-7.

[2] Campos D G, Fütterer T, Gfrörer T, et al. Screening smarter, not harder: A comparative analysis of machine learning screening algorithms and heuristic stopping criteria for systematic reviews in educational research[J/OL]. Educational Psychology Review, 2024, 36(1): 19. DOI: 10.1007/s10648-024-09862-5.

1. **Supplemental File 3. Effect modifiers and reporting completeness across included studies**

Supplemental Table 7. Summary of patient, ART-cycle, and intervention-level effect modifiers and reporting completeness across included studies.

*A. Effect modifiers*

| **Study** | **Population** | **Transfer type** | **Special population** | **Ovarian reserve- related status** | **Age** | **BMI** | **Infertility duration** | **Primary infertility proportion** | **Modality** | **Detailed timing** | **Timing node** | **Sessions** | **Frequency** | **Needle-retention/ session duration** | **Acupoints** | **Comparator as reported** | **Comparator class** |
| --- | --- | --- | --- | --- | --- | --- | --- | --- | --- | --- | --- | --- | --- | --- | --- | --- | --- |
| Altutunji et al., 2019 | Women undergoing fresh embryo transfer | Fresh embryo transfer | PCOS | NR | NR | NR | NR | NR | Manual acupuncture | Controlled ovarian hyperstimulation (before oocyte retrieval) | COH | NR | daily | 30 min | LR3, SP6, SP8, ST36, SP10, ST29, LI14, CV04 | blank/wait-list or usual care control | inactive control |
| Andersen et al., 2010 | Women undergoing fresh embryo transfer | Fresh embryo transfer | unselected infertility population | NR | 31 | 22.5 | 2.5 | 70.53 | Manual acupuncture | Before and after embryo transfer (before and after ET) | ET | 2 | daily | 30 min | Before ET: GV20, ST29, PC6, SP8, LR3; After ET: GV20, LI4, SP10, ST36, SP6 | Streitberger nonpenetrating sham acupuncture | nonpenetrating sham acupuncture |
| Craig et al., 2014 | Women undergoing fresh and/or frozen embryo transfer | Fresh and/or frozen embryo transfer | unselected infertility population | NR | 33.1 | NR | NR | NR | Manual acupuncture | Before and after embryo transfer (before and after ET) | ET | 2 | NR | 25 min | NR | blank/wait-list or usual care control | inactive control |
| Cui et al., 2011 | Women undergoing fresh embryo transfer | Fresh embryo transfer | PCOS | NR | NR | NR | NR | NR | Electroacupuncture | Controlled ovarian hyperstimulation (before and during ovarian stimulation) | COH | 5 | NR | 30 min | CV4, CV3, SP6, KI3 | blank/wait-list or usual care control | inactive control |
| Dehghani et al., 2020 | Women undergoing fresh embryo transfer | Fresh embryo transfer | unselected infertility population | NR | 32.25 | 25.73 | 5.4 | 82.13 | Electroacupuncture | Before embryo transfer; Before and after embryo transfer (before ET; before and after ET) | ET | 1; 2 | daily | 25 min | CV3, CV4, SP6, EX-CA1, GV20 | blank/wait-list or usual care control | inactive control |
| Dieterle et al., 2006 | Women undergoing fresh and/or frozen embryo transfer | Fresh and/or frozen embryo transfer | unselected infertility population | NR | 34.92 | 24.32 | 5.36 | 79.6 | Manual acupuncture | Luteal phase (after ET) | Luteal phase | 2 | daily | 30 min | RN6, SP8, SP6, ST36, SJ12, GB32, GB34, LI14 | superficial needling at nonacupoints | superficial sham acupuncture |
| Domar et al., 2009 | Women undergoing fresh embryo transfer | Fresh embryo transfer | unselected infertility population | NR | 36.1 | NR | NR | NR | Manual acupuncture | Before and after embryo transfer (before and after ET) | ET | 2 | NR | 25 min | NR | blank/wait-list or usual care control | inactive control |
| Dong et al., 2024 | Women undergoing fresh embryo transfer | Fresh embryo transfer | unselected infertility population | NR | 29.63 | 22.95 | NR | NR | Manual acupuncture | Before embryo transfer (from oocyte retrieval to before ET) | ET | 4 | once a day | 30 min | Before ET: CV3, CV6, SP6, LR3, ST25, ST29, ST36 | superficial needling at nonacupoints | superficial sham acupuncture |
| Feng et al., 2022 | Women undergoing fresh and/or frozen embryo transfer | Fresh and/or frozen embryo transfer | advanced reproductive age | advanced reproductive age | 31.6 | 22.45 | 3.49 | NR | TEAS | Controlled ovarian hyperstimulation (before and after ET) | Endometrial preparation / pre-FET period | 2 | NR | 30 min | LI3, SP8, ST29, SP10, ST36, KI3, BL23, RN4 | blank/wait-list or usual care control | inactive control |
| Gillerman et al., 2018 | Women undergoing fresh and/or frozen embryo transfer | Fresh and/or frozen embryo transfer | unselected infertility population | NR | 33.94 | 23.5 | NR | 42.11 | Manual acupuncture | Controlled ovarian hyperstimulation and embryo transfer (after ovarian stimulation, before ET, and after ET) | COH + ET | 3 | daily | 25 min | ST29, CV4, CV6, SP6, SP10, SP8, LR3, HT7, PC6, GV20, ST36 | blank/wait-list or usual care control | inactive control |
| Guven et al., 2020 | Women undergoing fresh embryo transfer | Fresh embryo transfer | unselected infertility population | NR | 30.9 | 23.85 | NR | NR | Manual acupuncture | Before and after embryo transfer (1 week before ET, before ET, and after ET) | ET | 3 | NR | 30 min | LI-4, GV-20, CV-3, LR-3, ST-30, SP-8, SP-6, ST-36, SP-9, CV-4 | blank/wait-list or usual care control | inactive control |
| Ho et al., 2009 | Women undergoing fresh embryo transfer | Fresh embryo transfer | PCOS | NR | 35.02 | NR | NR | NR | Electroacupuncture | Controlled ovarian hyperstimulation (before oocyte retrieval) | COH | 4 | daily | 30 min | LR3, SP6, ST28, EX-CA1, RN6, RN4 | blank/wait-list or usual care control | inactive control |
| Isoyama et al., 2012 | Women undergoing fresh embryo transfer | Fresh embryo transfer | unselected infertility population | NR | 34.2 | NR | 4.07 | NR | Manual acupuncture | Controlled ovarian hyperstimulation and embryo transfer (from oocyte retrieval to before ET and after ET) | COH + ET | 4 | twice a week | 25 min | HT7, PC6, CV17, GV20 | superficial needling at nonacupoints | superficial sham acupuncture |
| Jun et al., 2009 | Women undergoing fresh embryo transfer | Fresh embryo transfer | poor ovarian response | poor ovarian response | 34.46 | 22.5 | 6.11 | 11.42 | Electroacupuncture | Controlled ovarian hyperstimulation (before and during ovarian stimulation) | COH | NR | NR | 30 min | LI2, CV4, KI3, SP6 | blank/wait-list or usual care control | inactive control |
| Li et al., 2014 | Women undergoing fresh embryo transfer | Fresh embryo transfer | PCOS | NR | NR | NR | NR | NR | Electroacupuncture | Controlled ovarian hyperstimulation (from ovarian stimulation to before ET) | COH | 5 | once a day | 30 min | GV6, ST36, SP6, PC6, BL23, CV6, EX-CA1 | blank/wait-list or usual care control | inactive control |
| Magarelli et al., 2009 | Women undergoing fresh and/or frozen embryo transfer | Fresh and/or frozen embryo transfer | unselected infertility population | NR | 34.65 | 32.9 | NR | NR | Electroacupuncture | Controlled ovarian hyperstimulation and embryo transfer (before oocyte retrieval, before ET, and after ET) | COH + ET | 10 | daily | 25 min | NR | blank/wait-list or usual care control | inactive control |
| Morin et al., 2017 | Women undergoing fresh and/or frozen embryo transfer | Fresh and/or frozen embryo transfer | unselected infertility population | NR | 34.5 | NR | NR | NR | Manual acupuncture | Before and after embryo transfer (before and after ET) | ET | 2 | NR | 25 min | CV6, SP8, LR3, GV20, ST29, ST36, SP6, SP10, LI4, LR8 | blank/wait-list or usual care control | inactive control |
| Moy et al., 2011 | Women undergoing fresh embryo transfer | Fresh embryo transfer | unselected infertility population | NR | 33.24 | 24.44 | NR | NR | Manual acupuncture | Before and after embryo transfer (before and after ET) | ET | 2 | NR | 25 min | CV6, SP8, LR3, ST29, GV20, ST36, SP6, SP10, LI4 | superficial needling at nonacupoints | superficial sham acupuncture |
| Pang et al., 2025 | Women undergoing fresh embryo transfer | Fresh embryo transfer | PCOS | NR | 30.73 | 28.07 | 4.23 | 45.09 | Electroacupuncture | Controlled ovarian hyperstimulation (before oocyte retrieval) | COH | 6 | daily | 25 min | CV12, CV6, SP10, SP6, SP9, ST25, ST36, ST40 | nonpenetrating sham acupuncture | nonpenetrating sham acupuncture |
| Paulus et al., 2002 | Women undergoing fresh embryo transfer | Fresh embryo transfer | unselected infertility population | NR | 32.45 | NR | NR | NR | Electroacupuncture | Before and after embryo transfer (before and after ET) | ET | 2 | twice a week | 25 min | SP8, LR3, GV20, SP6, SP10, LI4 | blank/wait-list or usual care control | inactive control |
| Peyvandi et al., 2016 | Women undergoing fresh and/or frozen embryo transfer | Fresh and/or frozen embryo transfer | unselected infertility population | NR | NR | NR | NR | NR | Electroacupuncture | Controlled ovarian hyperstimulation and embryo transfer (before and after oocyte retrieval, and before ET) | COH + ET | 3 | daily | 25 min | GV20, CV3, LR3, PC6, SP6, ST29, ST36, LI4 | blank/wait-list or usual care control | inactive control |
| Qian et al., 2015 | Women undergoing fresh embryo transfer | Fresh embryo transfer | unselected infertility population | NR | 31 | 22.71 | 3.66 | NR | Manual acupuncture | Controlled ovarian hyperstimulation (from ovarian stimulation to before ET) | COH | 3 | NR | 30 min | NR | blank/wait-list or usual care control | inactive control |
| Qu et al., 2017 | Women undergoing fresh embryo transfer | Fresh embryo transfer | unselected infertility population | NR | NR | NR | NR | NR | TEAS | Embryo transfer period | ET | 2 | NR | 30 min | SP10, SP8, LR3, ST36, EX-CA1, RN4, PC6, RN12 | blank/wait-list or usual care control | inactive control |
| Rashidi et al., 2013 | Women undergoing fresh embryo transfer | Fresh embryo transfer | PCOS | NR | 31.57 | 26.96 | 9.25 | 23.5 | Electroacupuncture | Controlled ovarian hyperstimulation and embryo transfer (during pituitary downregulation, before ovulation, before oocyte retrieval, before ET, and after ET) | COH + ET | 5 | daily | 30 min | SP6, LR3, LI4, GV20, CV4, ST36, SP10, PC6, ST29 | blank/wait-list or usual care control | inactive control |
| Seto et al., 2017 | Women undergoing fresh and/or frozen embryo transfer | Fresh and/or frozen embryo transfer | unselected infertility population | NR | 34.7 | NR | 4.65 | NR | Manual acupuncture | Before and after embryo transfer (before and after ET) | ET | 2 | NR | 25 min | PC6, SP8, LR3, GV20, ST29, ST36, SP6, SP10, LI4 | Streitberger nonpenetrating sham acupuncture | nonpenetrating sham acupuncture |
| Shen et al., 2022 | Women undergoing frozen-thawed embryo transfer | Frozen-thawed embryo transfer | diminished ovarian reserve | diminished ovarian reserve | NR | NR | NR | NR | Electroacupuncture | Controlled ovarian hyperstimulation (COH) | Endometrial preparation / pre-FET period | NR | NR | 30 min | LI3, BL17, BL23, GV4, BL32, BL33, ST25, CV6, CV4 | blank/wait-list or usual care control | inactive control |
| Shuai et al., 2019 | Women undergoing fresh embryo transfer | Fresh embryo transfer | recurrent implantation failure | NR | 31.41 | 22.2 | 5.4 | 75.4 | TEAS | Controlled ovarian hyperstimulation (from oocyte retrieval to before ET) | COH | NR | daily | 30 min | LI1, SP6, CV3, CV4 | mock TEAS | sham TEAS |
| Smith et al., 2006 | Women undergoing fresh embryo transfer | Fresh embryo transfer | unselected infertility population | NR | 36 | 25.71 | NR | NR | Manual acupuncture | Controlled ovarian hyperstimulation and embryo transfer (from oocyte retrieval to before ET and after ET) | COH + ET | 3 | NR | 25 min | NR | Streitberger nonpenetrating sham acupuncture | nonpenetrating sham acupuncture |
| Smith et al., 2018 | Women undergoing fresh embryo transfer | Fresh embryo transfer | unselected infertility population | NR | 35.45 | 25.9 | NR | NR | Manual acupuncture | Controlled ovarian hyperstimulation and embryo transfer (from oocyte retrieval to before ET and after ET) | COH + ET | 3 | NR | 25 min | ST-29, CV-4, CV-6, SP-6, SP-10, SP-8, LR-3, HT-7, PC-6, GV-20, ST-36 | Streitberger nonpenetrating sham acupuncture | nonpenetrating sham acupuncture |
| So et al., 2009 | Women undergoing fresh embryo transfer | Fresh embryo transfer | unselected infertility population | NR | 36 | 21.65 | 4 | 61.44 | Manual acupuncture | Before and after embryo transfer (before and after ET) | ET | 2 | NR | 25 min | Before ET: PC6 (Neiguan), SP8 (Diji), LR3 (Taichong), GV20 (Baihui) and ST29 (Guilai); After ET: ST36 (Zusanli), SP6 (Sanyinjiao), SP10 (Xuehai) and LI4 (Hegu) | Streitberger nonpenetrating sham acupuncture | nonpenetrating sham acupuncture |
| So et al., 2010 | Women undergoing frozen-thawed embryo transfer | Frozen-thawed embryo transfer | unselected infertility population | NR | 35 | 21.75 | 5 | 38.06 | Manual acupuncture | After embryo transfer (after ET) | Luteal phase | 1 | daily | 25 min | After ET: ST36 (Zusanli), SP6 (Sanyinjiao), SP10 (Xuehai) and LI4 (Hegu) | Streitberger nonpenetrating sham acupuncture | nonpenetrating sham acupuncture |
| Westergaard et al., 2006 | Women undergoing fresh embryo transfer | Fresh embryo transfer | unselected infertility population | NR | 37 | 22.73 | 3.73 | 41.03 | Manual acupuncture | Before and after embryo transfer; Luteal phase (before and after ET; before and after ET, with an additional session 2 days later) | Luteal phase | 2; 3 | daily | 25 min | SP6, SP8, SP10, ST36, ST29, LI4, LR3, PC6, GV20, CV3 | blank/wait-list or usual care control | inactive control |
| Wu et al., 2019 | Women undergoing fresh embryo transfer | Fresh embryo transfer | unselected infertility population | NR | 32.75 | NR | 3.54 | 51.51 | Electroacupuncture | Controlled ovarian hyperstimulation (before ET) | COH | NR | daily | 25 min | CV3, CV6, ST29, SP6, LI4 | nonpenetrating sham acupuncture | nonpenetrating sham acupuncture |
| Wu et al., 2022 | Women undergoing fresh embryo transfer | Fresh embryo transfer | PCOS | NR | 29.35 | 22.92 | 3.35 | NR | Manual acupuncture | Controlled ovarian hyperstimulation (COH) | COH | 10 | daily | 25 min | CV4, CV6, CV3, EX-CA1, GV4, GV3, BL23, BL32 | blank/wait-list or usual care control | inactive control |
| Xia et al., 2023 | Women undergoing fresh and/or frozen embryo transfer | Fresh and/or frozen embryo transfer | advanced reproductive age | advanced reproductive age | 37.51 | 22.64 | 3.59 | NR | Manual acupuncture | Controlled ovarian hyperstimulation (before oocyte retrieval) | Endometrial preparation / pre-FET period | NR | daily | 30 min | EX-CA1, RN4, SP6, CV3 | Streitberger nonpenetrating sham acupuncture | nonpenetrating sham acupuncture |
| Xiang et al., 2021 | Women undergoing fresh and/or frozen embryo transfer | Fresh and/or frozen embryo transfer | unselected infertility population | NR | 28.8 | 27.7 | 2.95 | 50 | Electroacupuncture | Controlled ovarian hyperstimulation (before oocyte retrieval) | Endometrial preparation / pre-FET period | NR | NR | 25 min | NR | nonpenetrating sham acupuncture | nonpenetrating sham acupuncture |
| Xu et al., 2022 | Women undergoing frozen-thawed embryo transfer | Frozen-thawed embryo transfer | unselected infertility population | NR | 30.5 | 23.2 | 3.1 | NR | Manual acupuncture | Controlled ovarian hyperstimulation (COH) | Endometrial preparation / pre-FET period | NR | every other day | 30 min | GV20, GV4, BL17, CV4, CV6 | blank/wait-list or usual care control | inactive control |
| Zhai et al., 2022 | Women undergoing fresh and/or frozen embryo transfer | Fresh and/or frozen embryo transfer | unselected infertility population | NR | 31.23 | 21.82 | 2.3 | NR | TEAS | Controlled ovarian hyperstimulation (before oocyte retrieval) | Endometrial preparation / pre-FET period | 10 | once a day | 30 min | RN4, RN3, SP6, EX-CA1, KI13 | mock TEAS | sham TEAS |
| Zheng et al., 2015 | Women undergoing fresh embryo transfer | Fresh embryo transfer | diminished ovarian reserve | diminished ovarian reserve | 36.39 | 23.9 | 4.57 | 38.23 | TEAS | Controlled ovarian hyperstimulation (before oocyte retrieval) | COH | NR | once a day | 30 min | LI2, RN4, RN3, SP6, EX-CA1, ST25, BL23, GV3, GV4 | mock TEAS | sham TEAS |
| Zhong et al., 2017 | Women undergoing fresh and/or frozen embryo transfer | Fresh and/or frozen embryo transfer | unselected infertility population | NR | 31.58 | 21.8 | 4.92 | 41.51 | TEAS | Before embryo transfer (before ET) | ET | 1 | NR | 30 min | NR | blank/wait-list or usual care control | inactive control |
| Zhong et al., 2023 | Women undergoing fresh and/or frozen embryo transfer | Fresh and/or frozen embryo transfer | poor ovarian response | poor ovarian response | 38.05 | 22.41 | 3.69 | 50 | Electroacupuncture | Controlled ovarian hyperstimulation (COH) | Endometrial preparation / pre-FET period | 10 | NR | 25 min | NR | blank/wait-list or usual care control | inactive control |
| Zhou et al., 2016 | Women undergoing fresh embryo transfer | Fresh embryo transfer | unselected infertility population | NR | 35 | NR | 6.14 | NR | Electroacupuncture | Controlled ovarian hyperstimulation (COH) | COH | 10 | NR | 30 min | NR | blank/wait-list or usual care control | inactive control |

*B. Reporting summary*

| **Variable** | **Reported** | **NR** | **Total studies** |
| --- | --- | --- | --- |
| Population | 42 | 0 | 42 |
| ART procedure | 36 | 6 | 42 |
| Transfer type | 42 | 0 | 42 |
| Special population | 42 | 0 | 42 |
| Infertility etiology / diagnostic restriction | 36 | 6 | 42 |
| Ovarian reserve-related status | 6 | 36 | 42 |
| Age | 36 | 6 | 42 |
| BMI | 27 | 15 | 42 |
| Infertility duration | 24 | 18 | 42 |
| Primary infertility proportion | 16 | 26 | 42 |
| Modality | 42 | 0 | 42 |
| Detailed timing | 42 | 0 | 42 |
| Timing node | 42 | 0 | 42 |
| Sessions | 33 | 9 | 42 |
| Frequency | 23 | 19 | 42 |
| Needle-retention/session duration | 42 | 0 | 42 |
| Acupoints | 33 | 9 | 42 |
| Deqi/manual stimulation reported | 18 | 24 | 42 |
| Electrical or TEAS parameters | 11 | 31 | 42 |
| Comparator as reported | 42 | 0 | 42 |
| Comparator class | 42 | 0 | 42 |

Note: NR, not reported. Variables with sufficient and consistent reporting were included in subgroup analyses, meta-regression, or meta-CART. Variables with sparse or inconsistent reporting were summarized descriptively and considered in the interpretation of heterogeneity and certainty of evidence. Because moderator analyses were based on aggregate study-level data and were not adjusted for multiplicity, statistically significant findings should be interpreted as exploratory.

1. **Supplemental File 4. Operational criteria for applying the Cochrane Risk of Bias 2 tool**

**Assessment framework**

Risk of bias was assessed using the Cochrane Risk of Bias 2 tool for individually randomized, parallel-group trials (version 22 August 2019). The assessment focused on the effect of assignment to intervention and was conducted at the result level for biochemical pregnancy rate, clinical pregnancy rate, implantation rate, ongoing pregnancy rate, miscarriage rate, and live birth rate. Two reviewers independently evaluated each eligible result using information from trial reports, supplementary materials, protocols, trial registries, statistical analysis plans, and other available sources, with disagreements resolved through discussion or consultation with a third reviewer. Any departure from an algorithm-generated judgment required an explicit methodological justification. The following operational criteria were used to support the consistent application of RoB 2 to acupuncture trials in assisted reproduction and did not replace the official signalling questions or decision algorithms.

**Bias arising from the randomization process**

This domain considered whether the allocation sequence was random, whether allocation was adequately concealed until participants were enrolled and assigned, and whether baseline differences suggested a problem with the randomization process. A result was judged to be at low risk when an appropriate random sequence-generation method and adequate allocation concealment were reported and no important baseline imbalance suggested failure of randomization. Appropriate methods included computer-generated sequences, random-number tables, central randomization, or appropriately implemented drawing of lots. Some concerns were assigned when the study was described as randomized but sequence generation or allocation concealment was insufficiently reported, when concealment procedures were unclear, or when minor baseline imbalances could plausibly have occurred by chance. High risk was assigned when a non-random or predictable allocation method was used, allocation could have been anticipated or manipulated, concealment was clearly inadequate, or substantial baseline imbalances strongly suggested a failure of randomization. Relevant baseline characteristics included age, body mass index, infertility duration and cause, previous assisted-reproduction history, ovarian reserve indicators, and reproductive hormone levels.

**Bias due to deviations from intended interventions**

This domain was assessed for the effect of assignment to intervention and considered whether participants or intervention providers were aware of allocation, whether awareness resulted in deviations from the intended interventions, whether such deviations differed between groups and were likely to affect the outcome, and whether an appropriate assignment-based analysis was used. Because blinding of acupuncture practitioners is generally infeasible, lack of practitioner blinding alone was not considered sufficient to indicate high risk. A result was judged to be at low risk when interventions were delivered substantially as intended, important crossover, contamination, unequal co-interventions, or differential clinical management were absent, and participants were analyzed according to their randomized groups. Some concerns were assigned when adherence, contamination, crossover, co-interventions, or post-randomization exclusions were incompletely reported, or when the potential effect of deviations was uncertain. High risk was assigned when important trial-related deviations differed systematically between groups and were likely to affect the reproductive outcome, or when inappropriate per-protocol or as-treated analyses materially compromised the randomized comparison.

**Bias due to missing outcome data**

This domain considered the amount and reasons for missing outcome data, differences in missingness between groups, whether missingness was likely to depend on the true outcome value, and whether appropriate methods were used to address missing data. No fixed percentage threshold was used as the sole basis for judgment; instead, the potential influence of missing data was evaluated according to event frequency, group size, reasons for missingness, between-group imbalance, and the likely outcomes of participants with missing data. Particular attention was paid to cycle cancellation, failure to retrieve oocytes, fertilization failure, absence of transferable embryos, failure to undergo embryo transfer, withdrawal after randomization, loss to pregnancy follow-up, and unavailable live-birth or miscarriage information. A result was judged to be at low risk when outcome data were available for all or nearly all randomized participants, missingness was unlikely to depend on the true outcome, or appropriate analyses indicated that missing data were unlikely to materially affect the estimate. Some concerns were assigned when some data were missing and the relationship between missingness and the true outcome could not be excluded, or when post-randomization exclusions and reasons for missingness were insufficiently reported. High risk was assigned when missingness was likely to depend on the true outcome, differed importantly between groups, involved selective exclusion of participants with potentially different reproductive outcomes, and was not adequately addressed by appropriate analysis or sensitivity assessment.

**Bias in measurement of the outcome**

This domain considered whether outcome measurement was appropriate and consistent across intervention groups, whether outcome assessors were aware of intervention assignment, and whether such awareness was likely to influence outcome assessment. The reproductive outcomes included in this review were generally objective and were commonly determined using biochemical testing, ultrasonography, pregnancy follow-up, pregnancy-loss records, or delivery records; therefore, lack of participant or practitioner blinding alone was not considered sufficient to indicate measurement bias. A result was judged to be at low risk when appropriate and consistent outcome definitions, thresholds, time points, and measurement procedures were applied across groups and outcome ascertainment was unlikely to be influenced by knowledge of allocation. Some concerns were assigned when outcome definitions or measurement procedures were insufficiently described, when assessors were aware of assignment and some judgment was involved in determining the outcome, or when consistency of assessment across groups was unclear. High risk was assigned when measurement methods, outcome definitions, diagnostic thresholds, or follow-up procedures differed systematically between groups, when allocation awareness was likely to influence assessment, or when errors in the numerator or denominator were likely to materially bias the estimated intervention effect.

**Bias in selection of the reported result**

This domain considered whether the reported numerical result was selected on the basis of its direction, magnitude, or statistical significance from multiple eligible outcome definitions, measurement time points, denominators, or statistical analyses. When available, trial protocols, registry records, statistical analysis plans, conference abstracts, and earlier reports were compared with the published article, with particular attention paid to alternative definitions of pregnancy outcomes, different gestational time points, different analysis denominators, and the use of intention-to-treat, modified intention-to-treat, per-protocol, adjusted, or unadjusted analyses. A result was judged to be at low risk when the reported outcome definition, time point, denominator, and analysis were consistent with a prespecified protocol or analysis plan, or when there was sufficient evidence that selection based on the findings was unlikely. Some concerns were assigned when no protocol or sufficiently detailed analysis plan was available and multiple eligible measurements or analyses were possible, but there was no direct evidence of selective reporting. High risk was assigned when prespecified outcomes or analyses were changed without adequate justification, unfavorable or non-significant results were suppressed, outcome switching was identified, or there was direct evidence that a particular numerical result had been selected according to its statistical significance, magnitude, or direction. Selective non-reporting of an entire outcome was considered separately at the evidence-synthesis level.

**Overall risk-of-bias judgment**

The overall risk-of-bias judgment was determined separately for each assessed result and was based on the five domain-level judgments. A result was classified as low risk when all five domains were judged to be at low risk. A result was classified as having some concerns when at least one domain raised some concerns, no domain was judged to be at high risk, and the combined concerns did not substantially reduce confidence in the result. A result was classified as high risk when at least one domain was judged to be at high risk or when concerns across multiple domains collectively substantially reduced confidence in the result. For each judgment, the reviewers recorded the information sources, responses to the signalling questions, supporting evidence, algorithm-generated judgment, final judgment, and the rationale for any departure from the algorithm. The same decision rules were applied across studies, although separate judgments were made when risk of bias differed by outcome, time point, outcome definition, denominator, or analysis.

1. **Supplemental File 5. Definition of acupuncture timing nodes**

Acupuncture timing was classified according to the stage of the assisted reproductive technology cycle during which the intervention was delivered. Four timing nodes were predefined: controlled ovarian hyperstimulation (COH), embryo transfer (ET), COH plus ET, and luteal phase.

- 1. COH was defined as acupuncture administered mainly during the controlled ovarian hyperstimulation period, from the initiation of ovarian stimulation to before or around oocyte retrieval, without additional acupuncture around embryo transfer or during the luteal phase.
  2. ET was defined as acupuncture administered mainly around embryo transfer, including before, after, or on the day of embryo transfer. Studies in which acupuncture was limited to the peri-transfer period were classified as ET.
  3. COH + ET was defined as acupuncture administered during both the controlled ovarian hyperstimulation period and the embryo transfer period. Studies were classified as COH plus ET when acupuncture began during ovarian stimulation and continued before, on, or after embryo transfer.
  4. Luteal phase was defined as acupuncture administered mainly after embryo transfer during the luteal support or implantation period, from after embryo transfer to the pregnancy test. Studies in which acupuncture was repeatedly delivered after embryo transfer rather than only on the day of embryo transfer were classified as luteal phase interventions.

1. **Supplemental File 6. Key assumptions of Network Meta-Analysis**

There are three key assumptions for conducting a network meta-analysis (NMA) and model-based network meta-analysis (MBNMA): (1) network connectivity, (2) consistency in the data, and (3) transitivity. We assessed these assumptions for each reproductive outcome at the overall acupuncture level, acupuncture type level, and acupuncture timing-node level.

**Connectivity**

Connectivity is a key assumption in NMA and MBNMA because insufficient direct comparisons may reduce statistical power and make indirect estimates less reliable. We visually assessed network connectivity for each outcome and analysis level. The available networks connected the intervention nodes through the common comparator and direct treatment contrasts, as shown below.

**A. Overall acupuncture**


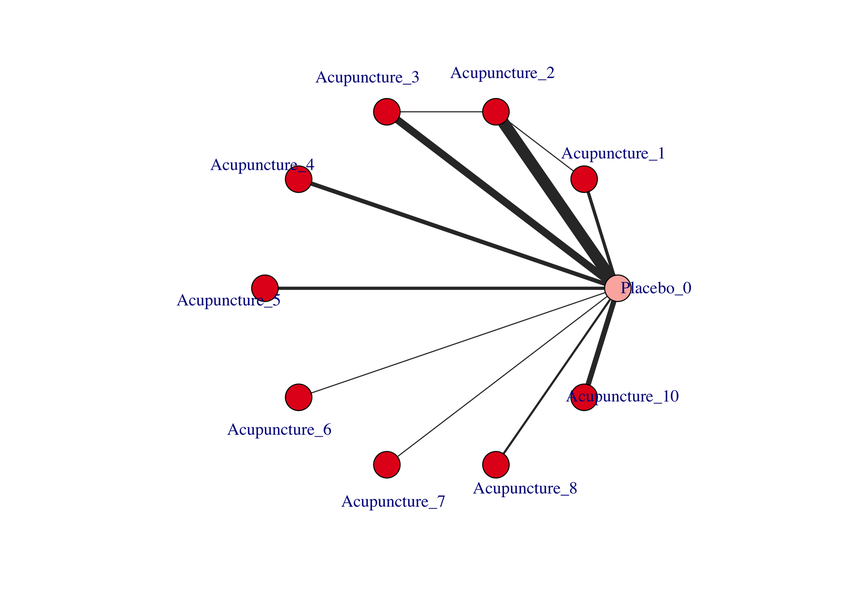


**B. Acupuncture type**


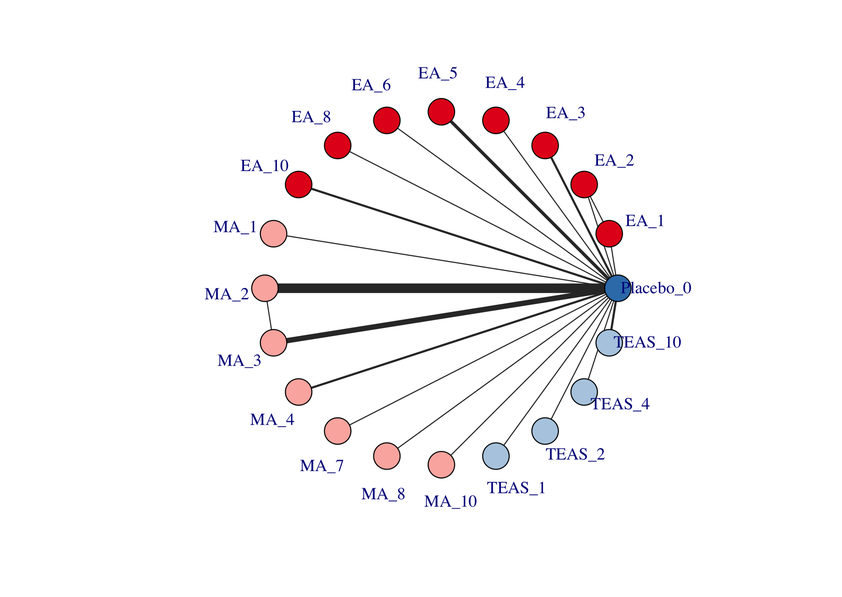


**C. Acupuncture timing nodes**


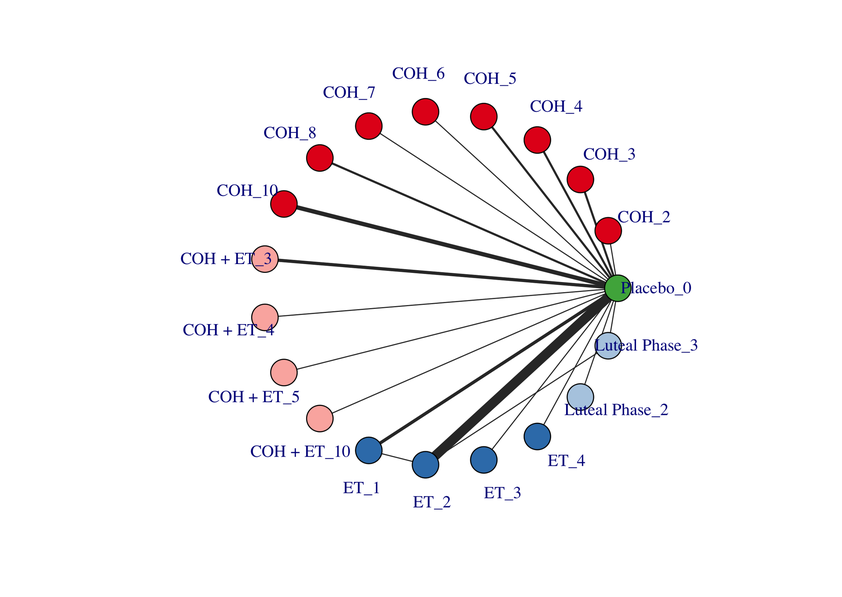


**A. Overall acupuncture**


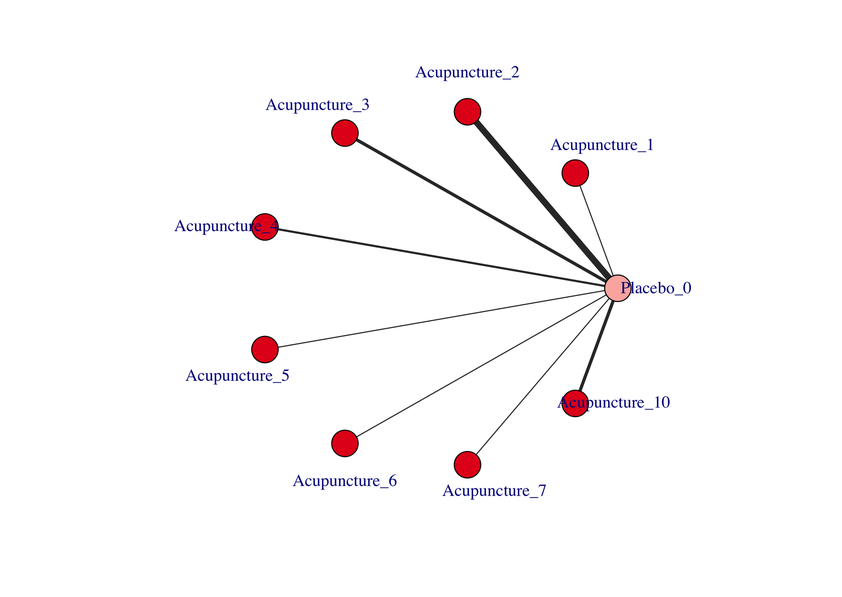


**B. Acupuncture type**


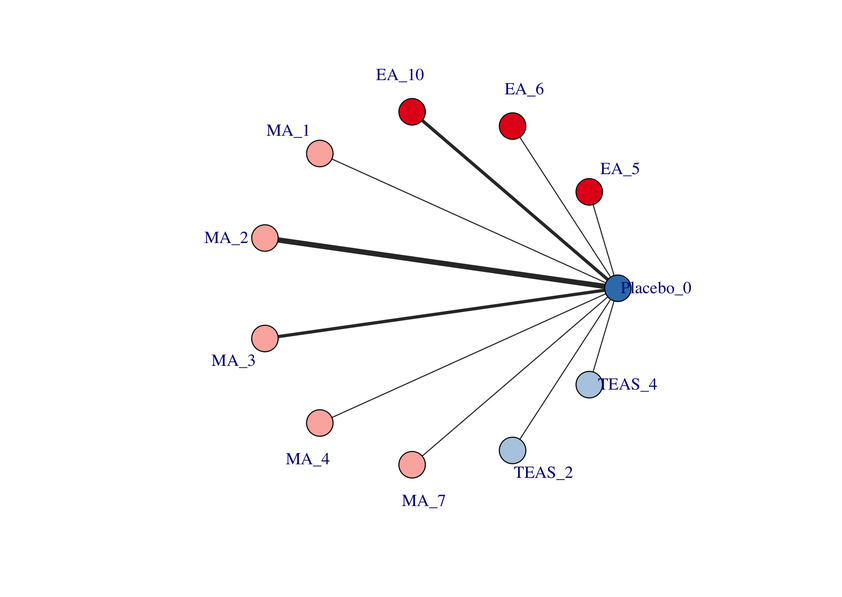


**C. Acupuncture timing nodes**


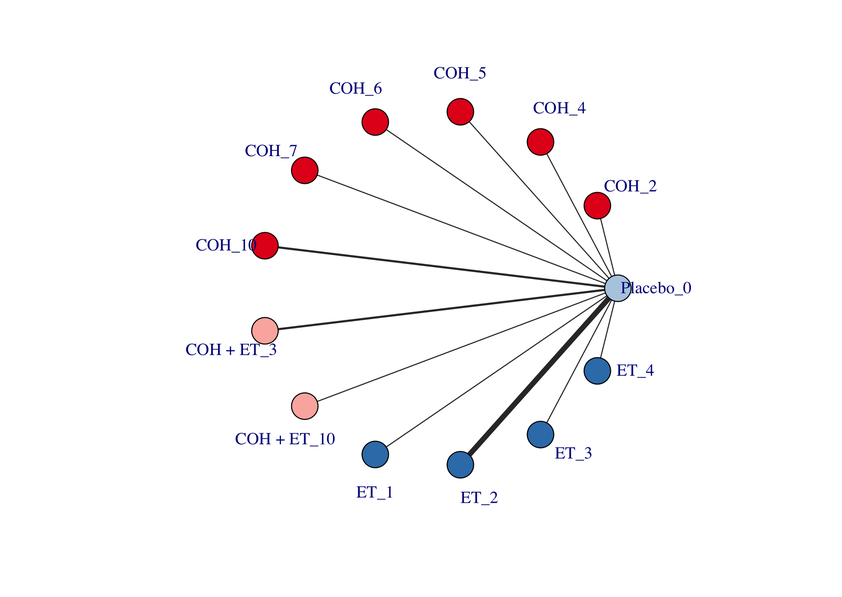


Supplemental Figure 1. Connectivity plots for OPR (ongoing pregnancy rate). A: overall acupuncture; B: acupuncture type; C: acupuncture timing nodes.

**A. Overall acupuncture**


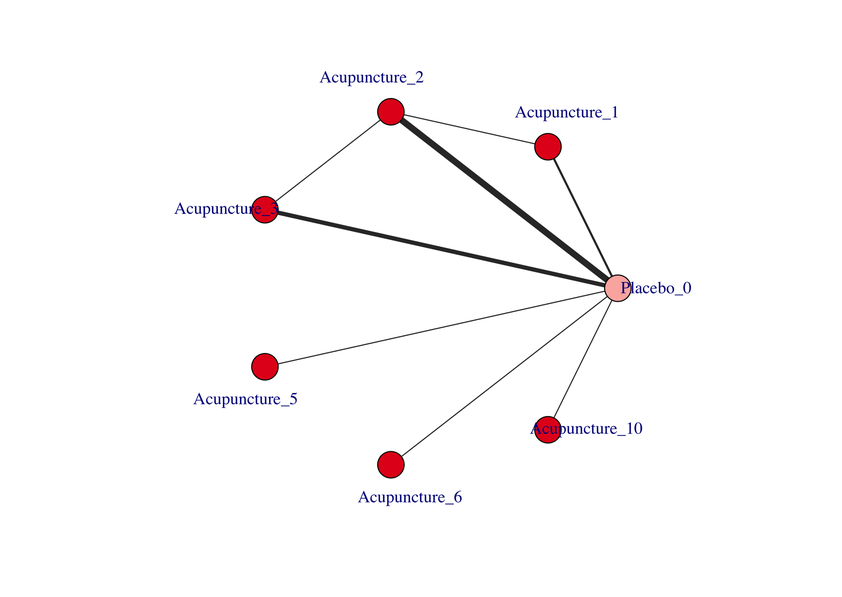


**B. Acupuncture type**


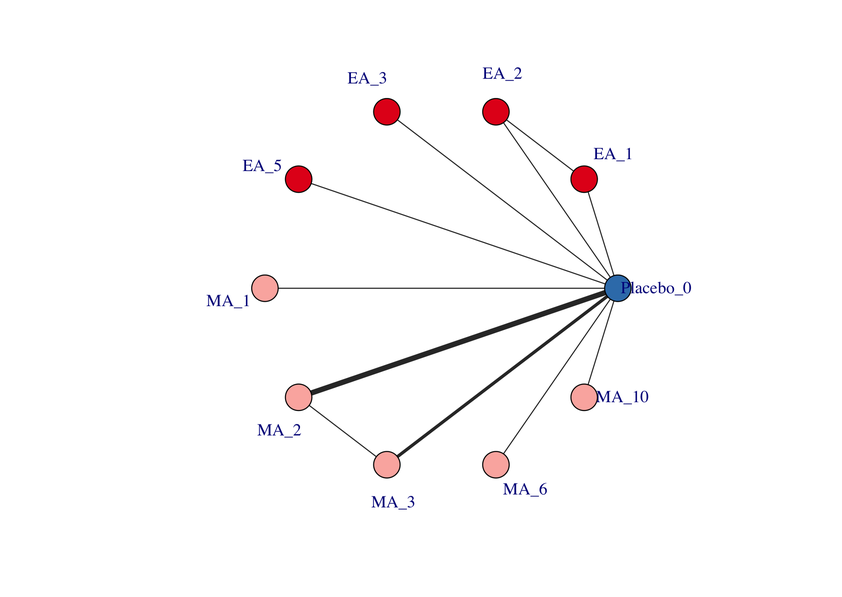


**C. Acupuncture timing nodes**


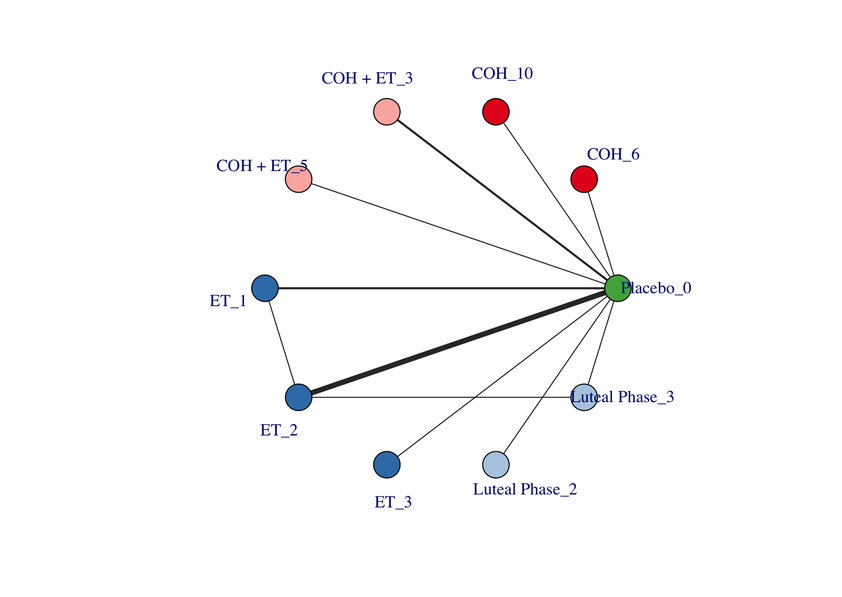


Supplemental Figure 2. Connectivity plots for BPR (biochemical pregnancy rate). A: overall acupuncture; B: acupuncture type; C: acupuncture timing nodes.

**A. Overall acupuncture**


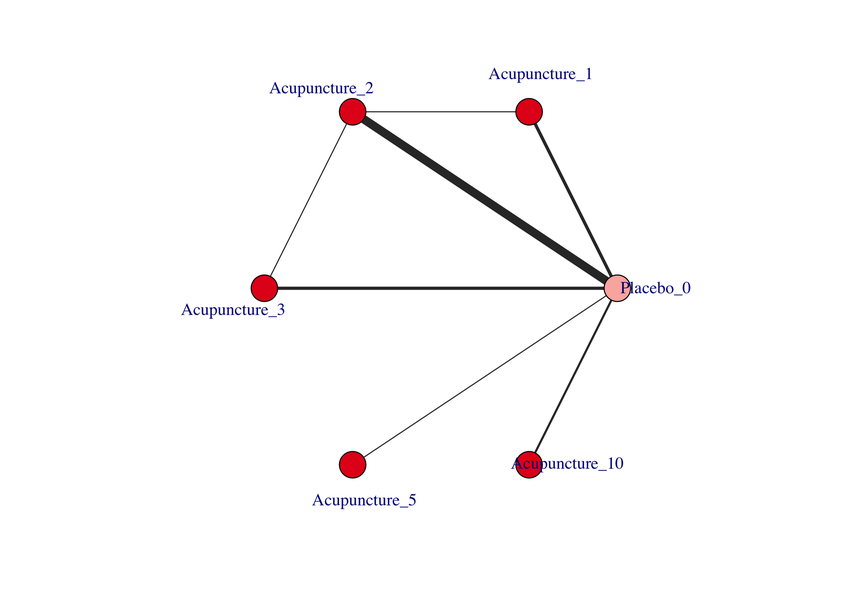


**B. Acupuncture type**


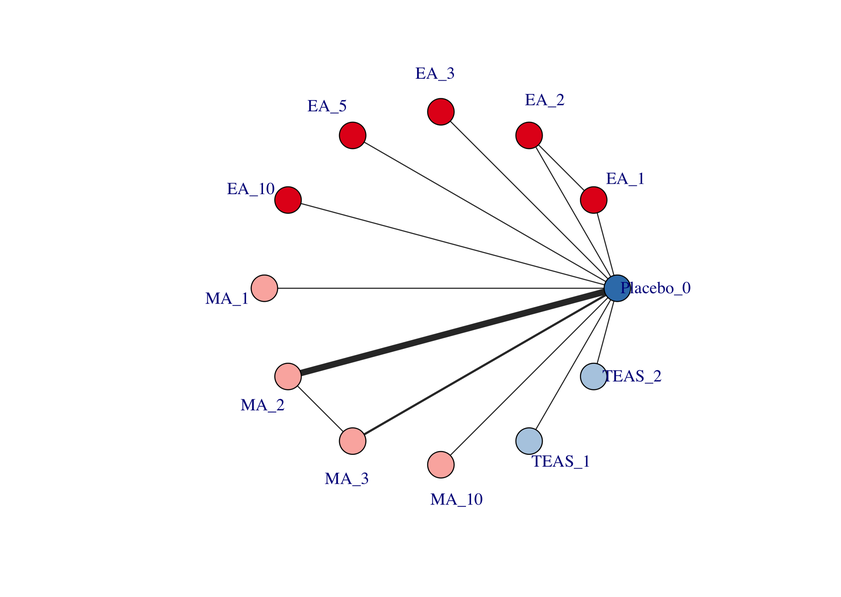


**C. Acupuncture timing nodes**


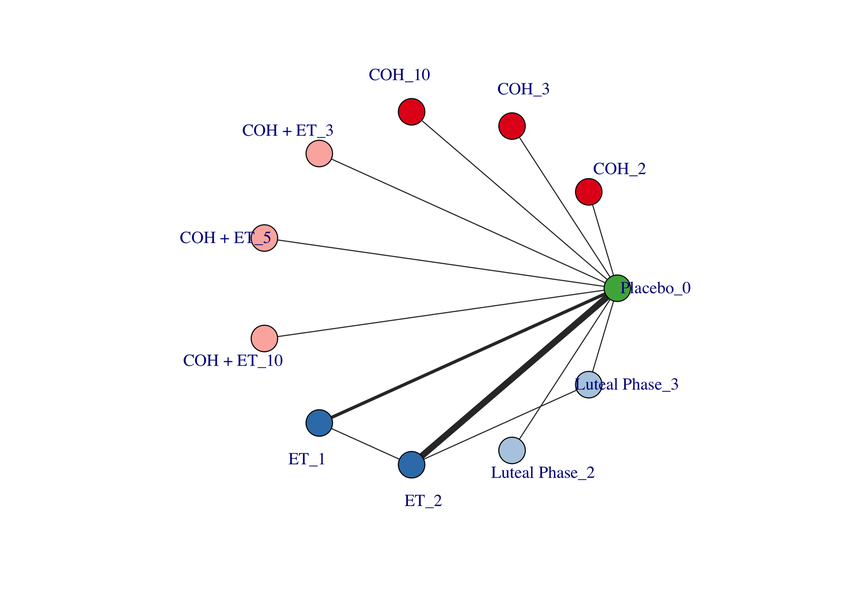


Supplemental Figure 3. Connectivity plots for IR (implantation rate). A: overall acupuncture; B: acupuncture type; C: acupuncture timing nodes.

**A. Overall acupuncture**


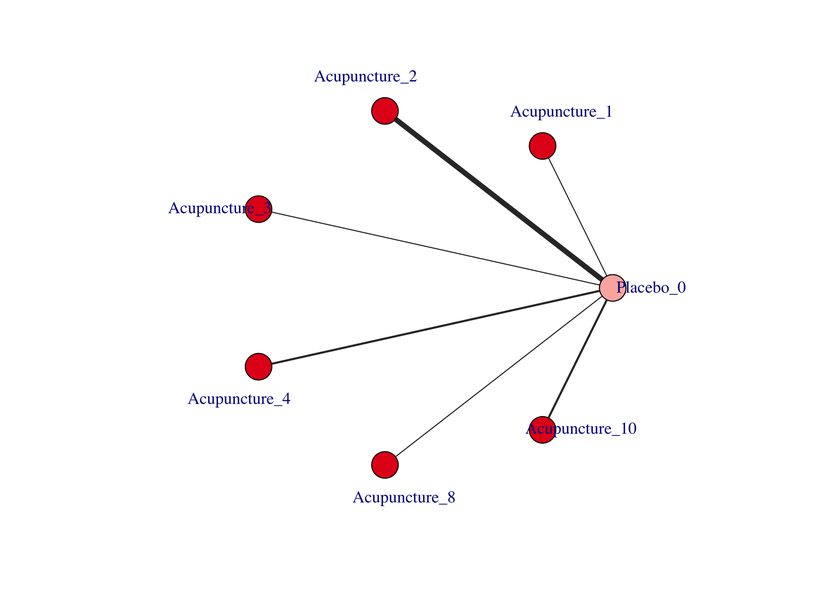


**B. Acupuncture type**


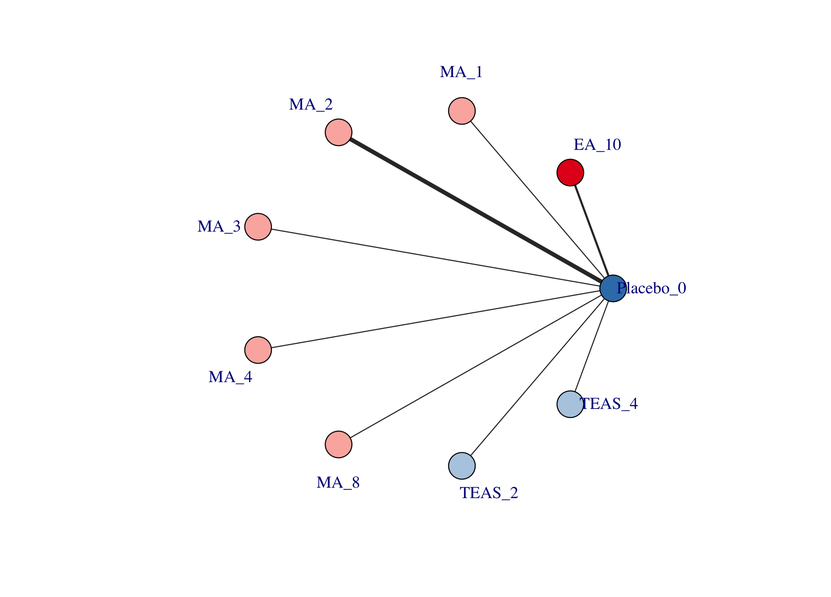


**C. Acupuncture timing nodes**


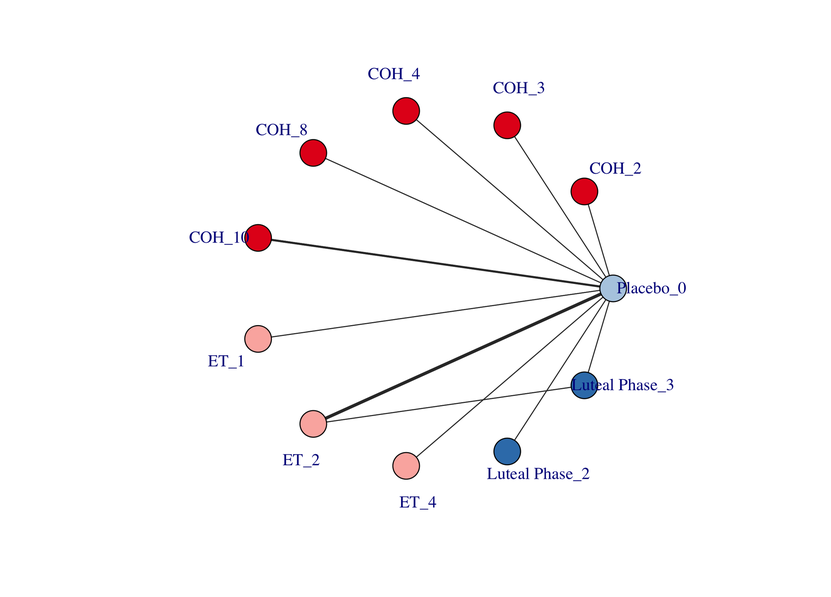


Supplemental Figure 4. Connectivity plots for MR (miscarriage rate). A: overall acupuncture; B: acupuncture type; C: acupuncture timing nodes.

**A. Overall acupuncture**


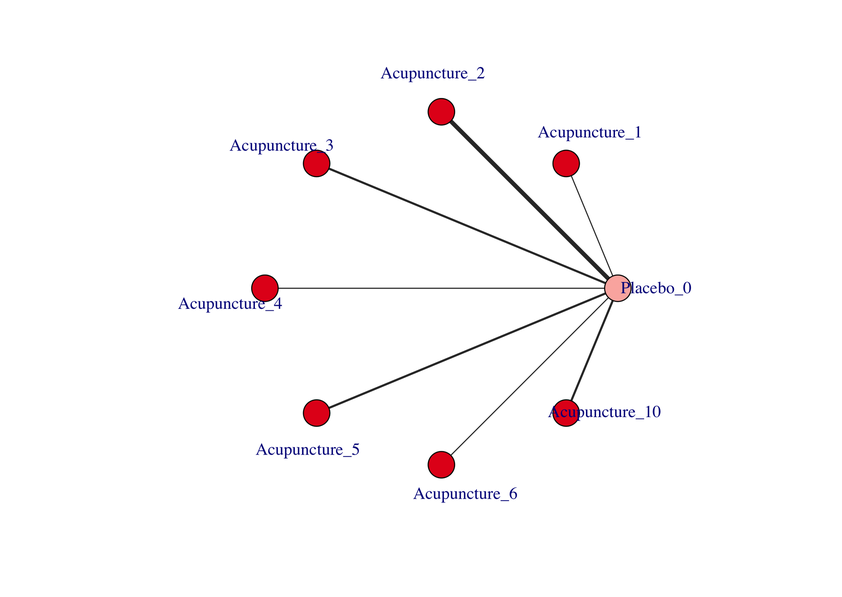


**B. Acupuncture type**


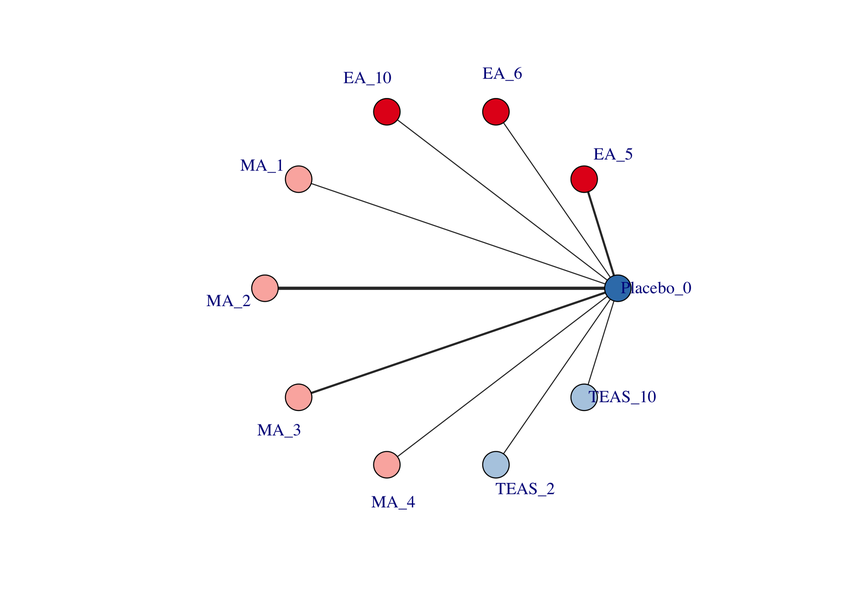


**C. Acupuncture timing nodes**


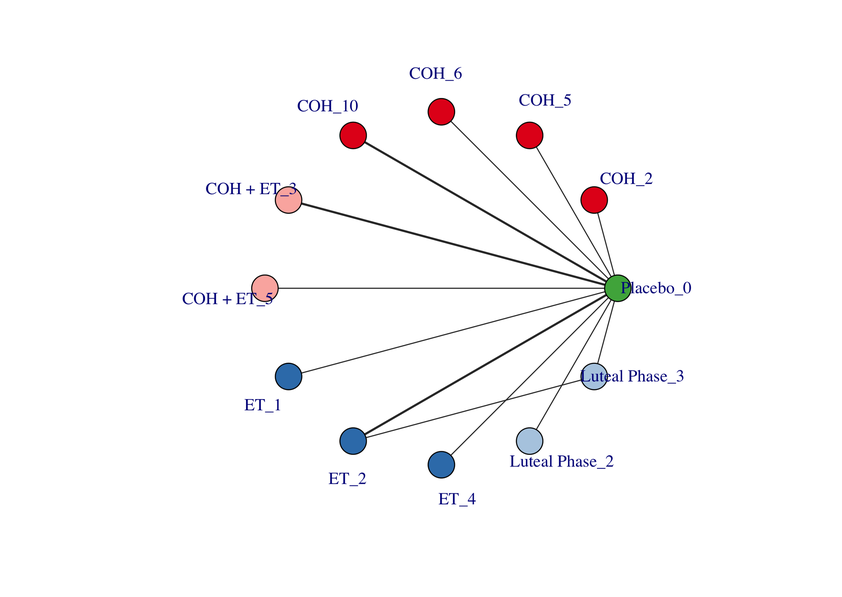


**Consistency**

We assessed consistency by comparing the fit of the consistency model with the unrelated mean effects (UME) model. Similar values for the number of estimated parameters (pD), residual deviance, deviance information criterion (DIC), and between-study standard deviation (SD) indicate that the consistency model provides an adequate representation of the observed evidence structure. The fit indices for all outcomes and analysis levels are summarized in Supplemental Table 8.

Supplemental Table 8. Consistent and UME models fit comparison across outcomes and analysis levels.

| **Outcome** | **Analysis level** | **Model** | **pD** | **Residual deviance** | **DIC** | **SD** | **ΔDIC (UME - Consistent)** | **Δ residual deviance (UME - Consistent)** | **Interpretation** |
| --- | --- | --- | --- | --- | --- | --- | --- | --- | --- |
| CPR (clinical pregnancy rate) | Overall acupuncture | Consistent | 75.04 | 74.6 | 475.36 | 0.32 |  |  |  |
| CPR (clinical pregnancy rate) | Overall acupuncture | UME | 70.47 | 74.17 | 470.35 | 0.24 | -5.01 | -0.43 | Potential residual inconsistency |
| CPR (clinical pregnancy rate) | Acupuncture type | Consistent | 74.26 | 74.7 | 474.66 | 0.45 |  |  |  |
| CPR (clinical pregnancy rate) | Acupuncture type | UME | 68.47 | 73.77 | 467.96 | 0.27 | -6.70 | -0.93 | Potential residual inconsistency |
| CPR (clinical pregnancy rate) | Acupuncture timing nodes | Consistent | 75.39 | 75.04 | 476.14 | 0.32 |  |  |  |
| CPR (clinical pregnancy rate) | Acupuncture timing nodes | UME | 73.7 | 73.89 | 473.31 | 0.27 | -2.83 | -1.15 | Mild UME advantage |
| LBR (live birth rate) | Overall acupuncture | Consistent | 35.34 | 35.65 | 234.52 | 0.38 |  |  |  |
| LBR (live birth rate) | Overall acupuncture | UME | 35.01 | 35.27 | 233.81 | 0.42 | -0.71 | -0.38 | Mild UME advantage |
| LBR (live birth rate) | Acupuncture type | Consistent | 34.81 | 36.07 | 234.42 | 0.4 |  |  |  |
| LBR (live birth rate) | Acupuncture type | UME | 37.29 | 36.53 | 237.35 | 0.44 | 2.93 | 0.46 | No material UME advantage |
| LBR (live birth rate) | Acupuncture timing nodes | Consistent | 38.05 | 37.24 | 238.83 | 0.45 |  |  |  |
| LBR (live birth rate) | Acupuncture timing nodes | UME | 38.02 | 38.21 | 239.76 | 0.33 | 0.93 | 0.97 | No material UME advantage |
| OPR (ongoing pregnancy rate) | Overall acupuncture | Consistent | 28.48 | 28.16 | 184.87 | 0.54 |  |  |  |
| OPR (ongoing pregnancy rate) | Overall acupuncture | UME | 24.95 | 26.08 | 179.26 | 0.46 | -5.61 | -2.08 | Potential residual inconsistency |
| OPR (ongoing pregnancy rate) | Acupuncture type | Consistent | 28.92 | 28.61 | 185.75 | 0.68 |  |  |  |
| OPR (ongoing pregnancy rate) | Acupuncture type | UME | 25.99 | 26.89 | 181.11 | 0.48 | -4.64 | -1.72 | Mild UME advantage |
| OPR (ongoing pregnancy rate) | Acupuncture timing nodes | Consistent | 27.14 | 28.7 | 184.07 | 0.36 |  |  |  |
| OPR (ongoing pregnancy rate) | Acupuncture timing nodes | UME | 28.61 | 28.19 | 185.03 | 0.49 | 0.96 | -0.51 | No material UME advantage |
| BPR (biochemical pregnancy rate) | Overall acupuncture | Consistent | 32.31 | 32.64 | 219.09 | 0.54 |  |  |  |
| BPR (biochemical pregnancy rate) | Overall acupuncture | UME | 32.88 | 32.48 | 219.51 | 0.44 | 0.42 | -0.16 | No material UME advantage |
| BPR (biochemical pregnancy rate) | Acupuncture type | Consistent | 33.09 | 32.32 | 219.55 | 0.82 |  |  |  |
| BPR (biochemical pregnancy rate) | Acupuncture type | UME | 33.16 | 32.05 | 219.35 | 0.49 | -0.20 | -0.27 | Mild UME advantage |
| BPR (biochemical pregnancy rate) | Acupuncture timing nodes | Consistent | 32.93 | 32.27 | 219.34 | 0.42 |  |  |  |
| BPR (biochemical pregnancy rate) | Acupuncture timing nodes | UME | 36.02 | 32.99 | 223.16 | 0.44 | 3.82 | 0.72 | No material UME advantage |
| IR (implantation rate) | Overall acupuncture | Consistent | 21.66 | 25.08 | 165.81 | 0.23 |  |  |  |
| IR (implantation rate) | Overall acupuncture | UME | 22.4 | 25.82 | 167.28 | 0.26 | 1.47 | 0.74 | No material UME advantage |
| IR (implantation rate) | Acupuncture type | Consistent | 24.02 | 25.23 | 168.32 | 0.48 |  |  |  |
| IR (implantation rate) | Acupuncture type | UME | 21.85 | 24.88 | 165.8 | 0.23 | -2.52 | -0.35 | Mild UME advantage |
| IR (implantation rate) | Acupuncture timing nodes | Consistent | 26.06 | 26.07 | 175.75 | 0.79 |  |  |  |
| IR (implantation rate) | Acupuncture timing nodes | UME | 21.56 | 26.52 | 171.69 | 0.24 | -4.06 | 0.45 | Mild UME advantage |
| MR (miscarriage rate) | Overall acupuncture | Consistent | 24.15 | 25.41 | 134.63 | 0.41 |  |  |  |
| MR (miscarriage rate) | Overall acupuncture | UME | 18.72 | 24.29 | 128.08 | 0.34 | -6.55 | -1.12 | Potential residual inconsistency |
| MR (miscarriage rate) | Acupuncture type | Consistent | 26.11 | 26.71 | 137.9 | 0.78 |  |  |  |
| MR (miscarriage rate) | Acupuncture type | UME | 19.65 | 23.59 | 128.3 | 0.3 | -9.60 | -3.12 | Potential residual inconsistency |
| MR (miscarriage rate) | Acupuncture timing nodes | Consistent | 28.2 | 27.64 | 143.62 | 0.81 |  |  |  |
| MR (miscarriage rate) | Acupuncture timing nodes | UME | 22.99 | 23.72 | 134.49 | 0.39 | -9.13 | -3.92 | Potential residual inconsistency |

Note. pD = number of estimated parameters; DIC = deviance information criterion; SD = between-study standard deviation; UME = unrelated mean effects. Lower DIC indicates better model fit and should be interpreted together with residual deviance, pD, SD, and the node-splitting diagnostics. ΔDIC and Δ residual deviance were calculated as UME minus Consistent; negative values indicate better UME fit. ΔDIC <= -5 was interpreted as a potential residual inconsistency signal, whereas smaller negative values were considered a mild UME advantage and non-negative values indicated no material UME advantage.

**Transitivity**

Transitivity requires that direct and indirect evidence are sufficiently comparable so that mixed treatment contrasts can be interpreted within the same evidence network. We assessed this assumption using the MBNMA node-splitting approach, which separates direct and indirect evidence for specific treatment contrasts. Forest plots and density plots were used to compare these contributions for each outcome and analysis level.

Supplemental Figure 5. Node-splitting analysis forest plots for CPR (clinical pregnancy rate). A: overall acupuncture; B: acupuncture type; C: acupuncture timing nodes.

**A. Overall acupuncture**


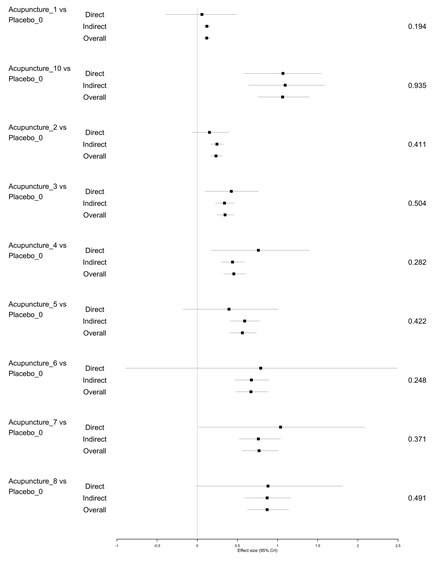


**B. Acupuncture type**


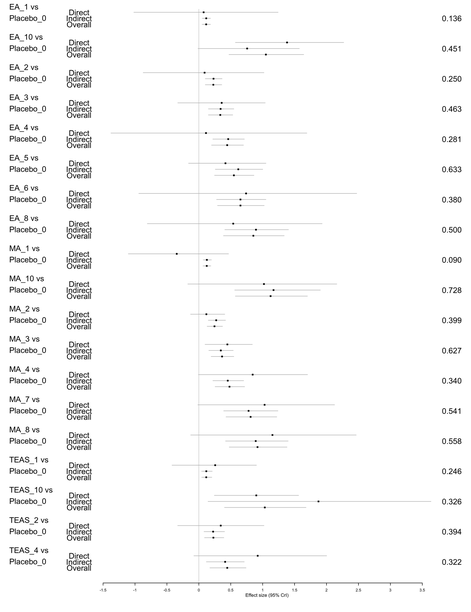


**C. Acupuncture timing nodes**


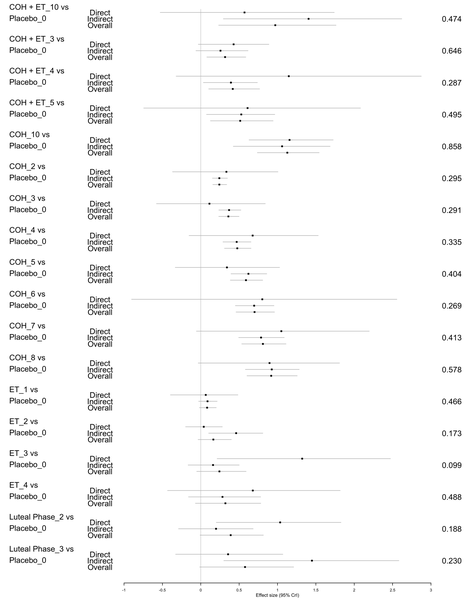


Supplemental Figure 6. Node-splitting analysis density plots for CPR (clinical pregnancy rate). A: overall acupuncture; B: acupuncture type; C: acupuncture timing nodes.

**A. Overall acupuncture**


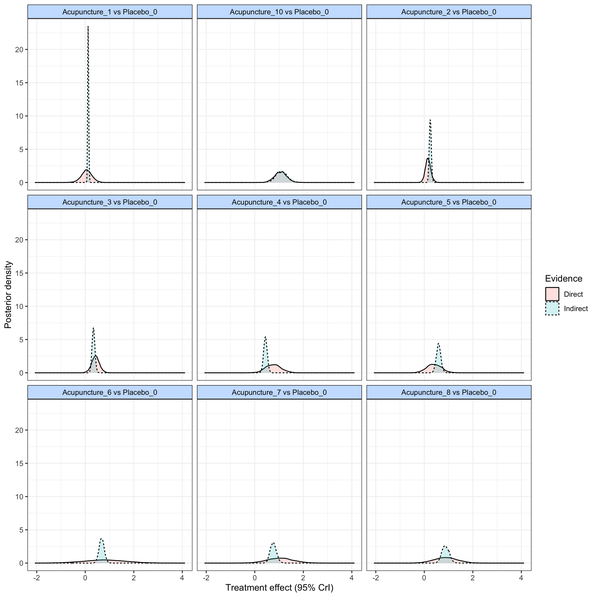


**B. Acupuncture type**


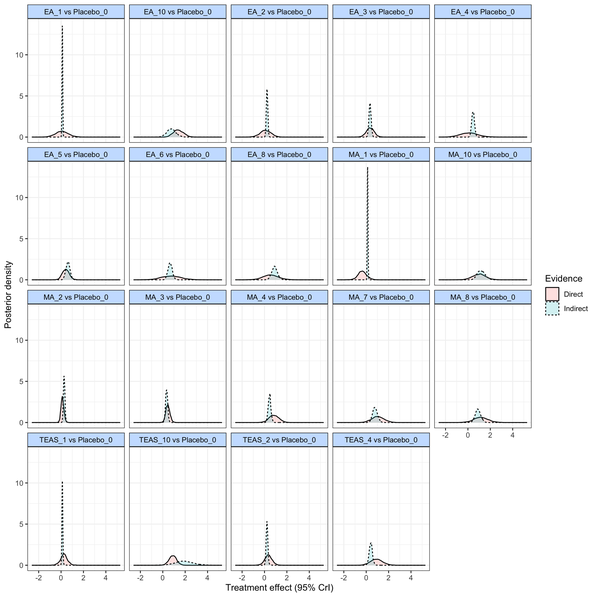


**C. Acupuncture timing nodes**


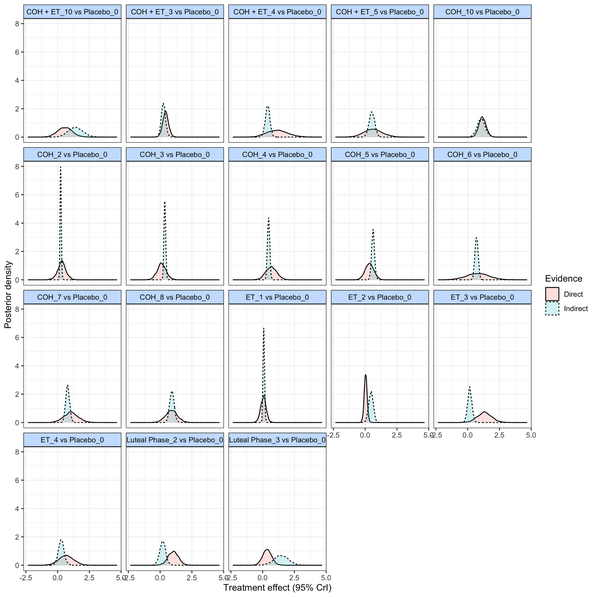


Supplemental Figure 7. Node-splitting analysis forest plots for LBR (live birth rate). A: overall acupuncture; B: acupuncture type; C: acupuncture timing nodes.

**A. Overall acupuncture**


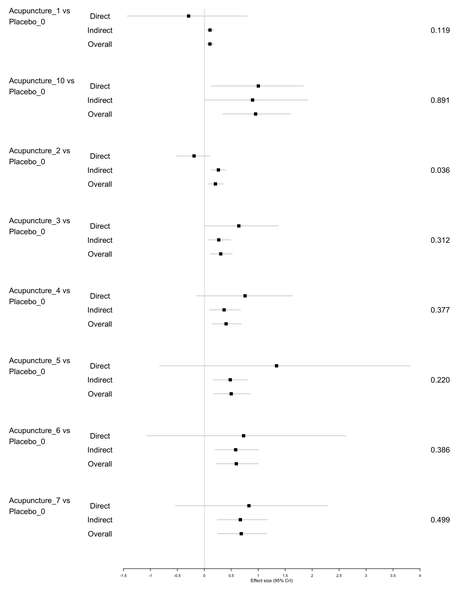


**B. Acupuncture type**


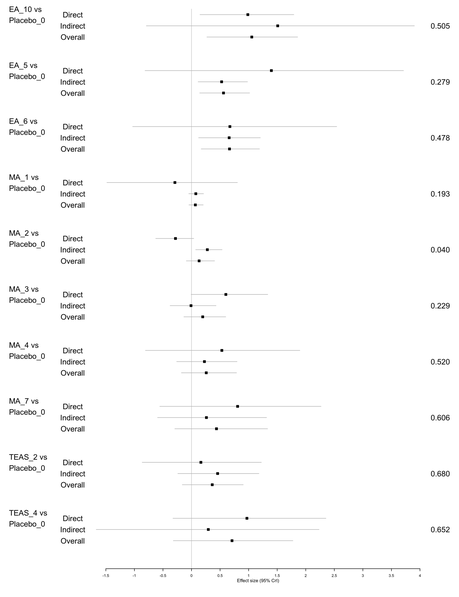


**C. Acupuncture timing nodes**


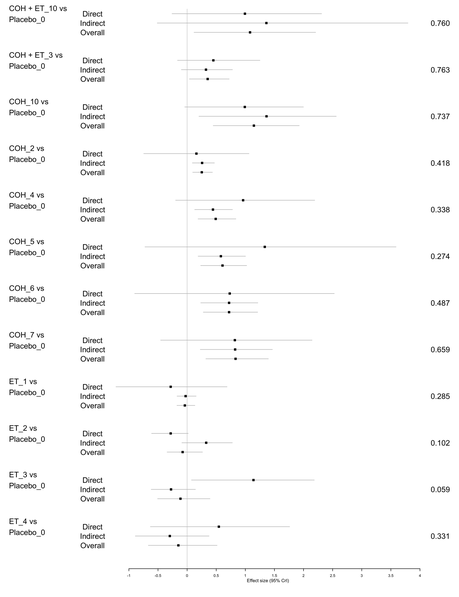


Supplemental Figure 8. Node-splitting analysis density plots for LBR (live birth rate). A: overall acupuncture; B: acupuncture type; C: acupuncture timing nodes.

**A. Overall acupuncture**


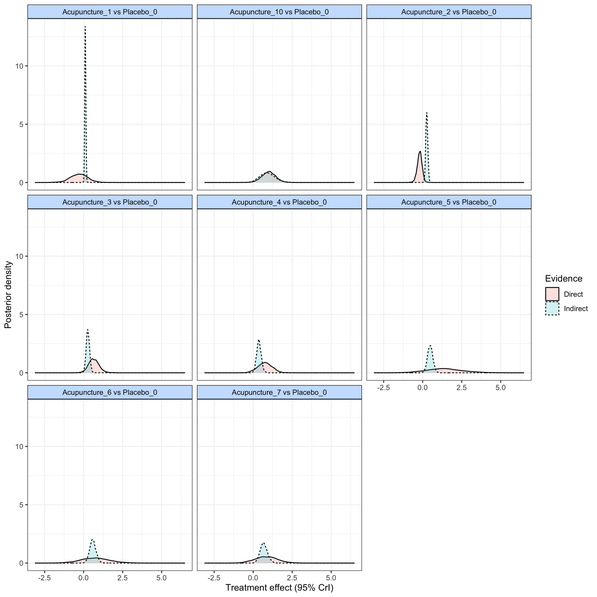


**B. Acupuncture type**


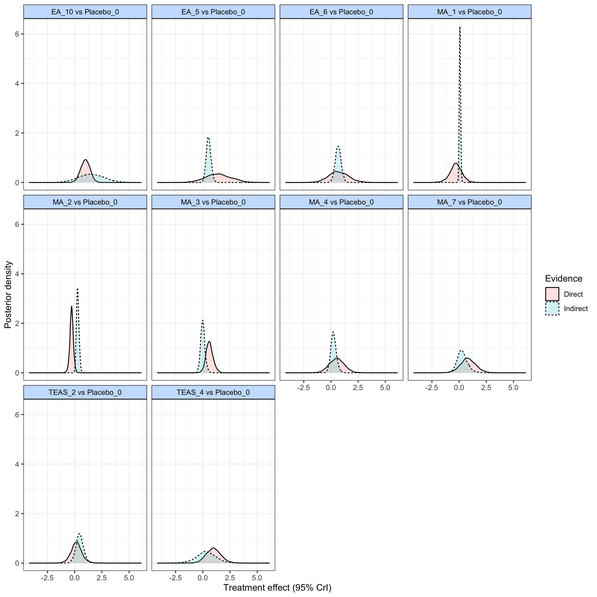


**C. Acupuncture timing nodes**


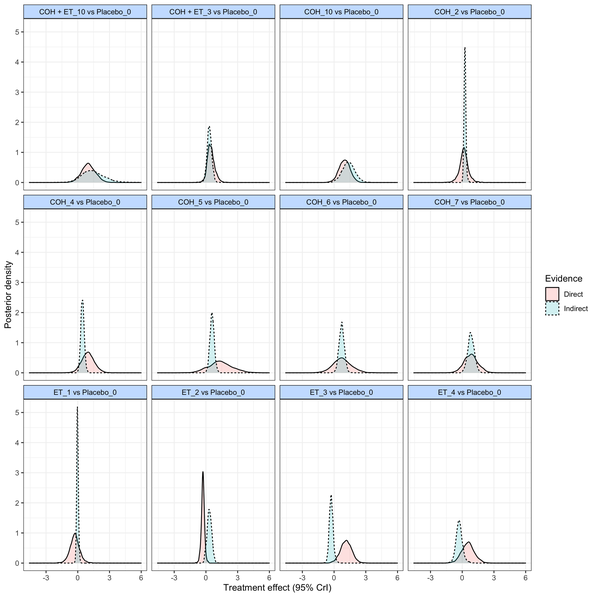


Supplemental Figure 9. Node-splitting analysis forest plots for OPR (ongoing pregnancy rate). A: overall acupuncture; B: acupuncture type; C: acupuncture timing nodes.

**A. Overall acupuncture**


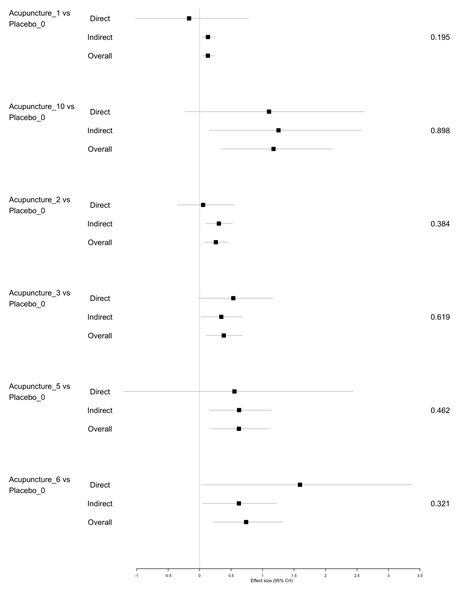


**B. Acupuncture type**


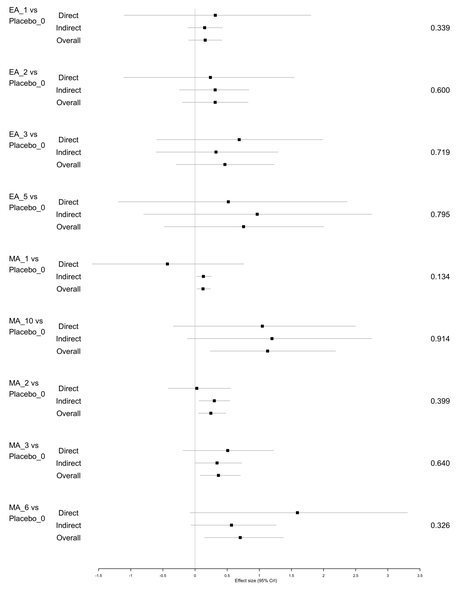


**C. Acupuncture timing nodes**


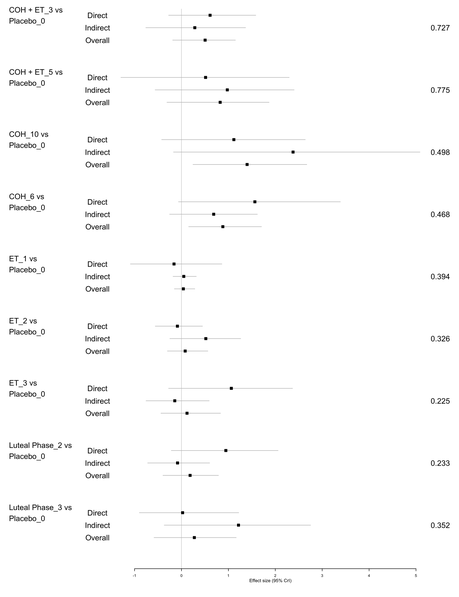


Supplemental Figure 10. Node-splitting analysis density plots for OPR (ongoing pregnancy rate). A: overall acupuncture; B: acupuncture type; C: acupuncture timing nodes.

**A. Overall acupuncture**


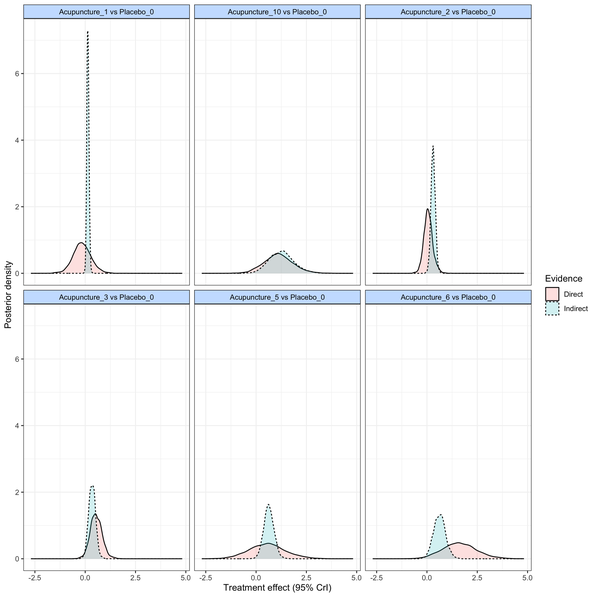


**B. Acupuncture type**


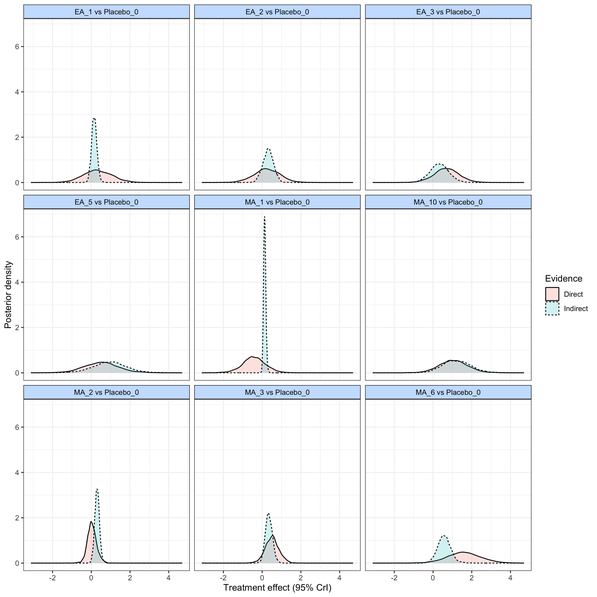


**C. Acupuncture timing nodes**


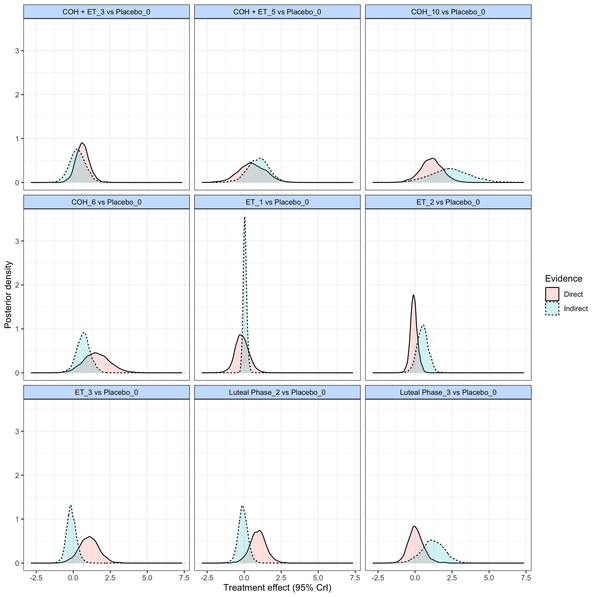


Supplemental Figure 11. Node-splitting analysis forest plots for BPR (biochemical pregnancy rate). A: overall acupuncture; B: acupuncture type; C: acupuncture timing nodes.

**A. Overall acupuncture**


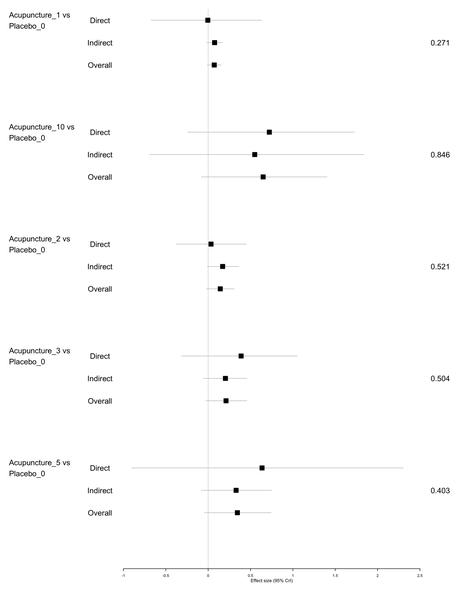


**B. Acupuncture type**


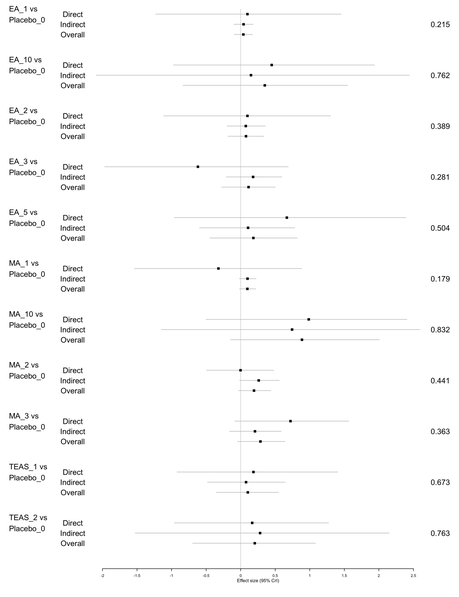


**C. Acupuncture timing nodes**


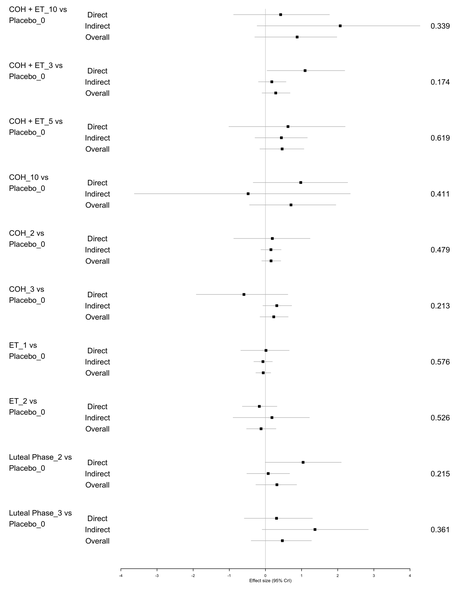


Supplemental Figure 12. Node-splitting analysis density plots for BPR (biochemical pregnancy rate). A: overall acupuncture; B: acupuncture type; C: acupuncture timing nodes.

**A. Overall acupuncture**


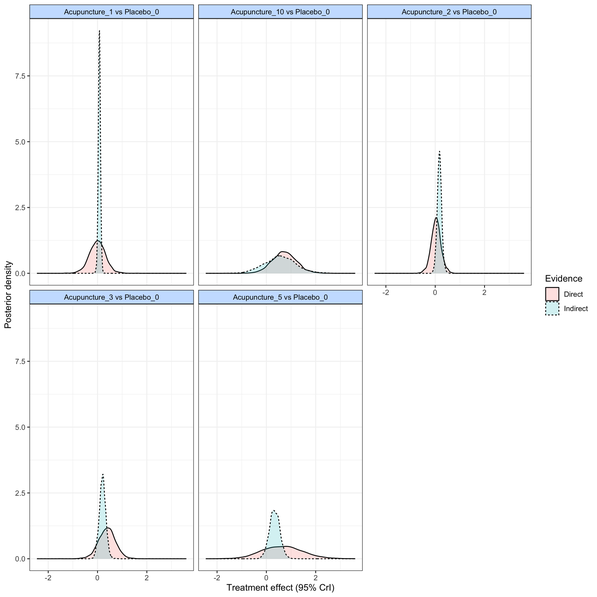


**B. Acupuncture type**


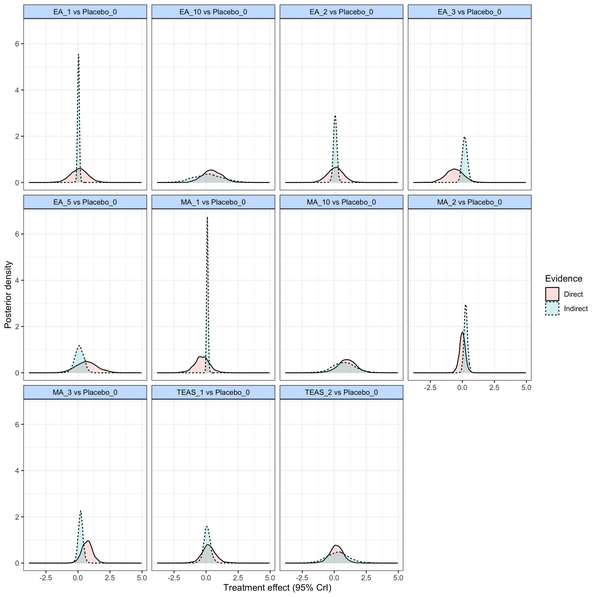


**C. Acupuncture timing nodes**


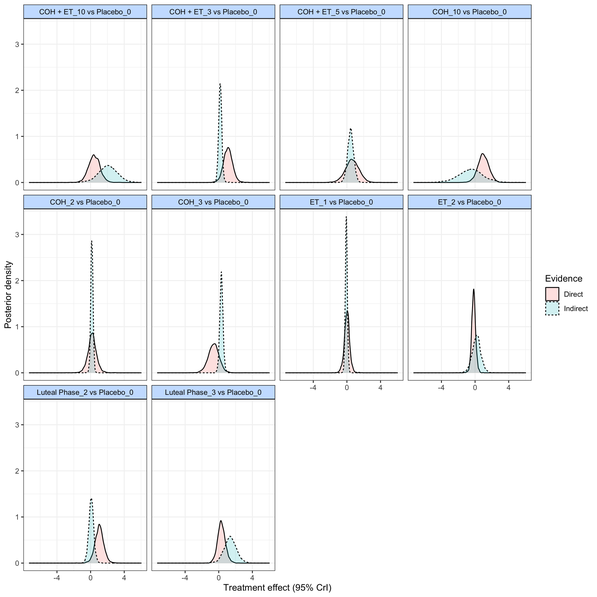


Supplemental Figure 13. Node-splitting analysis forest plots for IR (implantation rate). A: overall acupuncture; B: acupuncture type; C: acupuncture timing nodes.

**A. Overall acupuncture**


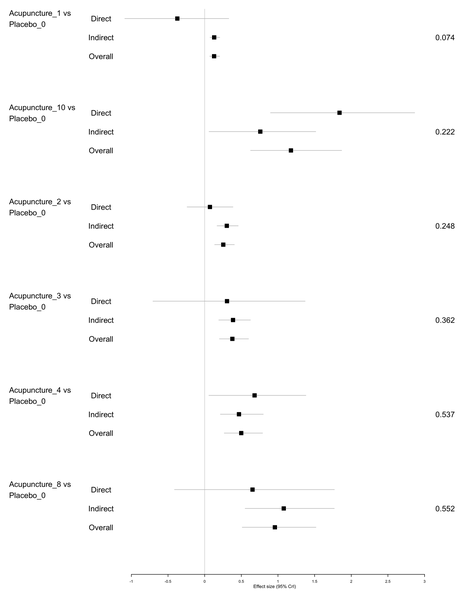


**B. Acupuncture type**


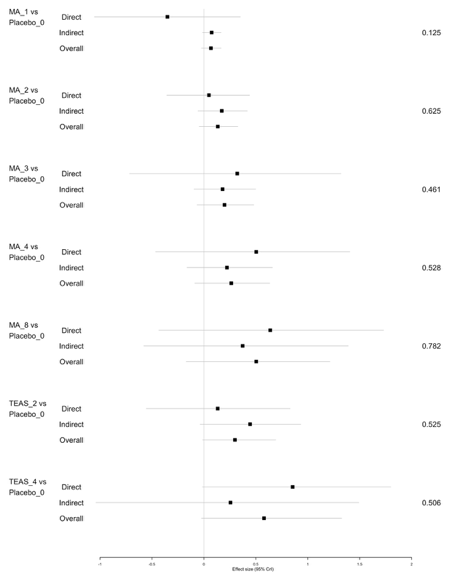


**C. Acupuncture timing nodes**


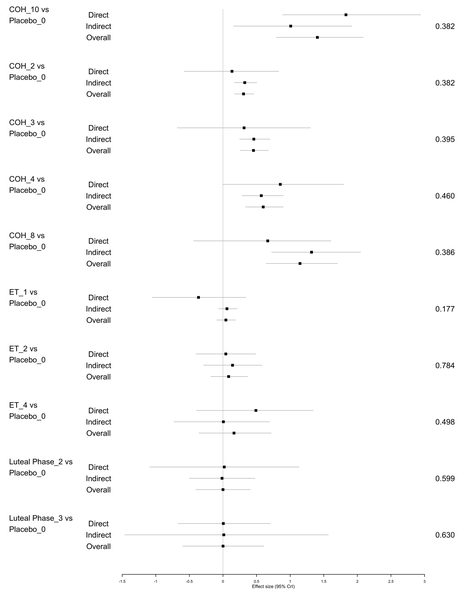


Supplemental Figure 14. Node-splitting analysis density plots for IR (implantation rate). A: overall acupuncture; B: acupuncture type; C: acupuncture timing nodes.

**A. Overall acupuncture**


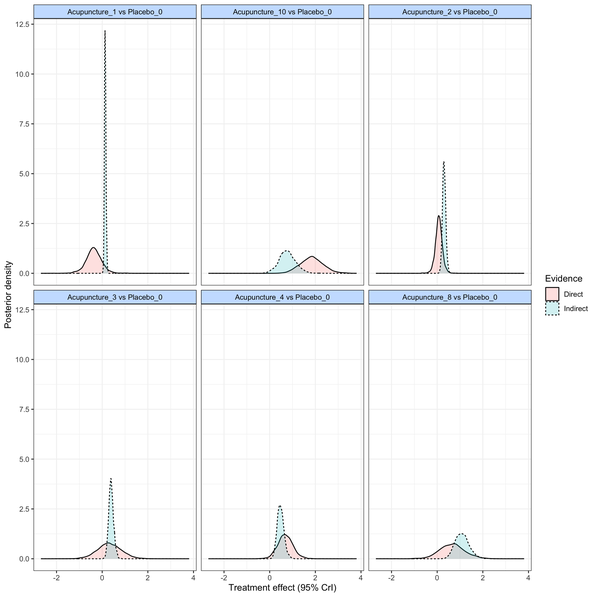


**B. Acupuncture type**


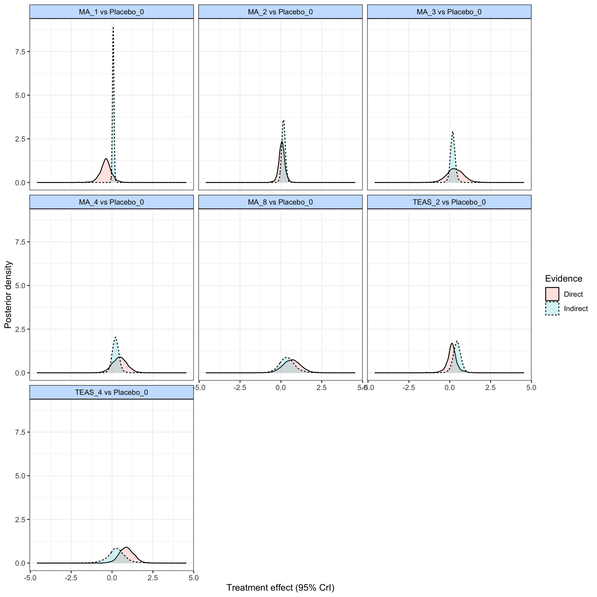


**C. Acupuncture timing nodes**


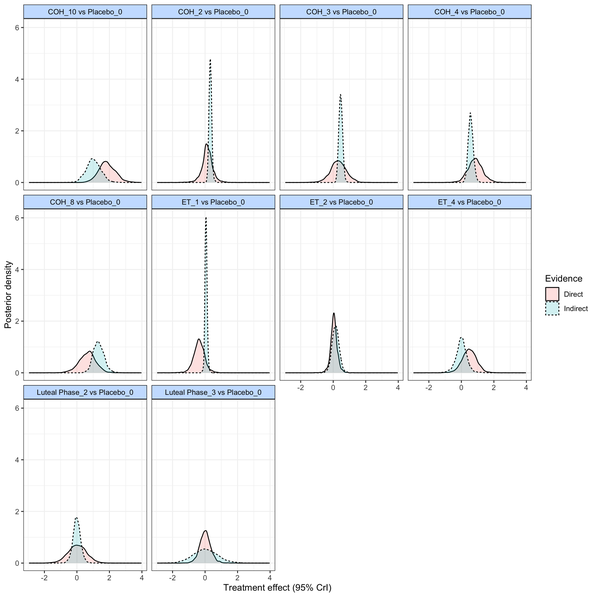


Supplemental Figure 15. Node-splitting analysis forest plots for MR (miscarriage rate). A: overall acupuncture; B: acupuncture type; C: acupuncture timing nodes.

**A. Overall acupuncture**


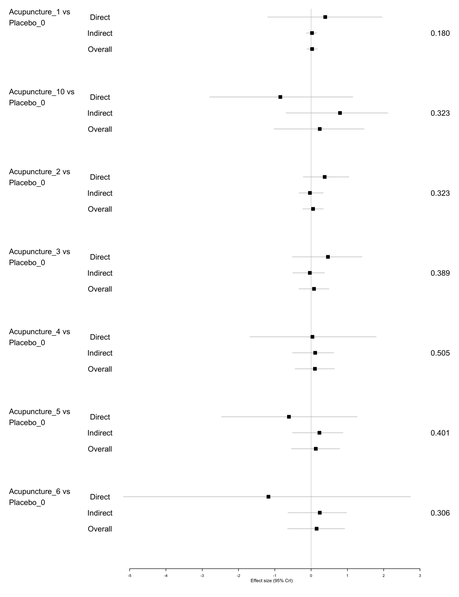


**B. Acupuncture type**


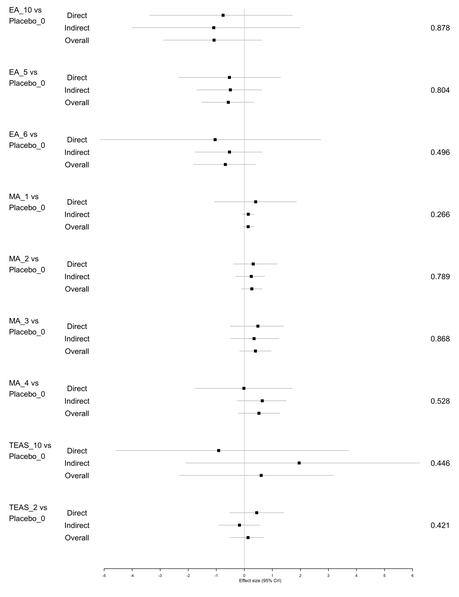


**C. Acupuncture timing nodes**


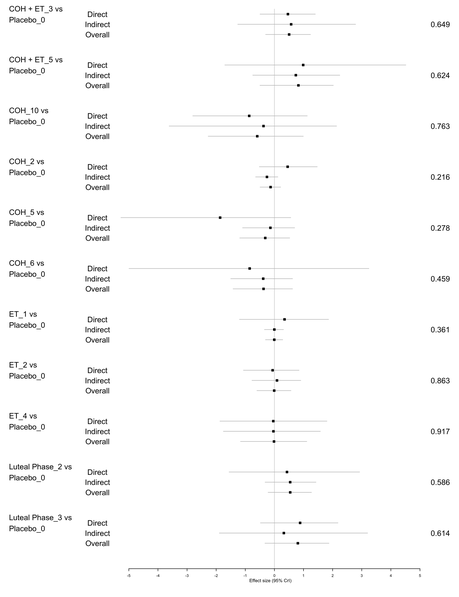


Supplemental Figure 16. Node-splitting analysis density plots for MR (miscarriage rate). A: overall acupuncture; B: acupuncture type; C: acupuncture timing nodes.

**A. Overall acupuncture**


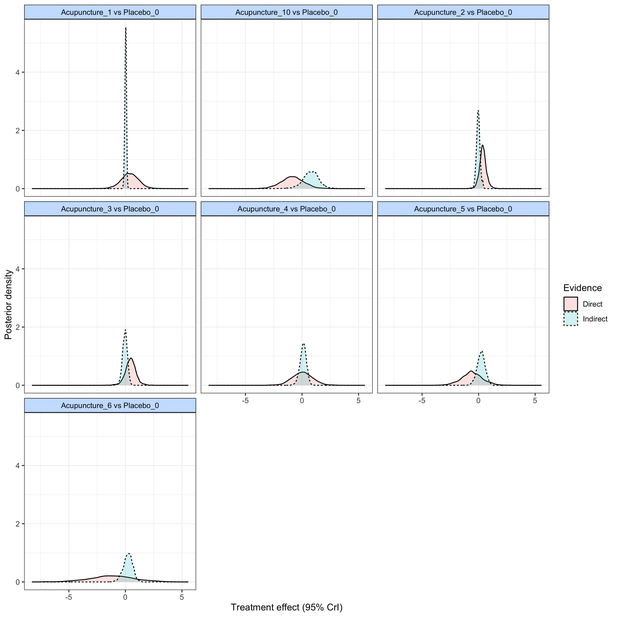


**B. Acupuncture type**


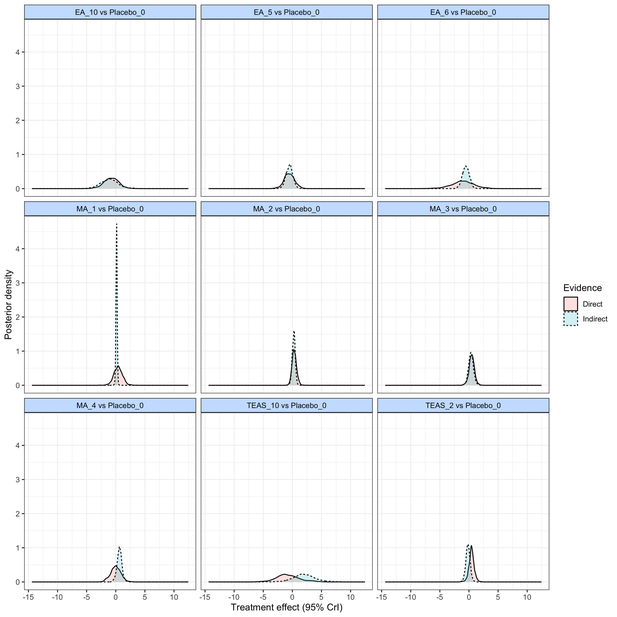


**C. Acupuncture timing nodes**


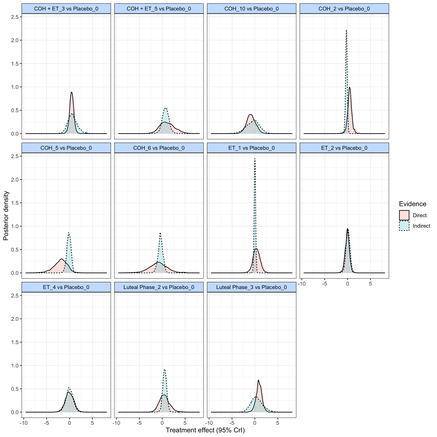


1. **Supplemental File 7. Characteristics of the studies included in the meta-analysis.**

Supplemental Table 9. Characteristics of the studies included in the meta-analysis.

| **First author, year** | **Country** | **Transfer type** | **Intervention group, n** | **Control group, n** | **Total sample size** | **Intervention modality** | **Timing of intervention** | **Sessions/retention time** | **Comparator** | **Blinding** | **Reproductive outcomes reported** |
| --- | --- | --- | --- | --- | --- | --- | --- | --- | --- | --- | --- |
| So et al., 2009 | China | Fresh embryo transfer | 185 | 185 | 370 | Acupuncture | Before and after embryo transfer (before and after ET) | 2 session(s); 25 min retention | Streitberger nonpenetrating sham acupuncture | participants: yes; interventionists: no; outcome assessors: yes | biochemical pregnancy; clinical pregnancy; ongoing pregnancy; live birth; implantation; miscarriage |
| So et al., 2010 | China | Frozen-thawed embryo transfer | 113 | 113 | 226 | Acupuncture | After embryo transfer (after ET) | 1 session(s); 25 min retention | Streitberger nonpenetrating sham acupuncture | participants: yes; interventionists: no; outcome assessors: yes | biochemical pregnancy; clinical pregnancy; ongoing pregnancy; live birth; implantation; miscarriage |
| Andersen et al., 2010 | Denmark | Fresh embryo transfer | 305 | 313 | 618 | Acupuncture | Before and after embryo transfer (before and after ET) | 2 session(s); 30 min retention | Streitberger nonpenetrating sham acupuncture | participants: yes; interventionists: no; outcome assessors: no | biochemical pregnancy; clinical pregnancy; ongoing pregnancy; live birth |
| Dong et al., 2024 | China | Fresh embryo transfer | 34 | 35 | 69 | Acupuncture | Before embryo transfer (from oocyte retrieval to before ET) | 4 session(s); 30 min retention | superficial needling at nonacupoints | participants: yes; interventionists: no; outcome assessors: yes | clinical pregnancy; live birth; implantation; miscarriage |
| Isoyama et al., 2012 | Brazil | Fresh embryo transfer | 22 | 21 | 43 | Acupuncture | Controlled ovarian hyperstimulation and embryo transfer (from oocyte retrieval to before ET and after ET) | 4 session(s); 25 min retention | superficial needling at nonacupoints | participants: yes; interventionists: no; outcome assessors: yes | clinical pregnancy |
| Dieterle et al., 2006 | Germany | Fresh and/or frozen embryo transfer | 116 | 109 | 225 | Acupuncture | Luteal phase (after ET) | 2 session(s); 30 min retention | superficial needling at nonacupoints | participants: yes; interventionists: no; outcome assessors: no | biochemical pregnancy; clinical pregnancy; ongoing pregnancy; implantation; miscarriage |
| Smith et al., 2018 | Australia | Fresh embryo transfer | 405 | 404 | 809 | Acupuncture | Controlled ovarian hyperstimulation and embryo transfer (from oocyte retrieval to before ET and after ET) | 3 session(s); 25 min retention | Streitberger nonpenetrating sham acupuncture | participants: yes; interventionists: no; outcome assessors: yes | clinical pregnancy; live birth |
| Xiang et al., 2021 | China | Fresh and/or frozen embryo transfer | 38 | 38 | 76 | Electroacupuncture | Controlled ovarian hyperstimulation (before oocyte retrieval) | NR session(s); 25 min retention | nonpenetrating sham acupuncture | participants: yes; interventionists: no; outcome assessors: no | clinical pregnancy; live birth |
| Wu et al., 2019 | China | Fresh embryo transfer | 33 | 33 | 66 | Electroacupuncture | Controlled ovarian hyperstimulation (before ET) | NR session(s); 25 min retention | nonpenetrating sham acupuncture | participants: yes; interventionists: no; outcome assessors: no | clinical pregnancy |
| Zheng et al., 2015 | China | Fresh embryo transfer | 56 | 56 | 112 | TEAS, 20 mA | Controlled ovarian hyperstimulation (before oocyte retrieval) | NR session(s); 30 min retention | mock TEAS | participants: yes; interventionists: no; outcome assessors: no | clinical pregnancy; miscarriage |
| Zhai et al., 2022 | China | Fresh and/or frozen embryo transfer | 160 | 40 | 200 | TEAS, 20 mA; TEAS, 30 mA; TEAS, 40 mA; TEAS, 50 mA | Controlled ovarian hyperstimulation (before oocyte retrieval) | 10 session(s); 30 min retention | mock TEAS | participants: yes; interventionists: no; outcome assessors: no | clinical pregnancy |
| Pang et al., 2025 | China | Fresh embryo transfer | 45 | 45 | 90 | Electroacupuncture | Controlled ovarian hyperstimulation (before oocyte retrieval) | 6 session(s); 25 min retention | nonpenetrating sham acupuncture | participants: yes; interventionists: no; outcome assessors: yes | clinical pregnancy; live birth; miscarriage |
| Smith et al., 2006 | Australia | Fresh embryo transfer | 110 | 118 | 228 | Acupuncture | Controlled ovarian hyperstimulation and embryo transfer (from oocyte retrieval to before ET and after ET) | 3 session(s); 25 min retention | Streitberger nonpenetrating sham acupuncture | participants: yes; interventionists: no; outcome assessors: no | clinical pregnancy; ongoing pregnancy |
| Moy et al., 2011 | United States | Fresh embryo transfer | 86 | 74 | 160 | Acupuncture | Before and after embryo transfer (before and after ET) | 2 session(s); 25 min retention | superficial needling at nonacupoints | participants: yes; interventionists: no; outcome assessors: yes | biochemical pregnancy; clinical pregnancy |
| Xia et al., 2023 | China | Fresh and/or frozen embryo transfer | 30 | 30 | 60 | Acupuncture | Controlled ovarian hyperstimulation (before oocyte retrieval) | NR session(s); 30 min retention | Streitberger nonpenetrating sham acupuncture | participants: yes; interventionists: no; outcome assessors: no | ongoing pregnancy |
| Shuai et al., 2019 | China | Fresh embryo transfer | 61 | 61 | 122 | TEAS, 20 mA | Controlled ovarian hyperstimulation (from oocyte retrieval to before ET) | NR session(s); 30 min retention | mock TEAS | participants: yes; interventionists: no; outcome assessors: no | clinical pregnancy; live birth; implantation |
| Altutunji et al., 2019 | China | Fresh embryo transfer | 33 | 69 | 102 | Acupuncture | Controlled ovarian hyperstimulation (before oocyte retrieval) | NR session(s); 30 min retention | blank/wait-list or usual care control | participants: no; interventionists: no; outcome assessors: no | biochemical pregnancy; clinical pregnancy; ongoing pregnancy; miscarriage |
| Ho et al., 2009 | China | Fresh embryo transfer | 30 | 14 | 44 | Electroacupuncture | Controlled ovarian hyperstimulation (before oocyte retrieval) | 4 session(s); 30 min retention | blank/wait-list or usual care control | participants: no; interventionists: no; outcome assessors: no | clinical pregnancy |
| Peyvandi et al., 2016 | Iran | Fresh and/or frozen embryo transfer | 82 | 82 | 164 | Electroacupuncture | Controlled ovarian hyperstimulation and embryo transfer (before and after oocyte retrieval, and before ET) | 3 session(s); 25 min retention | blank/wait-list or usual care control | participants: no; interventionists: no; outcome assessors: yes | clinical pregnancy; ongoing pregnancy |
| Seto et al., 2017 | China | Fresh and/or frozen embryo transfer | 93 | 119 | 212 | Acupuncture | Before and after embryo transfer (before and after ET) | 2 session(s); 25 min retention | Streitberger nonpenetrating sham acupuncture | participants: yes; interventionists: no; outcome assessors: yes | ongoing pregnancy; live birth |
| Gillerman et al., 2018 | United Kingdom | Fresh and/or frozen embryo transfer | 64 | 69 | 133 | Acupuncture | Controlled ovarian hyperstimulation and embryo transfer (after ovarian stimulation, before ET, and after ET) | 3 session(s); 25 min retention | blank/wait-list or usual care control | participants: no; interventionists: no; outcome assessors: yes | biochemical pregnancy; live birth; miscarriage |
| Paulus et al., 2002 | Germany | Fresh embryo transfer | 80 | 80 | 160 | Acupuncture | Before and after embryo transfer (before and after ET) | 2 session(s); 25 min retention | blank/wait-list or usual care control | participants: no; interventionists: no; outcome assessors: yes | clinical pregnancy |
| Westergaard et al., 2006 | Denmark | Fresh embryo transfer | 186 | 87 | 273 | Acupuncture | Before and after embryo transfer; Luteal phase (before and after ET; before and after ET, with an additional session 2 days later) | 2; 3 session(s); 25 min retention | blank/wait-list or usual care control | participants: no; interventionists: no; outcome assessors: yes | biochemical pregnancy; clinical pregnancy; ongoing pregnancy; implantation; miscarriage |
| Magarelli et al., 2009 | United States | Fresh and/or frozen embryo transfer | 34 | 33 | 67 | Electroacupuncture | Controlled ovarian hyperstimulation and embryo transfer (before oocyte retrieval, before ET, and after ET) | 10 session(s); 25 min retention | blank/wait-list or usual care control | participants: no; interventionists: no; outcome assessors: yes | biochemical pregnancy; clinical pregnancy; live birth; miscarriage |
| Morin et al., 2017 | United States | Fresh and/or frozen embryo transfer | 200 | 203 | 403 | Acupuncture | Before and after embryo transfer (before and after ET) | 2 session(s); 25 min retention | blank/wait-list or usual care control | participants: no; interventionists: no; outcome assessors: yes | clinical pregnancy; live birth; implantation |
| Jun et al., 2009 | China | Fresh embryo transfer | 30 | 30 | 60 | Electroacupuncture | Controlled ovarian hyperstimulation (before and during ovarian stimulation) | NR session(s); 30 min retention | blank/wait-list or usual care control | participants: no; interventionists: no; outcome assessors: no | clinical pregnancy; live birth; implantation; miscarriage |
| Qian et al., 2015 | China | Fresh embryo transfer | 57 | 57 | 114 | Acupuncture | Controlled ovarian hyperstimulation (from ovarian stimulation to before ET) | 3 session(s); 30 min retention | blank/wait-list or usual care control | participants: no; interventionists: no; outcome assessors: yes | clinical pregnancy; implantation |
| Cui et al., 2011 | China | Fresh embryo transfer | 34 | 32 | 66 | Electroacupuncture | Controlled ovarian hyperstimulation (before and during ovarian stimulation) | 5 session(s); 30 min retention | blank/wait-list or usual care control | participants: no; interventionists: no; outcome assessors: yes | clinical pregnancy; live birth; miscarriage |
| Dehghani et al., 2020 | Iran | Fresh embryo transfer | 124 | 62 | 186 | Electroacupuncture | Before embryo transfer; Before and after embryo transfer (before ET; before and after ET) | 1; 2 session(s); 25 min retention | blank/wait-list or usual care control | participants: no; interventionists: no; outcome assessors: yes | biochemical pregnancy; clinical pregnancy; ongoing pregnancy |
| Domar et al., 2009 | United States | Fresh embryo transfer | 68 | 78 | 146 | Acupuncture | Before and after embryo transfer (before and after ET) | 2 session(s); 25 min retention | blank/wait-list or usual care control | participants: no; interventionists: no; outcome assessors: no | clinical pregnancy |
| Feng et al., 2022 | China | Fresh and/or frozen embryo transfer | 362 | 369 | 731 | TEAS, 20 mA | Controlled ovarian hyperstimulation (before and after ET) | 2 session(s); 30 min retention | blank/wait-list or usual care control | participants: no; interventionists: no; outcome assessors: no | biochemical pregnancy; clinical pregnancy; live birth; implantation; miscarriage |
| Guven et al., 2020 | Turkey | Fresh embryo transfer | 38 | 38 | 76 | Acupuncture | Before and after embryo transfer (1 week before ET, before ET, and after ET) | 3 session(s); 30 min retention | blank/wait-list or usual care control | participants: no; interventionists: no; outcome assessors: yes | clinical pregnancy; ongoing pregnancy; live birth |
| Li et al., 2014 | China | Fresh embryo transfer | 102 | 98 | 200 | Electroacupuncture | Controlled ovarian hyperstimulation (from ovarian stimulation to before ET) | 5 session(s); 30 min retention | blank/wait-list or usual care control | participants: no; interventionists: no; outcome assessors: yes | clinical pregnancy |
| Rashidi et al., 2013 | Iran | Fresh embryo transfer | 31 | 31 | 62 | Electroacupuncture | Controlled ovarian hyperstimulation and embryo transfer (during pituitary downregulation, before ovulation, before oocyte retrieval, before ET, and after ET) | 5 session(s); 30 min retention | blank/wait-list or usual care control | participants: no; interventionists: no; outcome assessors: yes | biochemical pregnancy; clinical pregnancy; ongoing pregnancy; miscarriage |
| Shen et al., 2022 | China | Frozen-thawed embryo transfer | 30 | 30 | 60 | Electroacupuncture | Controlled ovarian hyperstimulation (COH) | NR session(s); 30 min retention | blank/wait-list or usual care control | participants: no; interventionists: no; outcome assessors: no | clinical pregnancy; implantation; miscarriage |
| Xu et al., 2022 | China | Frozen-thawed embryo transfer | 30 | 30 | 60 | Acupuncture | Controlled ovarian hyperstimulation (COH) | NR session(s); 30 min retention | blank/wait-list or usual care control | participants: no; interventionists: no; outcome assessors: yes | clinical pregnancy; implantation |
| Zhong et al., 2023 | China | Fresh and/or frozen embryo transfer | 48 | 48 | 96 | Electroacupuncture | Controlled ovarian hyperstimulation (COH) | 10 session(s); 25 min retention | blank/wait-list or usual care control | participants: no; interventionists: no; outcome assessors: yes | biochemical pregnancy; clinical pregnancy |
| Zhong et al., 2017 | China | Fresh and/or frozen embryo transfer | 735 | 1026 | 1761 | TEAS, 20 mA | Before embryo transfer (before ET) | 1 session(s); 30 min retention | blank/wait-list or usual care control | participants: no; interventionists: no; outcome assessors: no | biochemical pregnancy; clinical pregnancy |
| Zhou et al., 2016 | China | Fresh embryo transfer | 30 | 33 | 63 | Electroacupuncture | Controlled ovarian hyperstimulation (COH) | 10 session(s); 30 min retention | blank/wait-list or usual care control | participants: no; interventionists: no; outcome assessors: no | clinical pregnancy; implantation |
| Craig et al., 2014 | United States | Fresh and/or frozen embryo transfer | 57 | 56 | 113 | Acupuncture | Before and after embryo transfer (before and after ET) | 2 session(s); 25 min retention | blank/wait-list or usual care control | participants: no; interventionists: no; outcome assessors: yes | biochemical pregnancy; clinical pregnancy; live birth |
| Wu et al., 2022 | China | Fresh embryo transfer | 40 | 43 | 83 | Acupuncture | Controlled ovarian hyperstimulation (COH) | 10 session(s); 25 min retention | blank/wait-list or usual care control | participants: no; interventionists: no; outcome assessors: yes | clinical pregnancy; live birth |
| Qu et al., 2017 | China | Fresh embryo transfer | 372 | 109 | 481 | TEAS | Embryo transfer period | 2 session(s); 30 min retention | blank/wait-list or usual care control | participants: no; interventionists: no; outcome assessors: yes | clinical pregnancy; live birth; implantation |

1. **Supplemental File 8. Risk of bias assessment.**

Supplemental Figure 17. RoB 2 traffic-light plot for included studies.


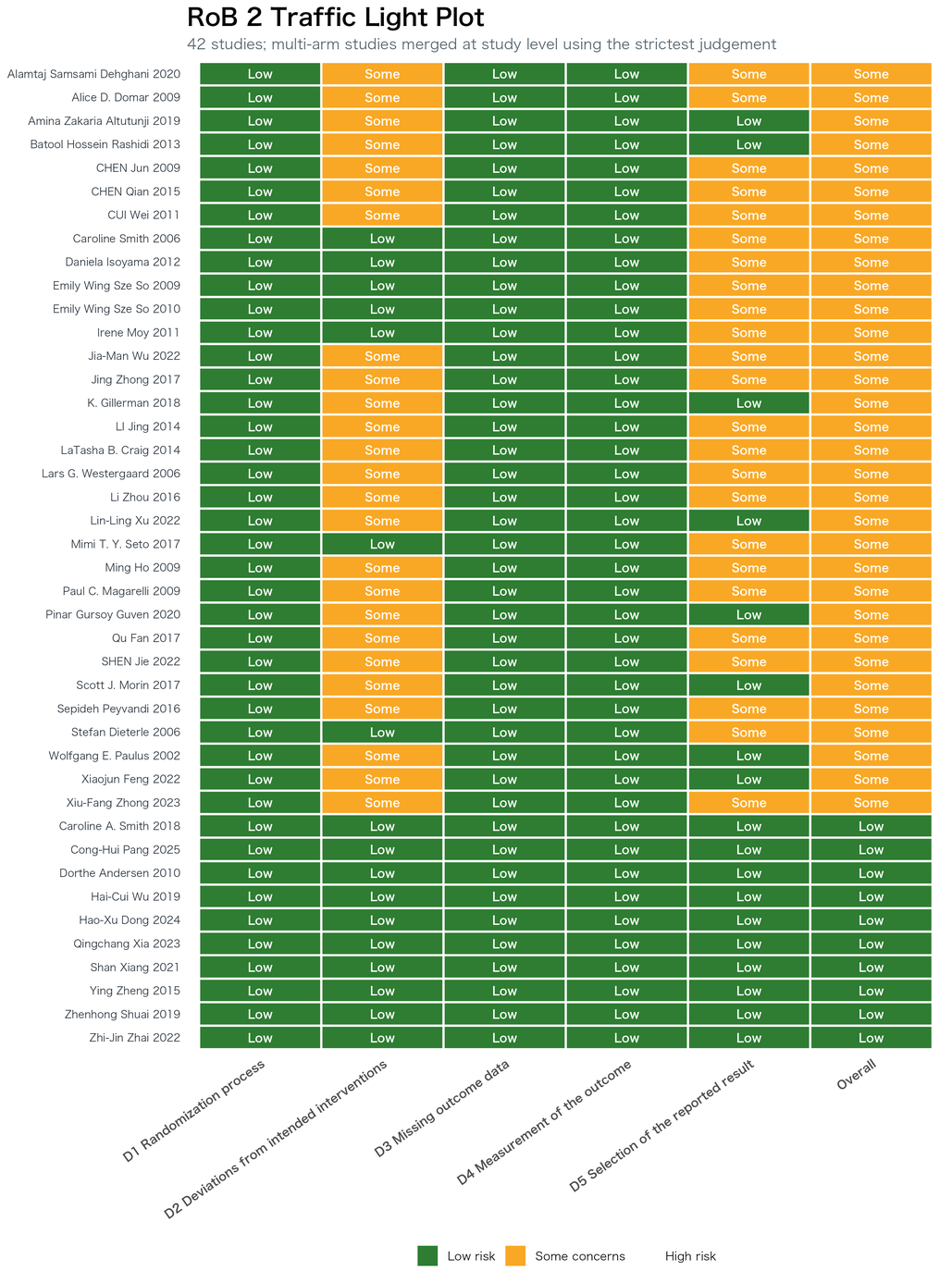


1. **Supplemental File 9. GRADE summary of findings.**

Supplemental Table 10. Summary of findings for overall and subgroup analyses of acupuncture-assisted reproduction outcomes.

| **Assessment group** | **Outcome** | **Code** | **Subgroup** | **Level** | **Studies** | **Participants acupuncture** | **Participants control** | **Control risk** | **RR (95% CI)** | **I2** | **Absolute difference per 1,000** | **Risk of bias** | **Inconsistency** | **Indirectness** | **Imprecision** | **Publication bias** | **Total downgrade** | **GRADE** |
| --- | --- | --- | --- | --- | --- | --- | --- | --- | --- | --- | --- | --- | --- | --- | --- | --- | --- | --- |
| Overall | Clinical pregnancy rate | CPR | Overall | Overall | 46 | 4513 | 4781 | 36.9% | 1.25 (1.14 to 1.37) | 53% | 92 more per 1,000 (52 more per 1,000 to 136 more per 1,000) | -1 | -1 | 0 | 0 | -1 | 3 | Very low |
| Overall | Live birth rate | LBR | Overall | Overall | 21 | 2604 | 2598 | 31.7% | 1.14 (0.98 to 1.32) | 60% | 44 more per 1,000 (6 fewer per 1,000 to 101 more per 1,000) | -1 | -1 | 0 | -1 | -1 | 4 | Very low |
| Overall | Miscarriage rate | MR | Overall | Overall | 17 | 791 | 738 | 14.9% | 1.16 (0.93 to 1.46) | 0% | 24 more per 1,000 (10 fewer per 1,000 to 69 more per 1,000) | -1 | 0 | 0 | -1 | -1 | 3 | Very low |
| Overall | Ongoing pregnancy rate | OPR | Overall | Overall | 15 | 1656 | 1682 | 28.8% | 1.20 (0.93 to 1.54) | 71% | 58 more per 1,000 (20 fewer per 1,000 to 156 more per 1,000) | -1 | -1 | 0 | -1 | -1 | 4 | Very low |
| Overall | Implantation rate | IR | Overall | Overall | 17 | 3295 | 3215 | 25.8% | 1.16 (1.02 to 1.31) | 54% | 41 more per 1,000 (5 more per 1,000 to 80 more per 1,000) | -1 | -1 | 0 | 0 | -1 | 3 | Very low |
| Overall | Biochemical pregnancy rate | BPR | Overall | Overall | 16 | 2411 | 2714 | 47.0% | 1.10 (0.94 to 1.25) | 73% | 47 more per 1,000 (28 fewer per 1,000 to 117 more per 1,000) | -1 | -1 | 0 | -1 | 0 | 3 | Very low |
| Comparator subgroup | Clinical pregnancy rate | CPR | Control type | Sham acupuncture | 18 | 1854 | 1831 | 31.1% | 1.29 (1.10 to 1.51) | 61% | 90 more per 1,000 (31 more per 1,000 to 158 more per 1,000) | 0 | -1 | 0 | 0 | -1 | 2 | Low |
| Comparator subgroup | Clinical pregnancy rate | CPR | Control type | Blank / wait-list | 28 | 2760 | 3036 | 39.6% | 1.23 (1.11 to 1.36) | 47% | 91 more per 1,000 (44 more per 1,000 to 143 more per 1,000) | -1 | 0 | 0 | 0 | -0 | 1 | Moderate |
| Comparator subgroup | Live birth rate | LBR | Control type | Sham acupuncture | 9 | 1469 | 1466 | 29.1% | 1.00 (0.82 to 1.21) | 54% | 0 more per 1,000 (52 fewer per 1,000 to 61 more per 1,000) | 0 | -1 | 0 | -1 | Not formally assessed | 2 | Low |
| Comparator subgroup | Live birth rate | LBR | Control type | Blank / wait-list | 12 | 1135 | 1132 | 35.1% | 1.24 (1.02 to 1.51) | 54% | 84 more per 1,000 (7 more per 1,000 to 179 more per 1,000) | -1 | -1 | 0 | 0 | -0 | 2 | Low |
| Comparator subgroup | Miscarriage rate | MR | Control type | Sham acupuncture | 7 | 236 | 225 | 20.0% | 1.22 (0.88 to 1.70) | 0% | 44 more per 1,000 (24 fewer per 1,000 to 140 more per 1,000) | -1 | 0 | 0 | -1 | Not formally assessed | 2 | Low |
| Comparator subgroup | Miscarriage rate | MR | Control type | Blank / wait-list | 10 | 454 | 427 | 12.9% | 1.05 (0.73 to 1.50) | 0% | 6 more per 1,000 (35 fewer per 1,000 to 64 more per 1,000) | -1 | 0 | 0 | -1 | -1 | 3 | Very low |
| Comparator subgroup | Ongoing pregnancy rate | OPR | Control type | Sham acupuncture | 7 | 1164 | 1166 | 32.7% | 1.07 (0.77 to 1.47) | 72% | 23 more per 1,000 (75 fewer per 1,000 to 154 more per 1,000) | -1 | -1 | 0 | -1 | Not formally assessed | 3 | Very low |
| Comparator subgroup | Ongoing pregnancy rate | OPR | Control type | Blank / wait-list | 8 | 492 | 516 | 20.2% | 1.36 (0.94 to 1.97) | 56% | 73 more per 1,000 (12 fewer per 1,000 to 196 more per 1,000) | -1 | -1 | 0 | -1 | Not formally assessed | 3 | Very low |
| Comparator subgroup | Implantation rate | IR | Control type | Sham acupuncture | 5 | 872 | 875 | 25.8% | 1.08 (0.76 to 1.52) | 67% | 21 more per 1,000 (62 fewer per 1,000 to 134 more per 1,000) | -1 | -1 | 0 | -1 | Not formally assessed | 3 | Very low |
| Comparator subgroup | Implantation rate | IR | Control type | Blank / wait-list | 12 | 2423 | 2340 | 25.8% | 1.16 (1.06 to 1.28) | 40% | 41 more per 1,000 (15 more per 1,000 to 72 more per 1,000) | -1 | 0 | 0 | 0 | -1 | 2 | Low |
| Comparator subgroup | Biochemical pregnancy rate | BPR | Control type | Sham acupuncture | 11 | 814 | 802 | 41.0% | 1.16 (0.92 to 1.47) | 69% | 66 more per 1,000 (33 fewer per 1,000 to 193 more per 1,000) | -1 | -1 | 0 | -1 | -0 | 3 | Very low |
| Comparator subgroup | Biochemical pregnancy rate | BPR | Control type | Blank / wait-list | 5 | 1597 | 1912 | 49.5% | 0.99 (0.69 to 1.41) | 71% | 5 fewer per 1,000 (153 fewer per 1,000 to 203 more per 1,000) | -1 | -1 | 0 | -1 | Not formally assessed | 3 | Very low |
| Intervention timing subgroup | Clinical pregnancy rate | CPR | Intervention timing | Controlled ovarian hyperstimulation | 21 | 1151 | 1151 | 36.1% | 1.40 (1.25 to 1.57) | 12% | 144 more per 1,000 (90 more per 1,000 to 206 more per 1,000) | -1 | 0 | 0 | 0 | -1 | 2 | Low |
| Intervention timing subgroup | Clinical pregnancy rate | CPR | Intervention timing | Controlled ovarian hyperstimulation + embryo transfer | 6 | 788 | 777 | 21.0% | 1.35 (1.12 to 1.62) | 0% | 73 more per 1,000 (25 more per 1,000 to 130 more per 1,000) | 0 | 0 | 0 | 0 | Not formally assessed | 0 | High |
| Intervention timing subgroup | Clinical pregnancy rate | CPR | Intervention timing | Embryo transfer | 17 | 2468 | 2743 | 42.1% | 1.05 (0.92 to 1.19) | 63% | 21 more per 1,000 (34 fewer per 1,000 to 80 more per 1,000) | -1 | -1 | 0 | -1 | -0 | 3 | Very low |
| Intervention timing subgroup | Clinical pregnancy rate | CPR | Intervention timing | Luteal phase | 2 | 207 | 196 | 19.4% | 1.77 (1.25 to 2.52) | 6% | 149 more per 1,000 (48 more per 1,000 to 295 more per 1,000) | -1 | 0 | 0 | 0 | Not formally assessed | 1 | Moderate |
| Intervention timing subgroup | Live birth rate | LBR | Intervention timing | Controlled ovarian hyperstimulation | 7 | 538 | 524 | 32.8% | 1.41 (1.09 to 1.82) | 77% | 135 more per 1,000 (30 more per 1,000 to 269 more per 1,000) | -1 | -2 | 0 | 0 | Not formally assessed | 3 | Very low |
| Intervention timing subgroup | Live birth rate | LBR | Intervention timing | Controlled ovarian hyperstimulation + embryo transfer | 3 | 503 | 506 | 18.2% | 1.61 (0.90 to 2.89) | 15% | 111 more per 1,000 (18 fewer per 1,000 to 344 more per 1,000) | -1 | 0 | 0 | -1 | Not formally assessed | 2 | Low |
| Intervention timing subgroup | Live birth rate | LBR | Intervention timing | Embryo transfer | 11 | 1563 | 1568 | 35.7% | 0.96 (0.83 to 1.11) | 53% | 14 fewer per 1,000 (61 fewer per 1,000 to 39 more per 1,000) | -1 | -1 | 0 | -1 | -0 | 3 | Very low |
| Intervention timing subgroup | Miscarriage rate | MR | Intervention timing | Controlled ovarian hyperstimulation | 7 | 286 | 263 | 13.7% | 0.82 (0.82 to 1.53) | 0% | 25 fewer per 1,000 (25 fewer per 1,000 to 73 more per 1,000) | -1 | 0 | 0 | -1 | Not formally assessed | 2 | Low |
| Intervention timing subgroup | Miscarriage rate | MR | Intervention timing | Controlled ovarian hyperstimulation + embryo transfer | 4 | 129 | 133 | 8.3% | 1.38 (0.73 to 2.61) | 10% | 31 more per 1,000 (22 fewer per 1,000 to 133 more per 1,000) | -1 | 0 | 0 | -1 | Not formally assessed | 2 | Low |
| Intervention timing subgroup | Miscarriage rate | MR | Intervention timing | Embryo transfer | 4 | 200 | 215 | 21.4% | 1.05 (0.73 to 1.50) | 0% | 11 more per 1,000 (58 fewer per 1,000 to 107 more per 1,000) | -1 | 0 | 0 | -1 | Not formally assessed | 2 | Low |
| Intervention timing subgroup | Miscarriage rate | MR | Intervention timing | Luteal phase | 2 | 75 | 41 | 17.1% | 1.52 (0.70 to 3.29) | 0% | 89 more per 1,000 (51 fewer per 1,000 to 391 more per 1,000) | -1 | 0 | 0 | -1 | Not formally assessed | 2 | Low |
| Intervention timing subgroup | Ongoing pregnancy rate | OPR | Intervention timing | Controlled ovarian hyperstimulation | 2 | 61 | 91 | 15.4% | 2.46 (1.36 to 4.44) | 0% | 225 more per 1,000 (55 more per 1,000 to 529 more per 1,000) | -1 | 0 | 0 | 0 | Not formally assessed | 1 | Moderate |
| Intervention timing subgroup | Ongoing pregnancy rate | OPR | Intervention timing | Controlled ovarian hyperstimulation + embryo transfer | 3 | 223 | 231 | 16.5% | 1.58 (1.10 to 2.28) | 0% | 95 more per 1,000 (16 more per 1,000 to 211 more per 1,000) | -1 | 0 | 0 | 0 | Not formally assessed | 1 | Moderate |
| Intervention timing subgroup | Ongoing pregnancy rate | OPR | Intervention timing | Embryo transfer | 8 | 1165 | 1164 | 34.3% | 0.92 (0.70 to 1.21) | 65% | 27 fewer per 1,000 (103 fewer per 1,000 to 72 more per 1,000) | -1 | -1 | 0 | -1 | Not formally assessed | 3 | Very low |
| Intervention timing subgroup | Ongoing pregnancy rate | OPR | Intervention timing | Luteal phase | 2 | 207 | 196 | 17.3% | 1.57 (0.93 to 2.66) | 48% | 99 more per 1,000 (12 fewer per 1,000 to 288 more per 1,000) | -1 | 0 | 0 | -1 | Not formally assessed | 2 | Low |
| Intervention timing subgroup | Implantation rate | IR | Intervention timing | Controlled ovarian hyperstimulation | 7 | 959 | 942 | 28.0% | 1.62 (1.17 to 2.24) | 65% | 174 more per 1,000 (48 more per 1,000 to 348 more per 1,000) | -1 | -1 | 0 | 0 | Not formally assessed | 2 | Low |
| Intervention timing subgroup | Implantation rate | IR | Intervention timing | Embryo transfer | 8 | 2028 | 1986 | 26.3% | 1.05 (0.90 to 1.23) | 51% | 13 more per 1,000 (26 fewer per 1,000 to 61 more per 1,000) | -1 | -1 | 0 | -1 | Not formally assessed | 3 | Very low |
| Intervention timing subgroup | Implantation rate | IR | Intervention timing | Luteal phase | 2 | 308 | 287 | 14.6% | 1.04 (0.71 to 1.52) | 0% | 6 more per 1,000 (42 fewer per 1,000 to 76 more per 1,000) | -1 | 0 | 0 | -1 | Not formally assessed | 2 | Low |
| Intervention timing subgroup | Biochemical pregnancy rate | BPR | Intervention timing | Controlled ovarian hyperstimulation | 2 | 367 | 399 | 47.4% | 1.35 (0.77 to 2.35) | 72% | 166 more per 1,000 (109 fewer per 1,000 to 639 more per 1,000) | -1 | -1 | 0 | -1 | Not formally assessed | 3 | Very low |
| Intervention timing subgroup | Biochemical pregnancy rate | BPR | Intervention timing | Controlled ovarian hyperstimulation + embryo transfer | 3 | 129 | 133 | 28.6% | 1.59 (1.13 to 2.24) | 0% | 169 more per 1,000 (37 more per 1,000 to 354 more per 1,000) | -1 | 0 | 0 | 0 | Not formally assessed | 1 | Moderate |
| Intervention timing subgroup | Biochemical pregnancy rate | BPR | Intervention timing | Embryo transfer | 9 | 1708 | 1986 | 50.7% | 0.88 (0.74 to 1.60) | 73% | 61 fewer per 1,000 (132 fewer per 1,000 to 304 more per 1,000) | -1 | -1 | 0 | -1 | Not formally assessed | 3 | Very low |
| Intervention timing subgroup | Biochemical pregnancy rate | BPR | Intervention timing | Luteal phase | 2 | 207 | 196 | 21.4% | 1.72 (1.16 to 2.54) | 32% | 154 more per 1,000 (34 more per 1,000 to 330 more per 1,000) | -1 | 0 | 0 | 0 | Not formally assessed | 1 | Moderate |

1. **Supplemental File 10. Publication bias assessment**

Supplemental Figure 18. Funnel plot for OPR (ongoing pregnancy rate).


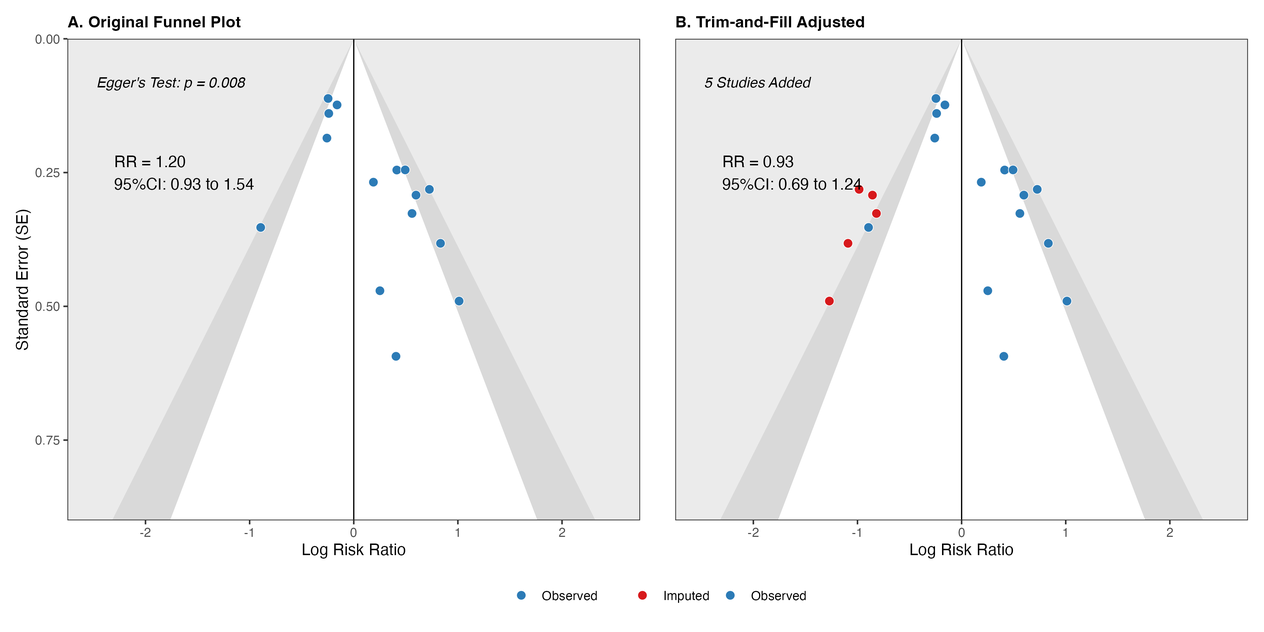


Supplemental Figure 19. Funnel plot for BPR (biochemical pregnancy rate).


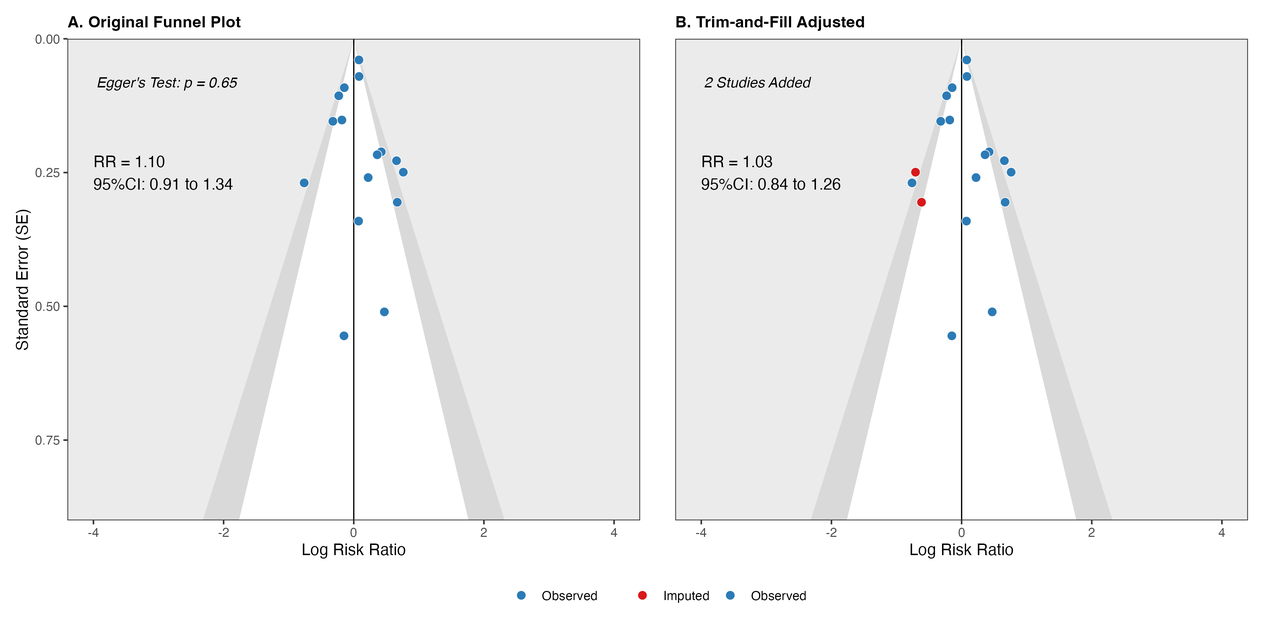


Supplemental Figure 20. Funnel plot for IR (implantation rate).


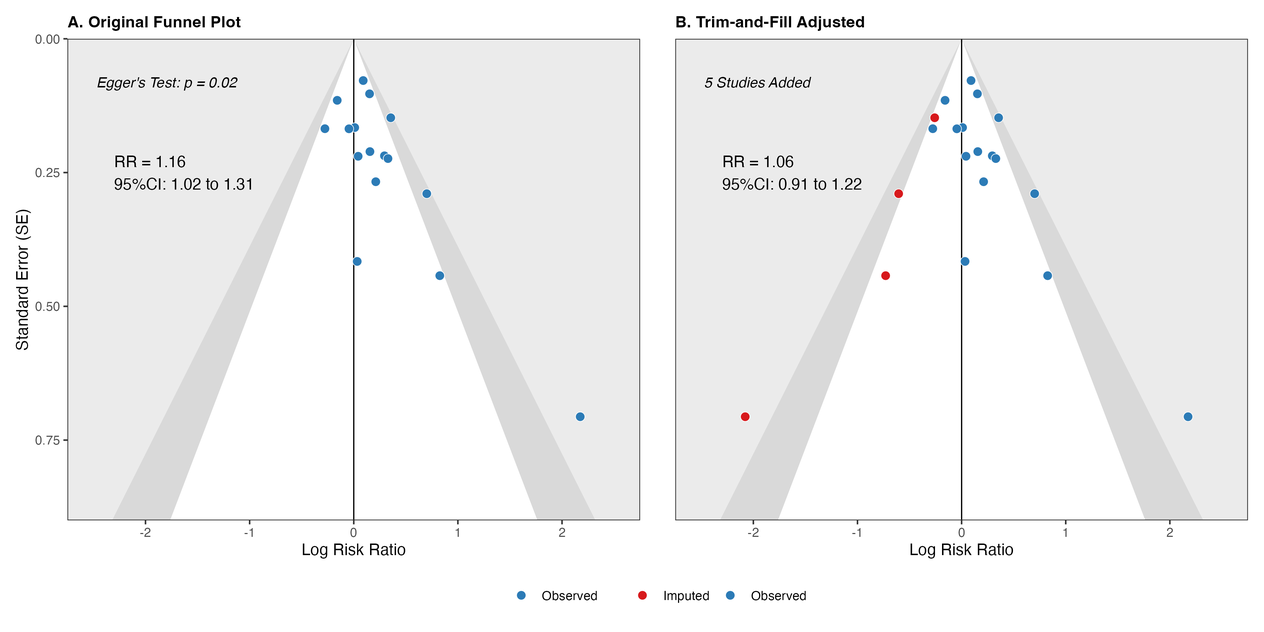


Supplemental Figure 21. Funnel plot for MR (miscarriage rate).


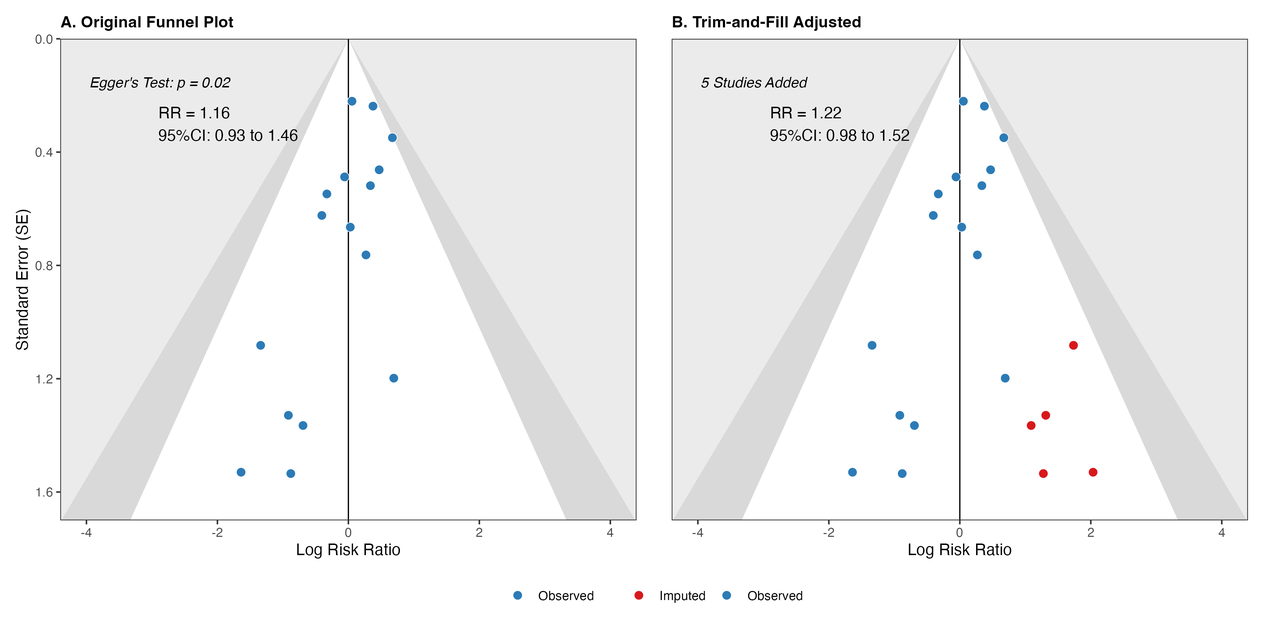


1. **Supplemental File 11. Subgroup and meta-regression summary for potential effect modifiers**

Supplemental Table 13. Summary of subgroup analyses and univariable meta-regression for potential effect modifiers across reproductive outcomes.

*Subgroup and meta-regression summary*

| **Outcome** | **Modifier** | **Analysis type** | **No. of comparisons/effect estimates, k** | **Levels / range** | **P for subgroup difference or meta-regression** | **Unadjusted tau2** | **Residual tau2** | **tau2 reduction / R2 analogue** | **Interpretation** |
| --- | --- | --- | --- | --- | --- | --- | --- | --- | --- |
| CPR | transfer type | Subgroup analysis / meta-regression | 46 | Fresh ET, k = 30; Mixed ET, k = 13; FET, k = 3 | 0.866 | 0.0457 | 0.0499 | 0% / no reduction | No statistically clear effect modification |
| CPR | acupuncture modality | Subgroup analysis / meta-regression | 46 | Manual acupuncture, k = 23; Electroacupuncture, k = 12; TEAS, k = 11; | 0.561 | 0.0457 | 0.0489 | 0% / no reduction | No statistically clear effect modification |
| CPR | comparator type | Subgroup analysis / meta-regression | 46 | inactive control, k = 28; sham control, k = 18 | 0.793 | 0.0457 | 0.049 | 0% / no reduction | No statistically clear effect modification |
| CPR | intervention timing | Subgroup analysis / meta-regression | 46 | COH, k = 21; ET, k = 17; COH + ET, k = 6; Luteal phase, k = 2 | <0.001 | 0.0457 | 0.0206 | 54.9% | Exploratory evidence of effect modification |
| CPR | age | Univariable meta-regression | 44 | Range: 28.80 to 38.05 | 0.632 | 0.0427 | 0.0441 | 0% / no reduction | No statistically clear effect modification |
| CPR | BMI | Univariable meta-regression | 36 | Range: 21.65 to 32.90 | 0.530 | 0.0375 | 0.0375 | 0.1% | No statistically clear effect modification |
| CPR | infertility duration | Univariable meta-regression | 32 | Range: 2.23 to 9.25 | 0.448 | 0.0493 | 0.0497 | 0% / no reduction | No statistically clear effect modification |
| CPR | primary infertility proportion | Univariable meta-regression | 20 | Range: 11.50 to 82.70 | 0.281 | 0.0759 | 0.0749 | 1.3% | No statistically clear effect modification |
| LBR | transfer type | Subgroup analysis / meta-regression | 21 | Fresh ET, k = 13; Mixed ET, k = 7; FET, k = 1 | 0.604 | 0.0659 | 0.0787 | 0% / no reduction | No statistically clear effect modification |
| LBR | acupuncture modality | Subgroup analysis / meta-regression | 21 | Manual acupuncture, k = 11; Electroacupuncture, k = 5; TEAS, k = 5; | 0.130 | 0.0659 | 0.0459 | 30.4% | No statistically clear effect modification |
| LBR | comparator type | Subgroup analysis / meta-regression | 21 | inactive control, k = 12; sham control, k = 9 | 0.151 | 0.0659 | 0.0541 | 18.0% | No statistically clear effect modification |
| LBR | intervention timing | Subgroup analysis / meta-regression | 21 | COH, k = 7; ET, k = 11; COH + ET, k = 3; Luteal phase, k = 0 | 0.015 | 0.0659 | 0.046 | 30.2% | Exploratory evidence of effect modification |
| LBR | age | Univariable meta-regression | 21 | Range: 28.80 to 36.00 | 0.046 | 0.0659 | 0.0443 | 32.8% | Exploratory evidence of effect modification |
| LBR | BMI | Univariable meta-regression | 18 | Range: 21.65 to 32.90 | 0.049 | 0.0624 | 0.0402 | 35.6% | Exploratory evidence of effect modification |
| LBR | infertility duration | Univariable meta-regression | 14 | Range: 2.50 to 6.45 | 0.497 | 0.0399 | 0.0416 | 0% / no reduction | No statistically clear effect modification |
| LBR | primary infertility proportion | Univariable meta-regression | 9 | Range: 11.50 to 75.40 | 0.500 | 0.1558 | 0.165 | 0% / no reduction | No statistically clear effect modification; limited by sparse data |
| OPR | transfer type | Subgroup analysis / meta-regression | 15 | Fresh ET, k = 10; Mixed ET, k = 4; FET, k = 1 | 0.451 | 0.1601 | 0.1816 | 0% / no reduction | No statistically clear effect modification |
| OPR | acupuncture modality | Subgroup analysis / meta-regression | 15 | Manual acupuncture, k = 14; Electroacupuncture, k = 1 | 0.453 | 0.1601 | 0.1641 | 0% / no reduction | No statistically clear effect modification |
| OPR | comparator type | Subgroup analysis / meta-regression | 15 | inactive control, k = 8; sham control, k = 7 | 0.337 | 0.1601 | 0.1484 | 7.3% | No statistically clear effect modification |
| OPR | intervention timing | Subgroup analysis / meta-regression | 15 | COH, k = 2; ET, k = 8; COH + ET, k = 3; Luteal phase, k = 2 | 0.009 | 0.1601 | 0.064 | 60.0% | Exploratory evidence of effect modification |
| OPR | age | Univariable meta-regression | 13 | Range: 30.90 to 37.51 | 0.325 | 0.1491 | 0.156 | 0% / no reduction | No statistically clear effect modification |
| OPR | BMI | Univariable meta-regression | 12 | Range: 21.65 to 26.96 | 0.596 | 0.1576 | 0.1647 | 0% / no reduction | No statistically clear effect modification |
| OPR | infertility duration | Univariable meta-regression | 11 | Range: 2.50 to 9.25 | 0.983 | 0.1461 | 0.1706 | 0% / no reduction | No statistically clear effect modification |
| OPR | primary infertility proportion | Univariable meta-regression | 9 | Range: 23.50 to 82.70 | 0.593 | 0.1391 | 0.1644 | 0% / no reduction | No statistically clear effect modification; limited by sparse data |
| BPR | transfer type | Subgroup analysis / meta-regression | 16 | Fresh ET, k = 9; Mixed ET, k = 6; FET, k = 1 | 0.570 | 0.1078 | 0.1227 | 0% / no reduction | No statistically clear effect modification |
| BPR | acupuncture modality | Subgroup analysis / meta-regression | 16 | Manual acupuncture, k = 13; TEAS, k = 2; Electroacupuncture, k = 1 | 0.961 | 0.1078 | 0.1368 | 0% / no reduction | No statistically clear effect modification |
| BPR | comparator type | Subgroup analysis / meta-regression | 16 | inactive control, k = 11; sham control, k = 5 | 0.450 | 0.1078 | 0.112 | 0% / no reduction | No statistically clear effect modification |
| BPR | intervention timing | Subgroup analysis / meta-regression | 16 | COH, k = 2; ET, k = 9; COH + ET, k = 3; Luteal phase, k = 2 | 0.005 | 0.1078 | 0.0459 | 57.4% | Exploratory evidence of effect modification |
| BPR | age | Univariable meta-regression | 15 | Range: 31.00 to 37.00 | 0.193 | 0.0991 | 0.0978 | 1.3% | No statistically clear effect modification |
| BPR | BMI | Univariable meta-regression | 14 | Range: 21.65 to 32.90 | 0.685 | 0.0962 | 0.1055 | 0% / no reduction | No statistically clear effect modification |
| BPR | infertility duration | Univariable meta-regression | 11 | Range: 2.50 to 9.25 | 0.704 | 0.0903 | 0.104 | 0% / no reduction | No statistically clear effect modification |
| BPR | primary infertility proportion | Univariable meta-regression | 11 | Range: 23.50 to 82.70 | 0.282 | 0.1366 | 0.1358 | 0.6% | No statistically clear effect modification |
| IR | transfer type | Subgroup analysis / meta-regression | 17 | Fresh ET, k = 11; FET, k = 3; Mixed ET, k = 3 | 0.859 | 0.0268 | 0.0432 | 0% / no reduction | No statistically clear effect modification |
| IR | acupuncture modality | Subgroup analysis / meta-regression | 17 | Manual acupuncture, k = 9; TEAS, k = 5; Electroacupuncture, k = 3; | 0.012 | 0.0268 | 0.0211 | 21.3% | Exploratory evidence of effect modification |
| IR | comparator type | Subgroup analysis / meta-regression | 17 | inactive control, k = 12; sham control, k = 5 | 0.159 | 0.0268 | 0.0172 | 35.8% | No statistically clear effect modification |
| IR | intervention timing | Subgroup analysis / meta-regression | 17 | COH, k = 7; ET, k = 8; COH + ET, k = 0; Luteal phase, k = 2 | 0.074 | 0.0268 | 0.0359 | 0% / no reduction | No statistically clear effect modification |
| IR | age | Univariable meta-regression | 17 | Range: 29.63 to 37.00 | 0.246 | 0.0268 | 0.0202 | 24.7% | No statistically clear effect modification |
| IR | BMI | Univariable meta-regression | 15 | Range: 21.65 to 24.31 | 0.004 | 0.0281 | 0 | 100.0% | Exploratory evidence of effect modification |
| IR | infertility duration | Univariable meta-regression | 14 | Range: 3.10 to 6.39 | 0.506 | 0.0343 | 0.0452 | 0% / no reduction | No statistically clear effect modification |
| IR | primary infertility proportion | Univariable meta-regression | 8 | Range: 11.48 to 79.55 | 0.493 | 0.0973 | 0.1202 | 0% / no reduction | No statistically clear effect modification; limited by sparse data |
| MR | transfer type | Subgroup analysis / meta-regression | 17 | Fresh ET, k = 11; Mixed ET, k = 4; FET, k = 2 | 0.805 | 0 | 0 | Not estimable | No statistically clear effect modification |
| MR | acupuncture modality | Subgroup analysis / meta-regression | 17 | Manual acupuncture, k = 10; Electroacupuncture, k = 5; TEAS, k = 2 | 0.079 | 0 | 0 | Not estimable | No statistically clear effect modification |
| MR | comparator type | Subgroup analysis / meta-regression | 17 | inactive control, k = 10; sham control, k = 7 | 0.577 | 0 | 0.0153 | Not estimable | No statistically clear effect modification |
| MR | intervention timing | Subgroup analysis / meta-regression | 17 | COH, k = 7; ET, k = 4; COH + ET, k = 4; Luteal phase, k = 2 | 0.688 | 0 | 0.0317 | Not estimable | No statistically clear effect modification |
| MR | age | Univariable meta-regression | 16 | Range: 29.29 to 37.00 | 0.941 | 0 | 0 | Not estimable | No statistically clear effect modification |
| MR | BMI | Univariable meta-regression | 16 | Range: 21.66 to 32.90 | 0.918 | 0 | 0 | Not estimable | No statistically clear effect modification |
| MR | infertility duration | Univariable meta-regression | 11 | Range: 3.38 to 9.25 | 0.501 | 0 | 0 | Not estimable | No statistically clear effect modification |
| MR | primary infertility proportion | Univariable meta-regression | 12 | Range: 11.20 to 79.78 | 0.525 | 0 | 0 | Not estimable | No statistically clear effect modification |

Note: NR, not reported. Variables with sufficient and consistent reporting were included in subgroup analyses, meta-regression, or meta-CART. Variables with sparse or inconsistent reporting were summarized descriptively and considered in the interpretation of heterogeneity and certainty of evidence. The column k denotes the number of comparisons/effect estimates included in the model, not necessarily the number of unique studies. Because these analyses were based on aggregate study-level data and were not adjusted for multiplicity, statistically significant findings should be interpreted as exploratory. Meta-regression results based on fewer than 10 comparisons were considered unstable and interpreted descriptively. Unadjusted tau2 was calculated using the same subset of comparisons included in each moderator model. Values shown as 0% / no reduction indicate that adding the moderator did not reduce between-study heterogeneity.

1. **Supplemental File 12. Exploratory timing-dose meta-regression**

Supplemental Figure 22. Exploratory timing-dose meta-regression for clinical pregnancy and live birth rates. A: distribution of acupuncture session numbers by intervention timing node; B: study-level session-effect associations based on log risk ratios.


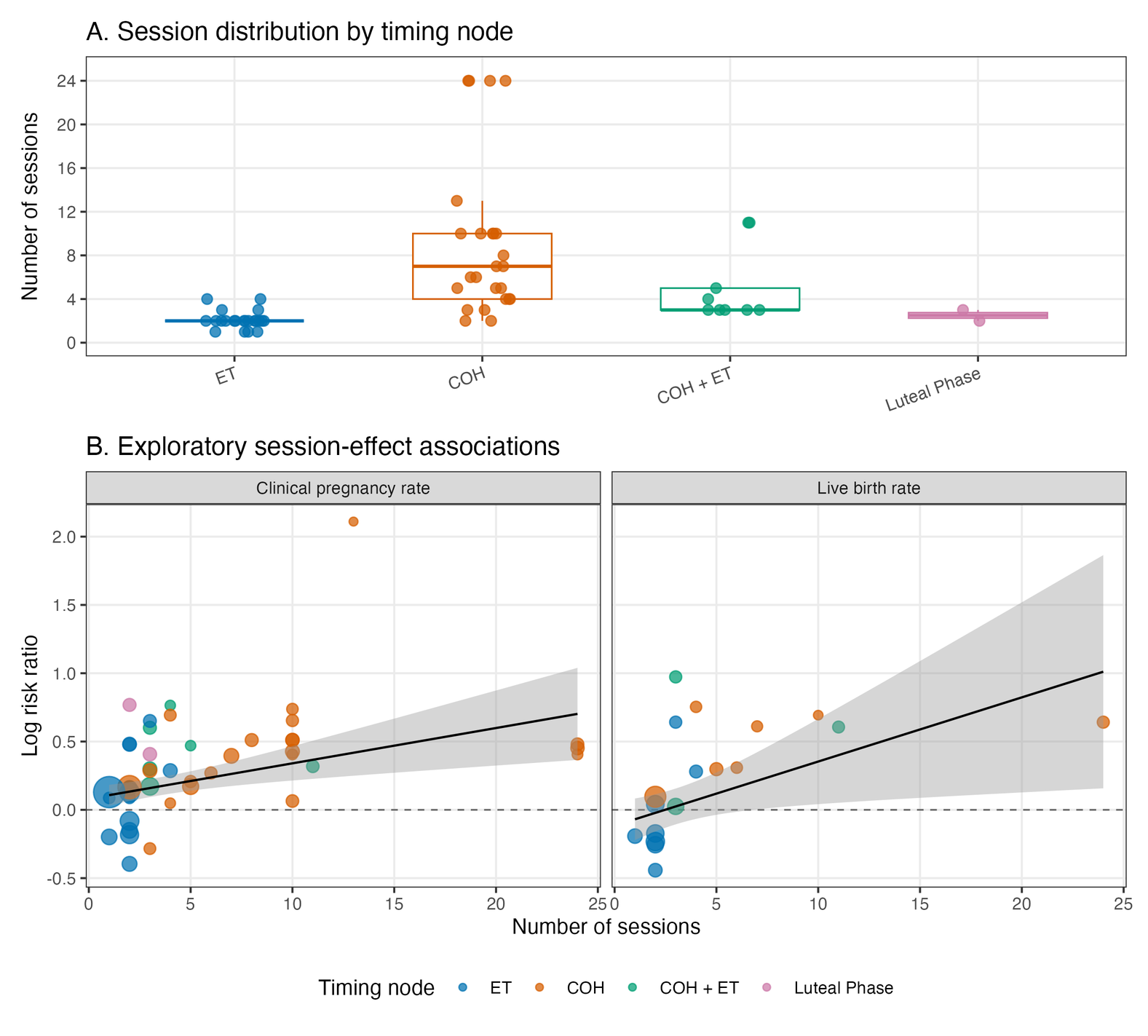


Supplemental Table 14. Study-level exploratory timing-dose meta-regression summary for clinical pregnancy and live birth rates.

| **Outcome** | **Model** | **Timing** | **k** | **median_session** | **min_session** | **max_session** | **Term** | **estimate** | **se** | **z** | **p** | **ci_lb** | **ci_ub** | **omnibus_p** | **tau2** |
| --- | --- | --- | --- | --- | --- | --- | --- | --- | --- | --- | --- | --- | --- | --- | --- |
| CPR | Session distribution | ET | 14 | 2 | 1 | 4 | NA | NA | NA | NA | NA | NA | NA | NA | NA |
| CPR | Session distribution | COH | 21 | 10 | 2 | 24 | NA | NA | NA | NA | NA | NA | NA | NA | NA |
| CPR | Session distribution | COH + ET | 6 | 3.5 | 3 | 11 | NA | NA | NA | NA | NA | NA | NA | NA | NA |
| CPR | Session distribution | Luteal Phase | 2 | 2.5 | 2 | 3 | NA | NA | NA | NA | NA | NA | NA | NA | NA |
| CPR | Timing only | NA | 43 | NA | NA | NA | intrcpt | 0.0551 | 0.0572 | 0.9642 | 0.3349 | -0.057 | 0.1673 | 0.0009 | 0.0172 |
| CPR | Timing only | NA | 43 | NA | NA | NA | TimingCOH | 0.2902 | 0.0848 | 3.4209 | 0.0006 | 0.124 | 0.4565 | 0.0009 | 0.0172 |
| CPR | Timing only | NA | 43 | NA | NA | NA | TimingCOH + ET | 0.2739 | 0.1294 | 2.1173 | 0.0342 | 0.0204 | 0.5274 | 0.0009 | 0.0172 |
| CPR | Timing only | NA | 43 | NA | NA | NA | TimingLuteal Phase | 0.5195 | 0.2055 | 2.5282 | 0.0115 | 0.1168 | 0.9222 | 0.0009 | 0.0172 |
| CPR | Session only | NA | 43 | NA | NA | NA | intrcpt | 0.1105 | 0.0559 | 1.9763 | 0.0481 | 0.0009 | 0.2201 | 0.0023 | 0.0223 |
| CPR | Session only | NA | 43 | NA | NA | NA | Session | 0.0242 | 0.008 | 3.0422 | 0.0023 | 0.0086 | 0.0398 | 0.0023 | 0.0223 |
| CPR | Timing + session | NA | 43 | NA | NA | NA | intrcpt | 0.0215 | 0.0557 | 0.3863 | 0.6993 | -0.0876 | 0.1306 | 0.0002 | 0.0125 |
| CPR | Timing + session | NA | 43 | NA | NA | NA | TimingCOH | 0.1813 | 0.0977 | 1.8555 | 0.0635 | -0.0102 | 0.3728 | 0.0002 | 0.0125 |
| CPR | Timing + session | NA | 43 | NA | NA | NA | TimingCOH + ET | 0.23 | 0.1244 | 1.8485 | 0.0645 | -0.0139 | 0.4738 | 0.0002 | 0.0125 |
| CPR | Timing + session | NA | 43 | NA | NA | NA | TimingLuteal Phase | 0.5095 | 0.1985 | 2.5662 | 0.0103 | 0.1204 | 0.8986 | 0.0002 | 0.0125 |
| CPR | Timing + session | NA | 43 | NA | NA | NA | Session | 0.0169 | 0.0091 | 1.8595 | 0.063 | -0.0009 | 0.0347 | 0.0002 | 0.0125 |
| CPR | Timing + session + comparator | NA | 43 | NA | NA | NA | intrcpt | 0.0646 | 0.0487 | 1.3281 | 0.1841 | -0.0308 | 0.1601 | 1.02628523135437e-05 | 0.0034 |
| CPR | Timing + session + comparator | NA | 43 | NA | NA | NA | TimingCOH | 0.1256 | 0.0826 | 1.5199 | 0.1285 | -0.0364 | 0.2875 | 1.02628523135437e-05 | 0.0034 |
| CPR | Timing + session + comparator | NA | 43 | NA | NA | NA | TimingCOH + ET | 0.2432 | 0.1097 | 2.2173 | 0.0266 | 0.0282 | 0.4582 | 1.02628523135437e-05 | 0.0034 |
| CPR | Timing + session + comparator | NA | 43 | NA | NA | NA | TimingLuteal Phase | 0.5109 | 0.184 | 2.7774 | 0.0055 | 0.1504 | 0.8715 | 1.02628523135437e-05 | 0.0034 |
| CPR | Timing + session + comparator | NA | 43 | NA | NA | NA | Session | 0.0236 | 0.0086 | 2.7379 | 0.0062 | 0.0067 | 0.0405 | 1.02628523135437e-05 | 0.0034 |
| CPR | Timing + session + comparator | NA | 43 | NA | NA | NA | ComparatorSham/superficial control | -0.1398 | 0.0684 | -2.0425 | 0.0411 | -0.2739 | -0.0056 | 1.02628523135437e-05 | 0.0034 |
| LBR | Session distribution | ET | 8 | 2 | 1 | 4 | NA | NA | NA | NA | NA | NA | NA | NA | NA |
| LBR | Session distribution | COH | 7 | 6 | 2 | 24 | NA | NA | NA | NA | NA | NA | NA | NA | NA |
| LBR | Session distribution | COH + ET | 3 | 3 | 3 | 11 | NA | NA | NA | NA | NA | NA | NA | NA | NA |
| LBR | Timing only | NA | 18 | NA | NA | NA | intrcpt | -0.0944 | 0.1009 | -0.9353 | 0.3496 | -0.2921 | 0.1034 | 0.0083 | 0.0485 |
| LBR | Timing only | NA | 18 | NA | NA | NA | TimingCOH | 0.4583 | 0.1736 | 2.6392 | 0.0083 | 0.1179 | 0.7986 | 0.0083 | 0.0485 |
| LBR | Timing only | NA | 18 | NA | NA | NA | TimingCOH + ET | 0.4918 | 0.2166 | 2.2707 | 0.0232 | 0.0673 | 0.9163 | 0.0083 | 0.0485 |
| LBR | Session only | NA | 18 | NA | NA | NA | intrcpt | -0.0625 | 0.0955 | -0.6549 | 0.5126 | -0.2497 | 0.1246 | 0.007 | 0.0421 |
| LBR | Session only | NA | 18 | NA | NA | NA | Session | 0.0436 | 0.0162 | 2.6962 | 0.007 | 0.0119 | 0.0754 | 0.007 | 0.0421 |
| LBR | Timing + session | NA | 18 | NA | NA | NA | intrcpt | -0.1641 | 0.0936 | -1.7529 | 0.0796 | -0.3475 | 0.0194 | 0.0027 | 0.0287 |
| LBR | Timing + session | NA | 18 | NA | NA | NA | TimingCOH | 0.3143 | 0.1684 | 1.8665 | 0.062 | -0.0157 | 0.6444 | 0.0027 | 0.0287 |
| LBR | Timing + session | NA | 18 | NA | NA | NA | TimingCOH + ET | 0.3929 | 0.1955 | 2.0093 | 0.0445 | 0.0097 | 0.7762 | 0.0027 | 0.0287 |
| LBR | Timing + session | NA | 18 | NA | NA | NA | Session | 0.0285 | 0.017 | 1.6745 | 0.094 | -0.0049 | 0.062 | 0.0027 | 0.0287 |
| LBR | Timing + session + comparator | NA | 18 | NA | NA | NA | intrcpt | -0.0547 | 0.111 | -0.4927 | 0.6222 | -0.2722 | 0.1629 | 0.0005 | 0.0124 |
| LBR | Timing + session + comparator | NA | 18 | NA | NA | NA | TimingCOH | 0.186 | 0.1534 | 1.2126 | 0.2253 | -0.1146 | 0.4866 | 0.0005 | 0.0124 |
| LBR | Timing + session + comparator | NA | 18 | NA | NA | NA | TimingCOH + ET | 0.3329 | 0.1688 | 1.9724 | 0.0486 | 0.0021 | 0.6637 | 0.0005 | 0.0124 |
| LBR | Timing + session + comparator | NA | 18 | NA | NA | NA | Session | 0.0361 | 0.0157 | 2.2926 | 0.0219 | 0.0052 | 0.067 | 0.0005 | 0.0124 |
| LBR | Timing + session + comparator | NA | 18 | NA | NA | NA | ComparatorSham/superficial control | -0.1957 | 0.1234 | -1.5865 | 0.1126 | -0.4375 | 0.0461 | 0.0005 | 0.0124 |

1. **Supplemental File 13. Continuous moderator analysis using Meta-CART**

Meta-CART used Age_Pooled, BMI_Pooled, PI_Pooled, and Duration_Pooled as candidate continuous moderators when corresponding output files were available.

Supplemental Figure 23. Continuous-variable Meta-CART analysis for CPR. No moderator effect was detected.


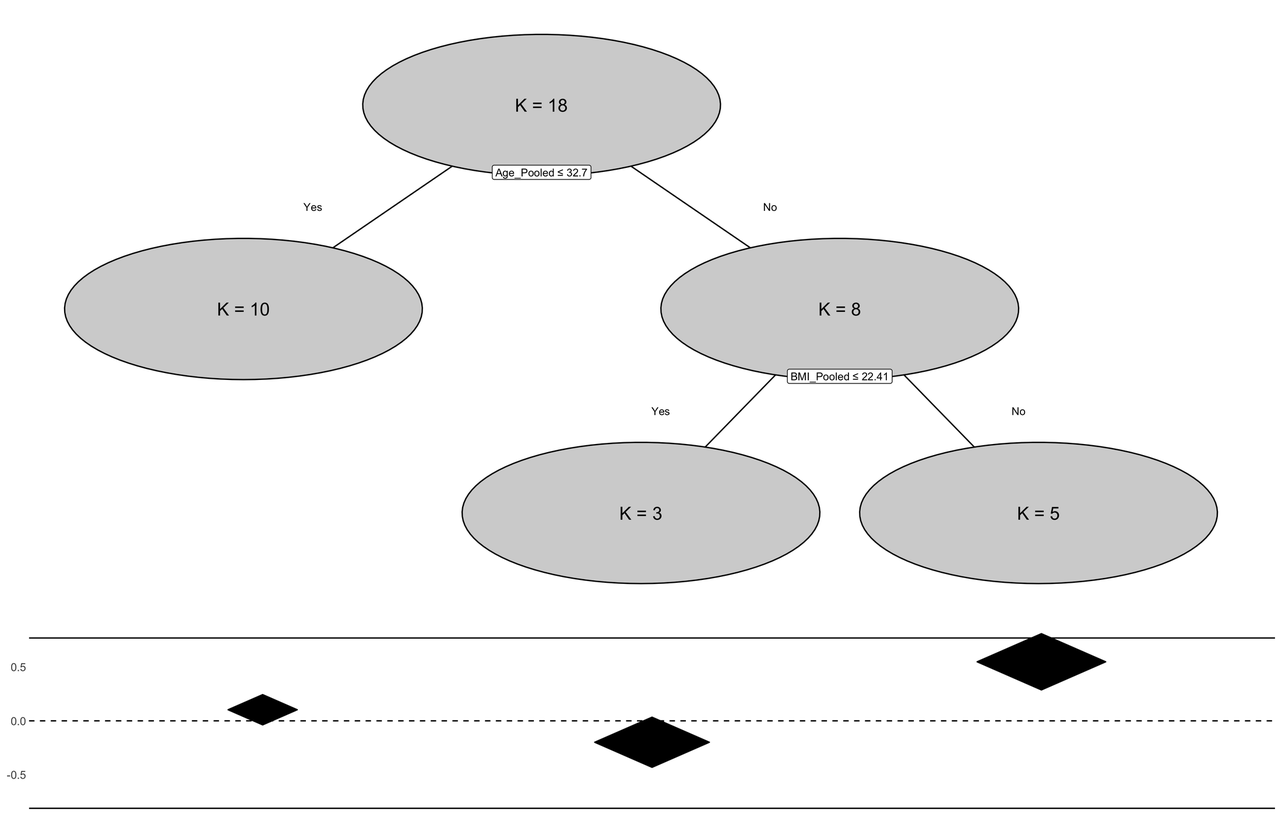


Supplemental Figure 24. Continuous-variable Meta-CART analysis for LBR. Age_Pooled and Duration_Pooled were identified as potential moderators.


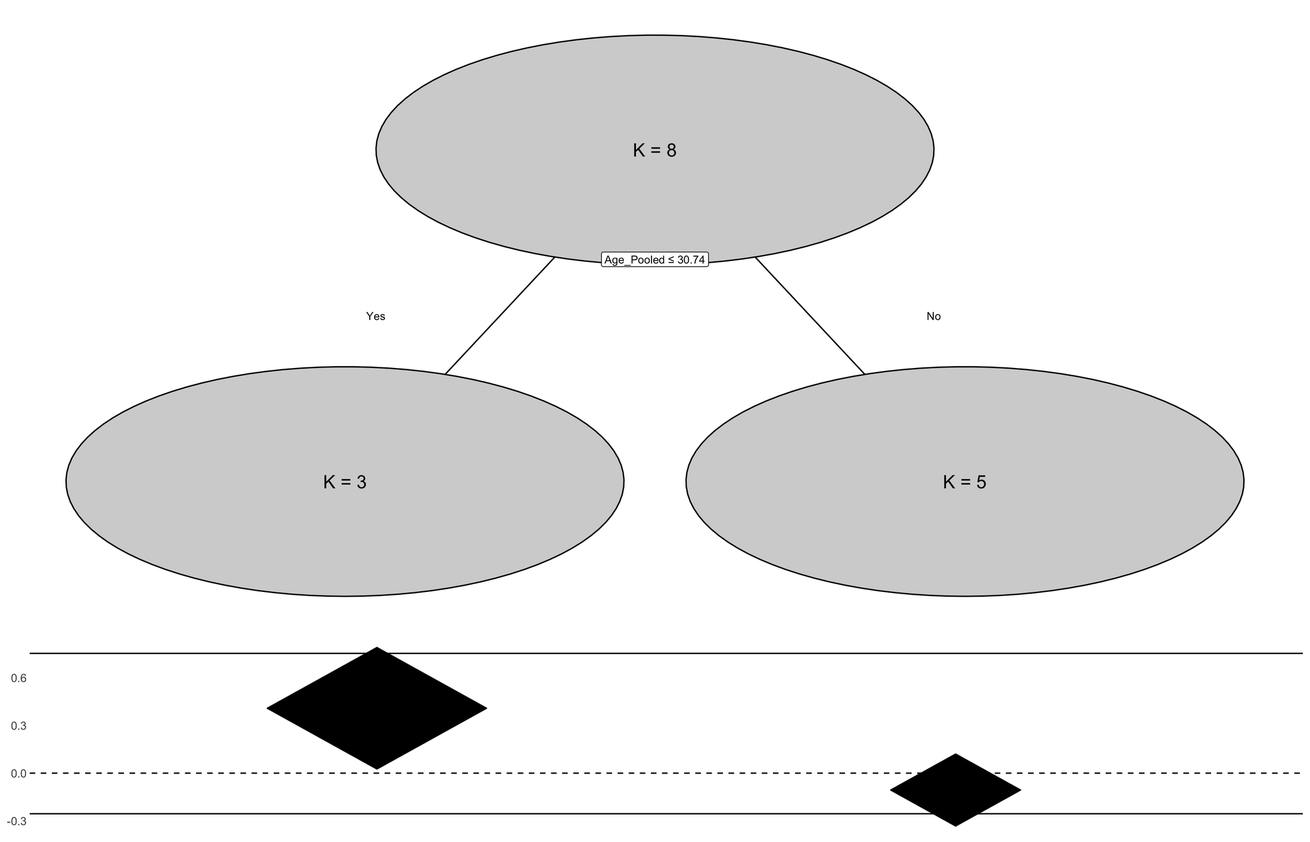


Supplemental Figure 25. Continuous-variable Meta-CART analysis for MR. BMI_Pooled was identified as a potential moderator.


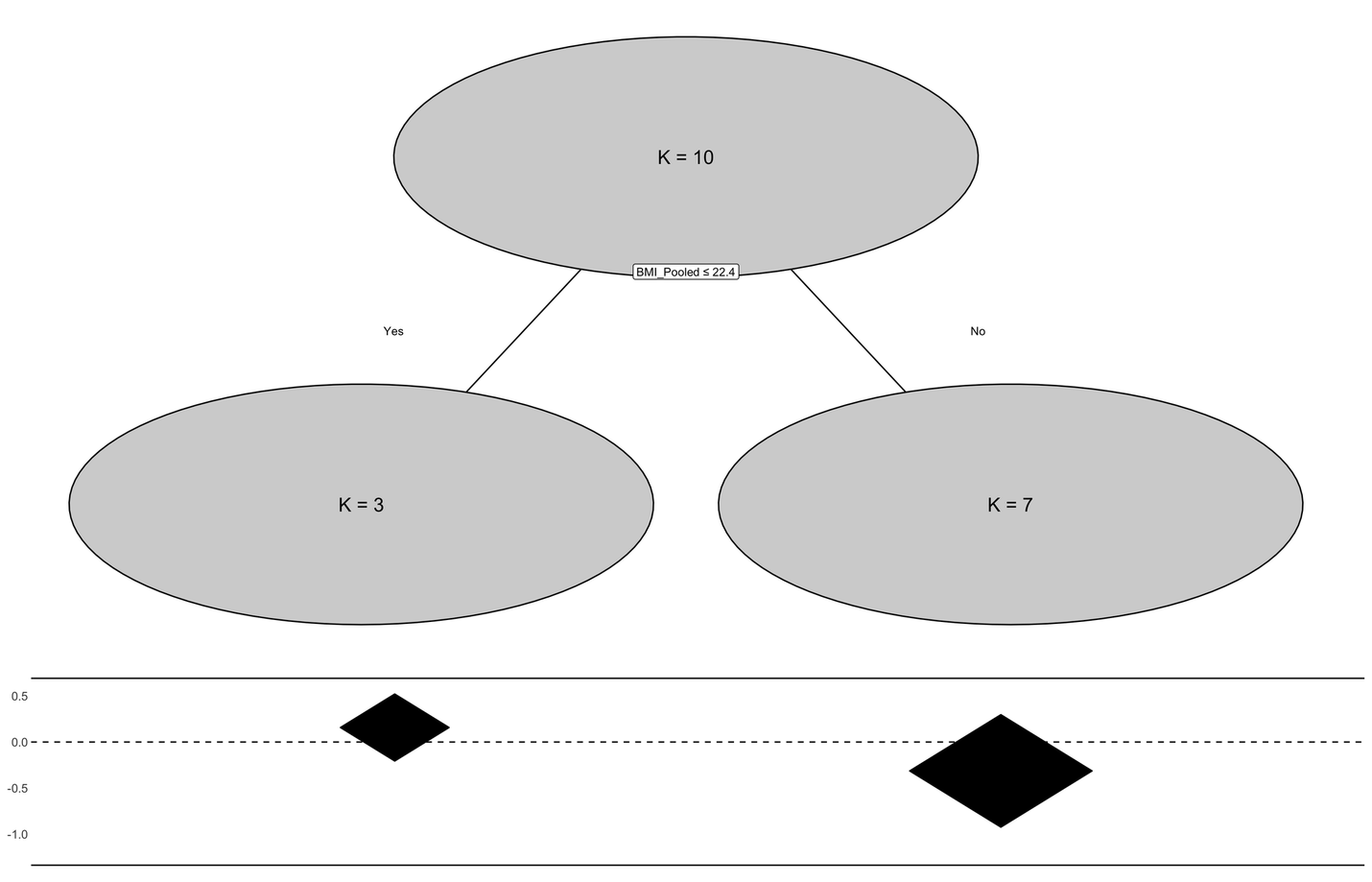


1. **Supplemental File 14. Sensitivity analyses for conventional pairwise meta-analysis**

Supplemental Figure 26. Sensitivity analysis for CPR (clinical pregnancy rate).


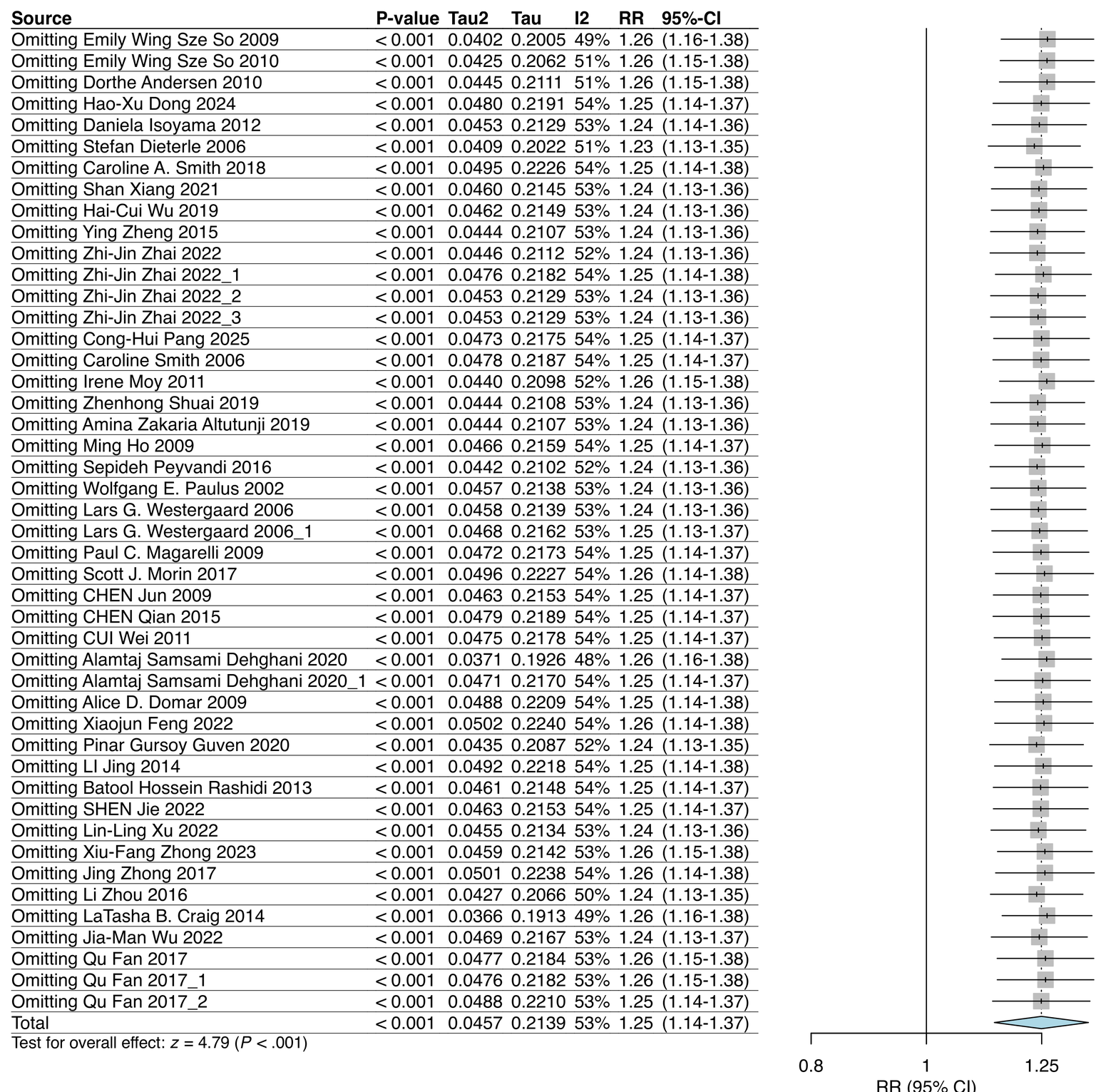


Supplemental Figure 27. Sensitivity analysis for LBR (live birth rate).


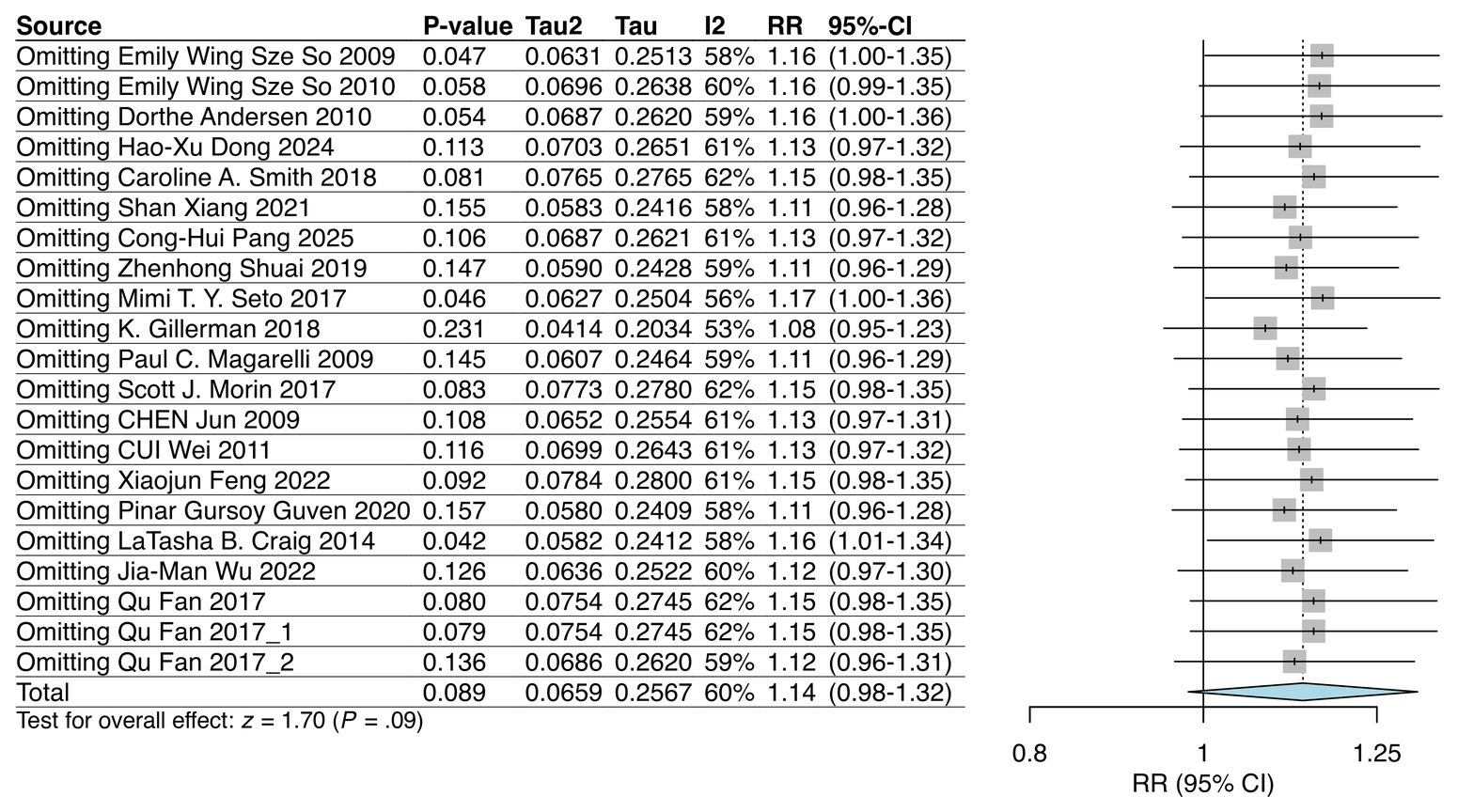


Supplemental Figure 28. Sensitivity analysis for OPR (ongoing pregnancy rate).


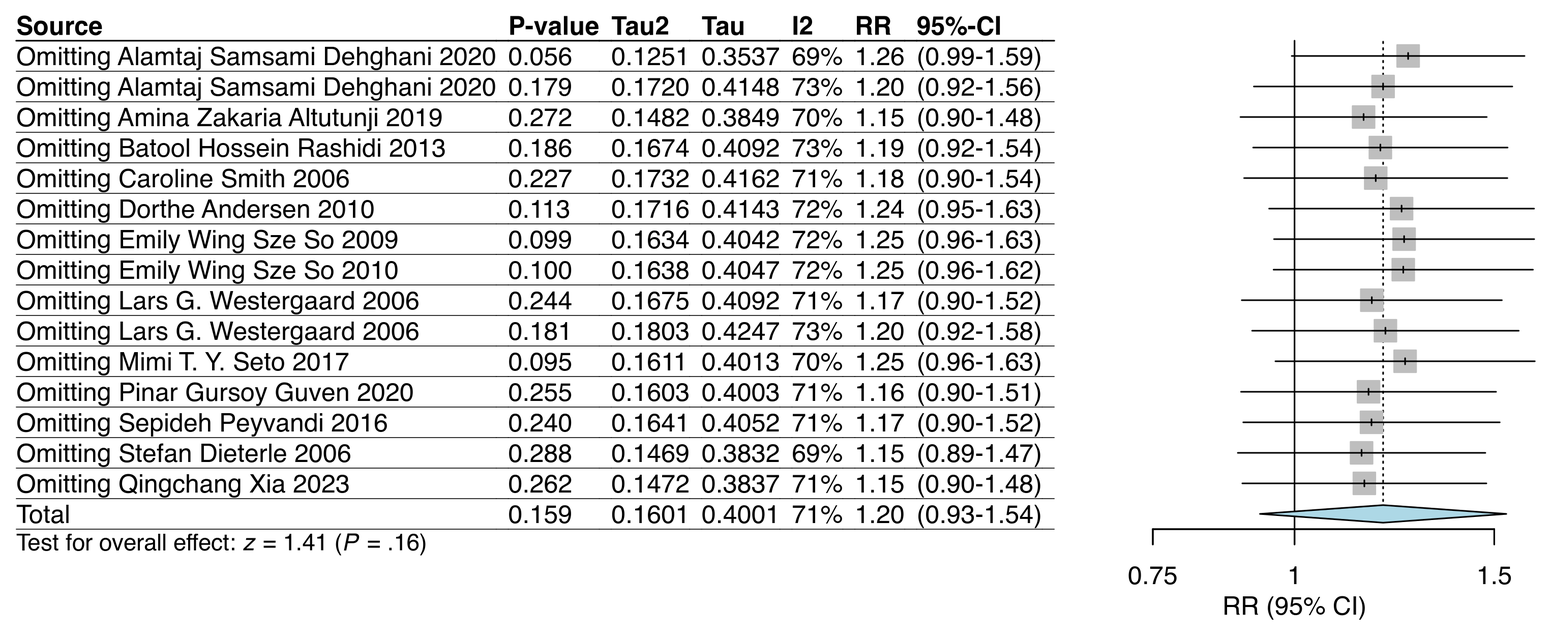


Supplemental Figure 29. Sensitivity analysis for BPR (biochemical pregnancy rate).


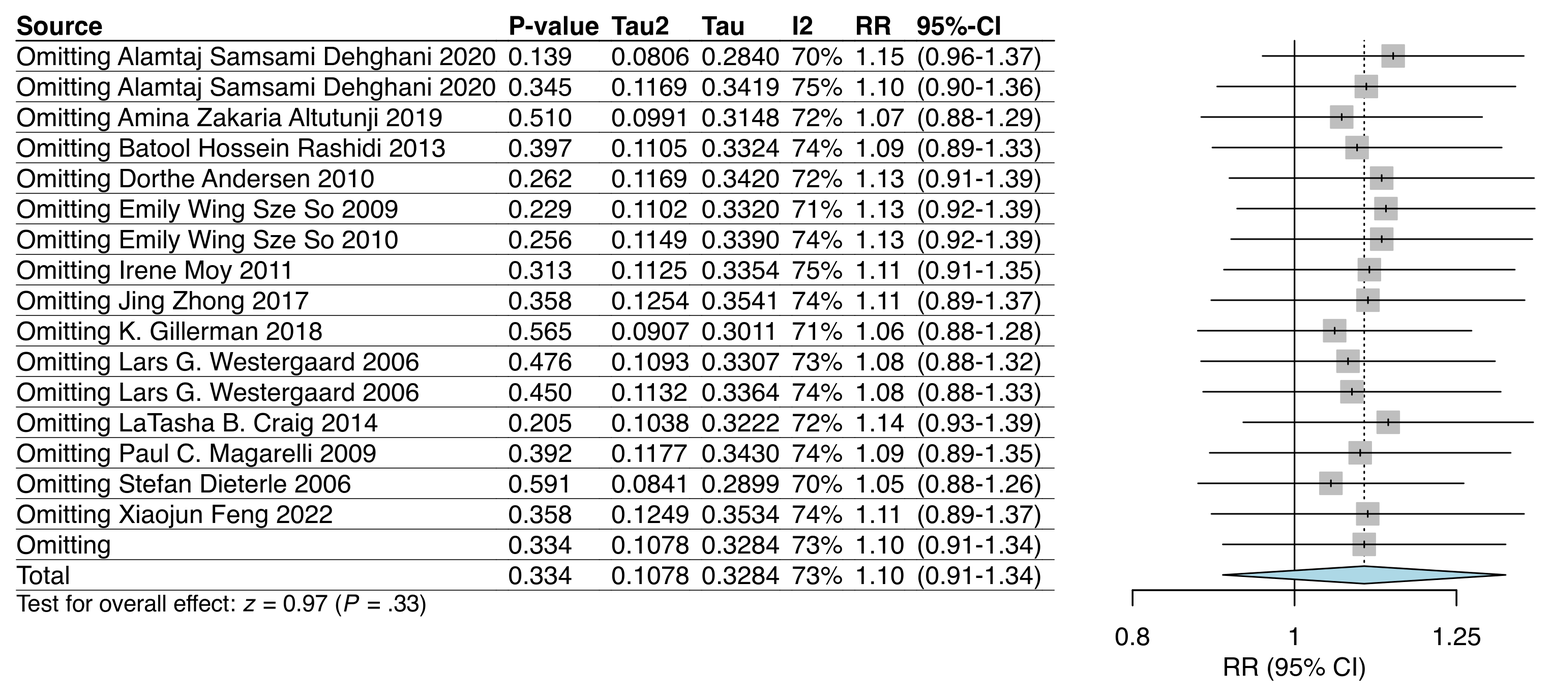


Supplemental Figure 30. Sensitivity analysis for IR (implantation rate).


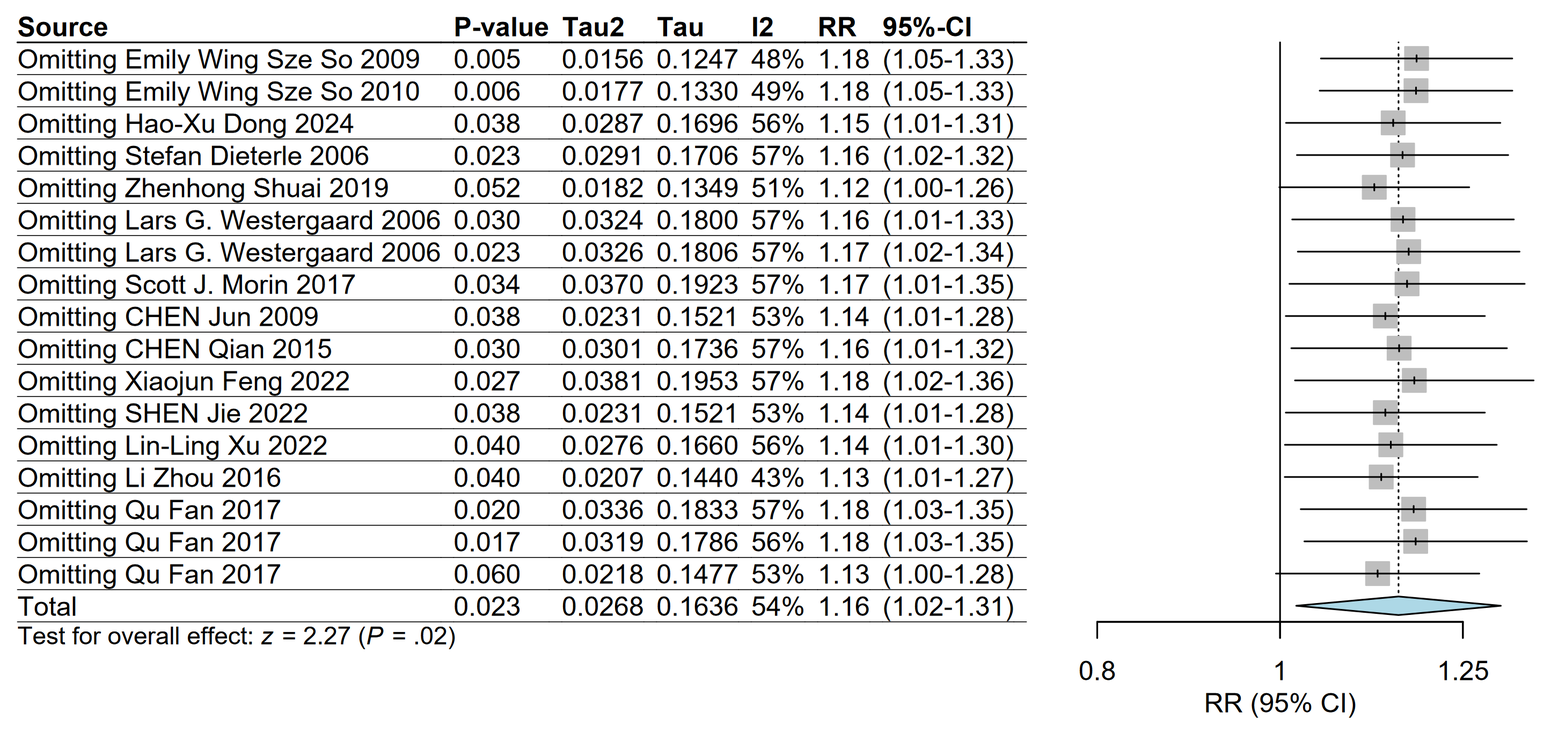


Supplemental Figure 31. Sensitivity analysis for MR (miscarriage rate).


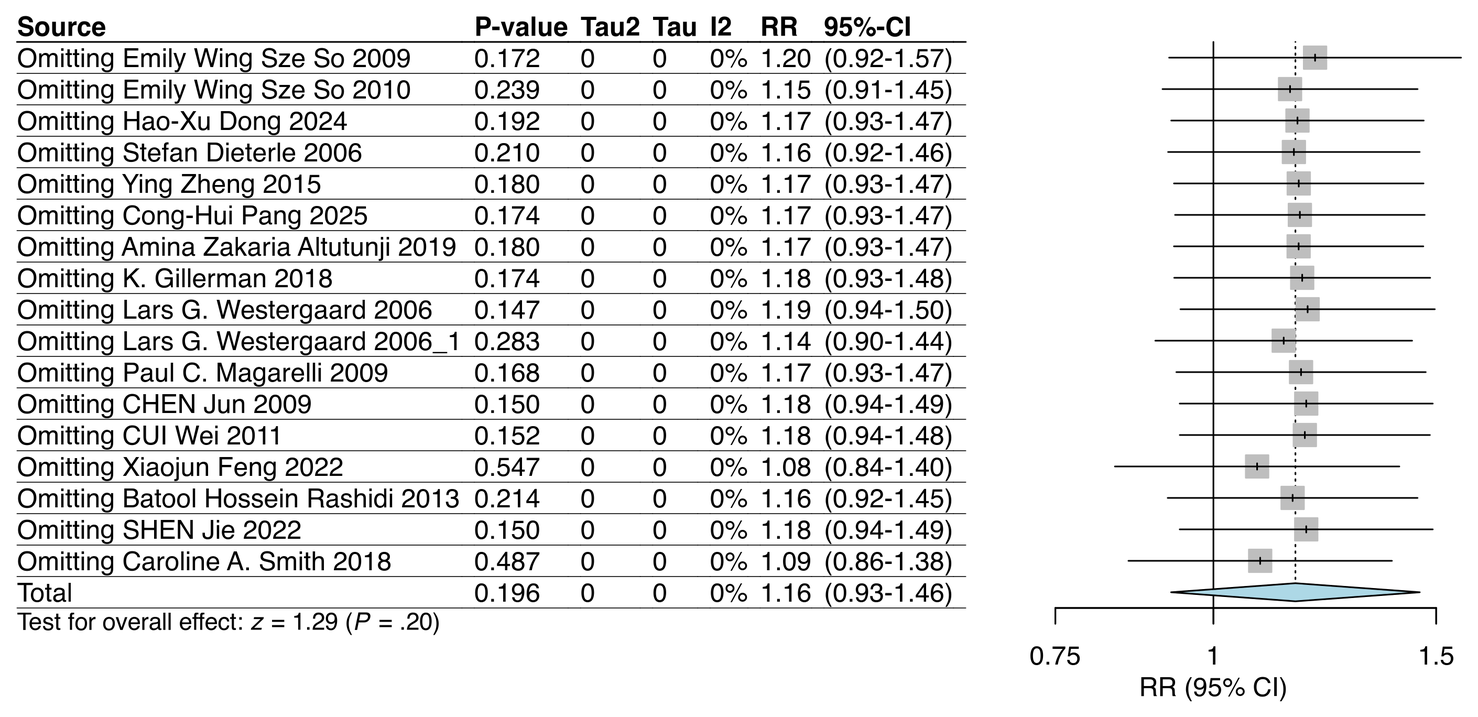


1. **Supplemental File 15. Connectivity plots for network meta-analysis by acupuncture type, needle retention time, and acupuncture timing node.**

Supplemental Figure 32. Connectivity plots for CPR (clinical pregnancy rate). A: acupuncture type; B: needle retention time; C: acupuncture timing node.


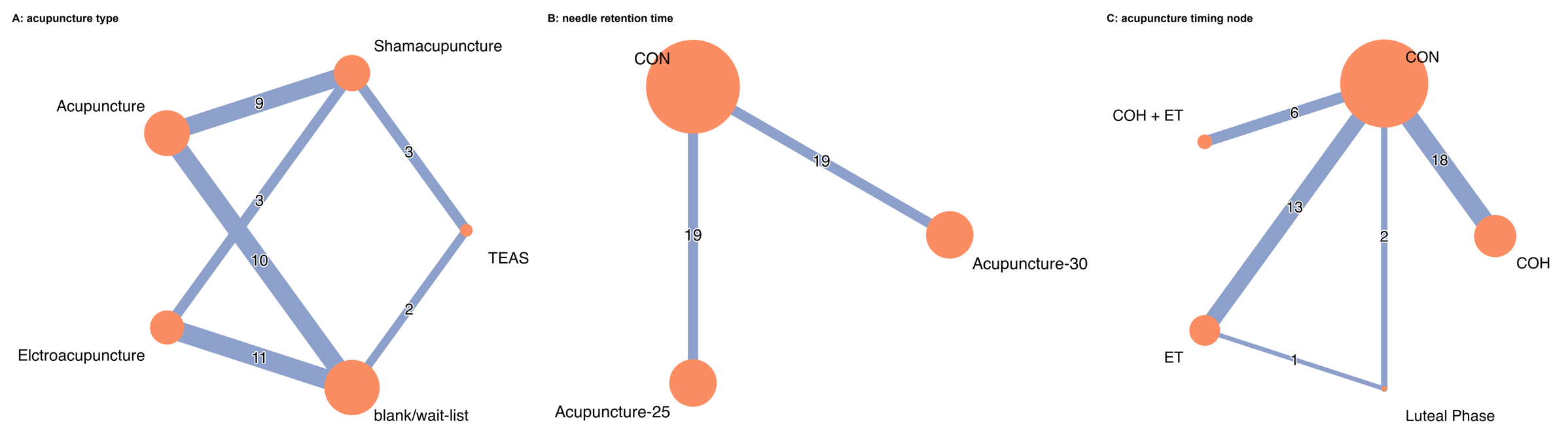


Supplemental Figure 33. Connectivity plots for LBR (live birth rate). A: acupuncture type; B: needle retention time; C: acupuncture timing node.


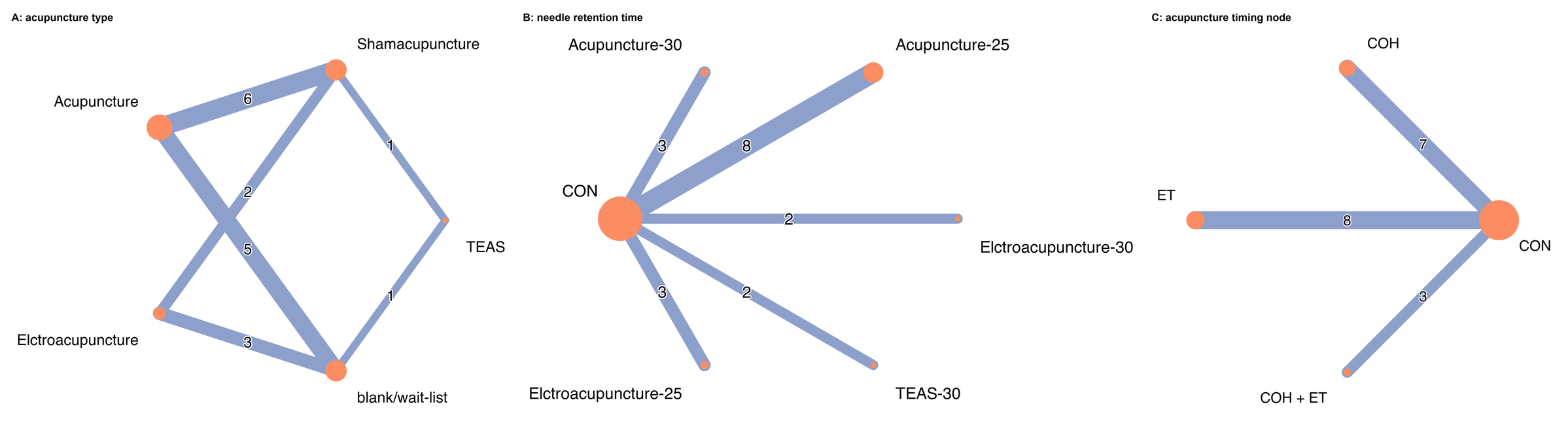


Supplemental Figure 34. Connectivity plots for OPR (ongoing pregnancy rate). A: acupuncture type; B: needle retention time; C: acupuncture timing node.


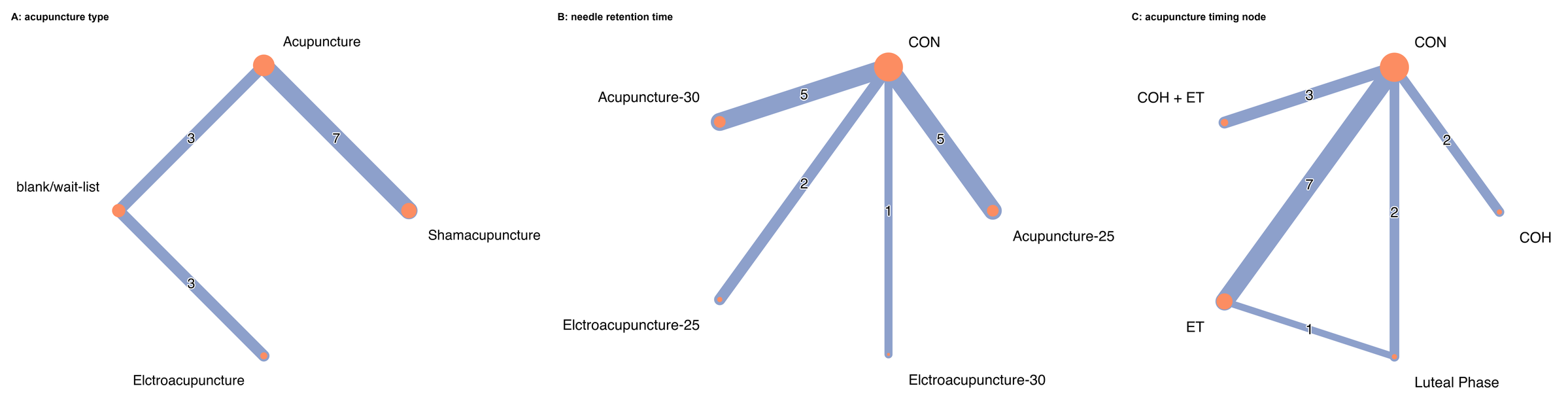


Supplemental Figure 35. Connectivity plots for BPR (biochemical pregnancy rate). A: acupuncture type; B: needle retention time; C: acupuncture timing node.


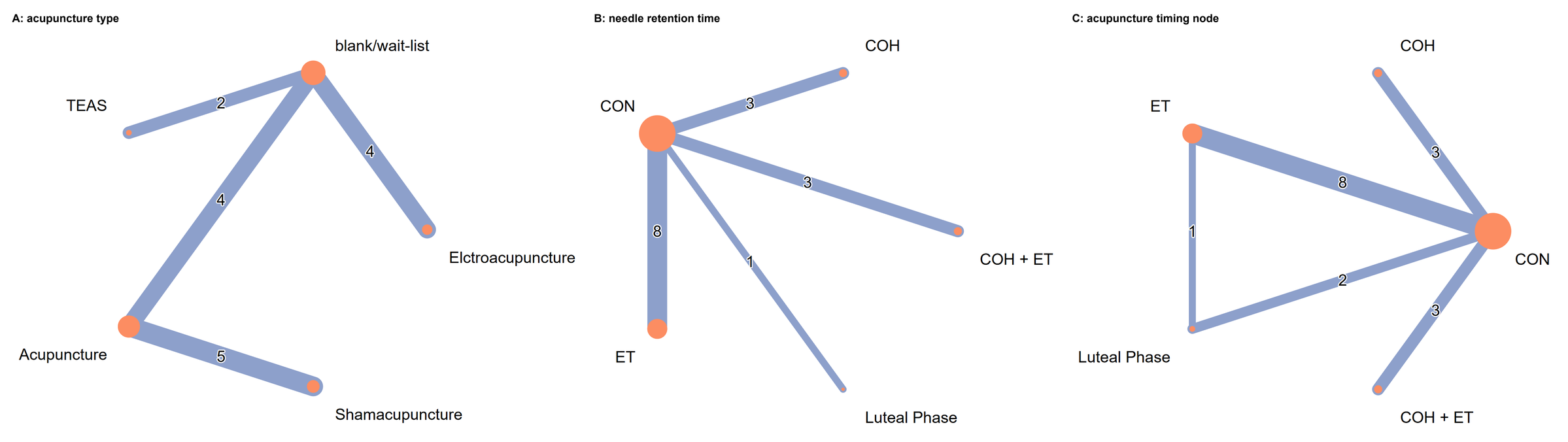


Supplemental Figure 36. Connectivity plots for IR (implantation rate). A: acupuncture type; B: needle retention time; C: acupuncture timing node.


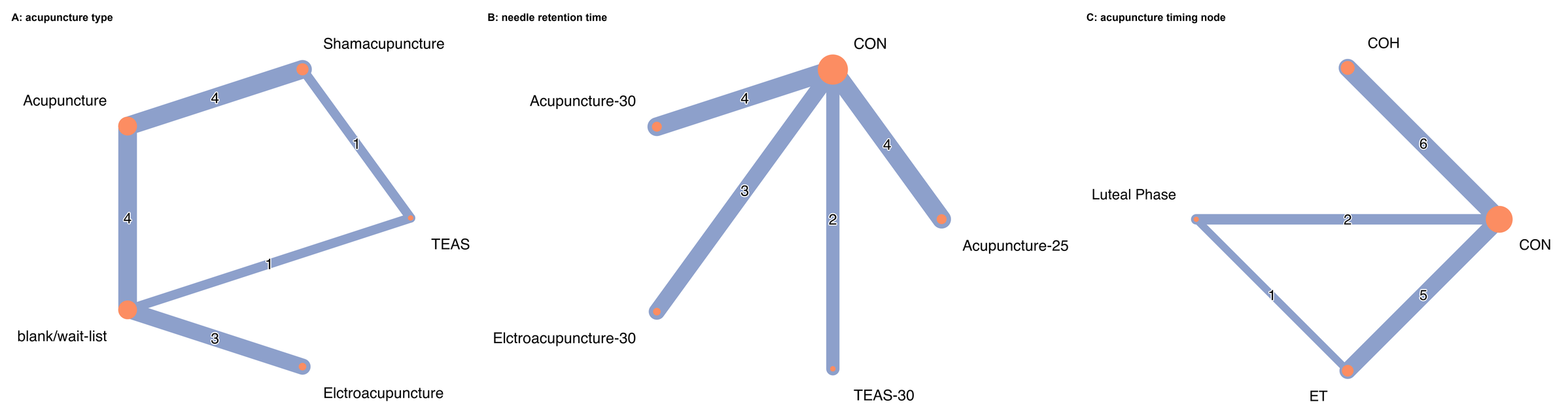


Supplemental Figure 37. Connectivity plots for MR (miscarriage rate). A: acupuncture type; B: needle retention time; C: acupuncture timing node.


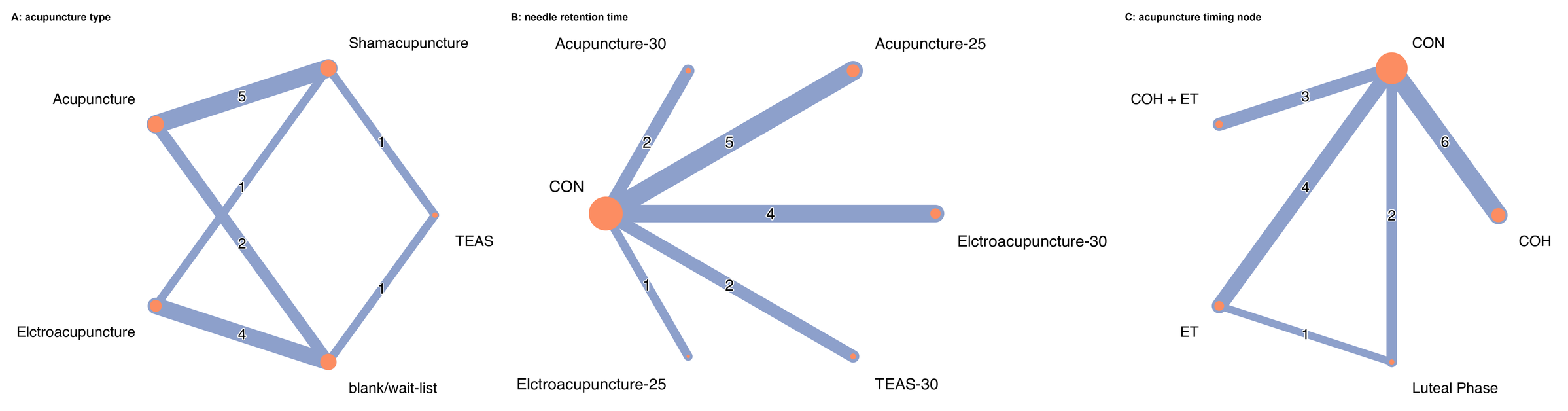


1. **Supplemental File 16. Consistency assessment for network meta-analysis by acupuncture type, needle retention time, and acupuncture timing node.**

Supplemental Figure 38. Consistency assessment plots for OPR (ongoing pregnancy rate). A: acupuncture type; B: needle retention time; C: acupuncture timing node.


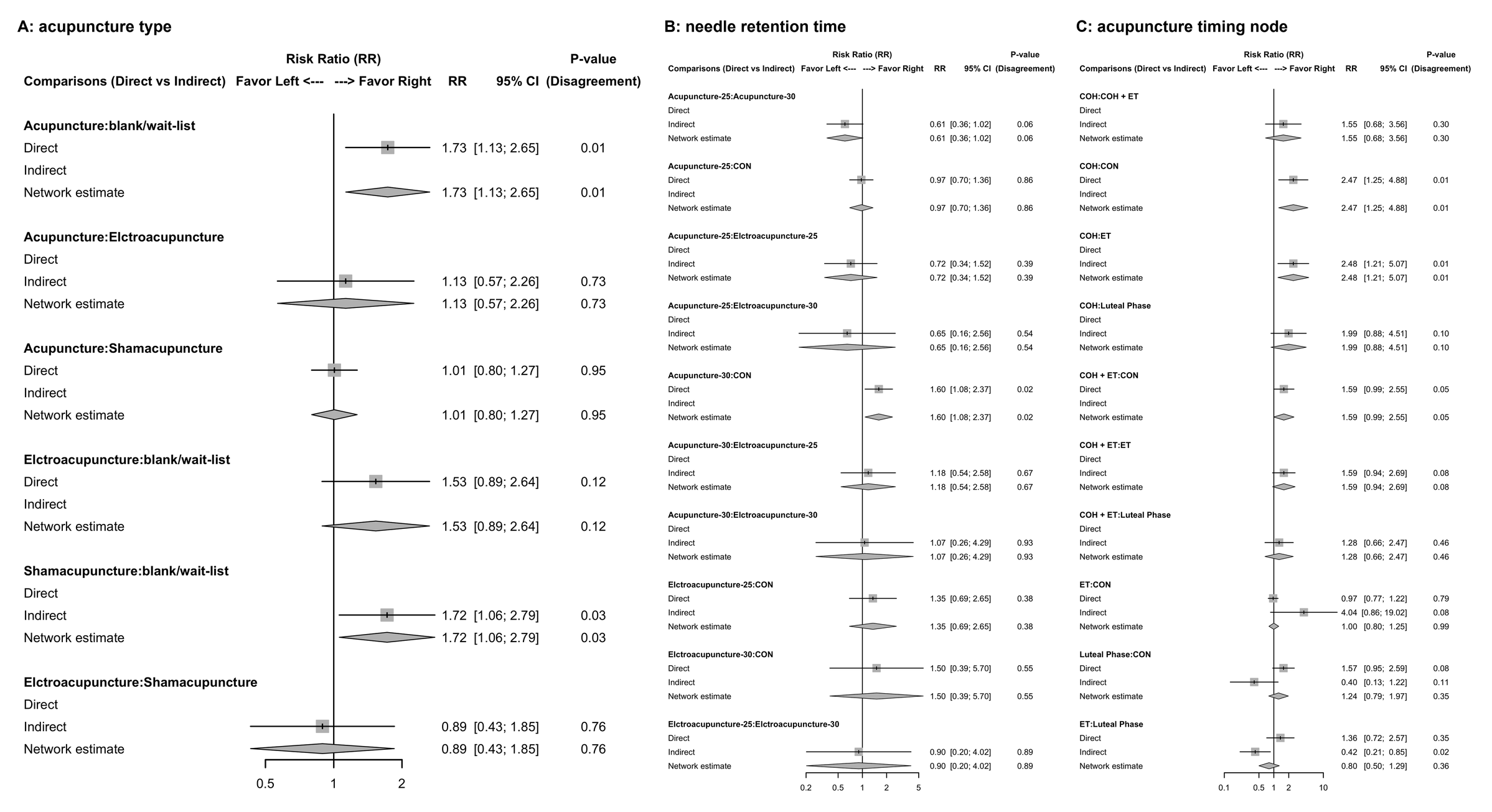


Supplemental Figure 39. Consistency assessment plots for BPR (biochemical pregnancy rate). A: acupuncture type; B: needle retention time; C: acupuncture timing node.


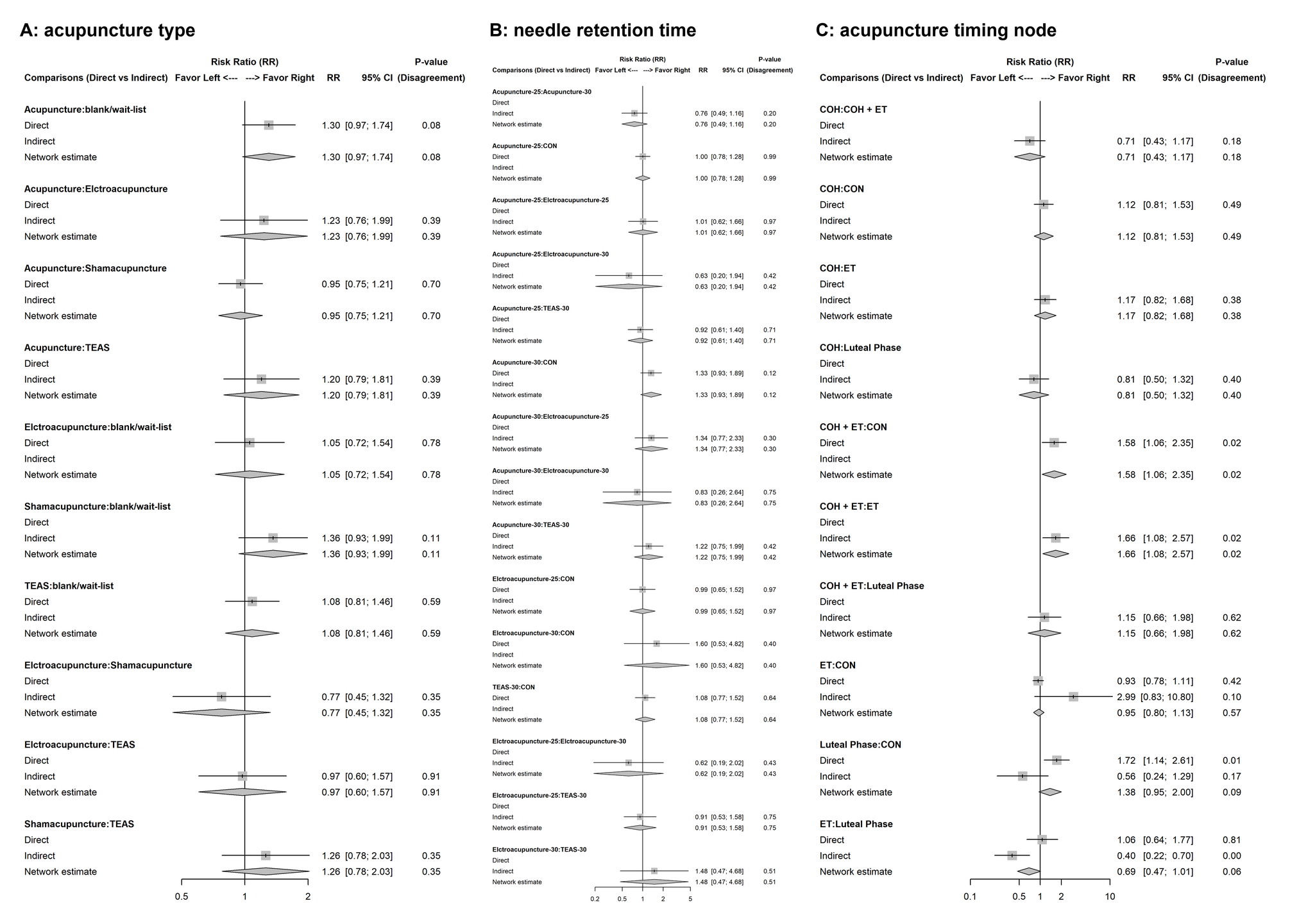


Supplemental Figure 40. Consistency assessment plots for IR (implantation rate). A: acupuncture type; B: needle retention time; C: acupuncture timing node.


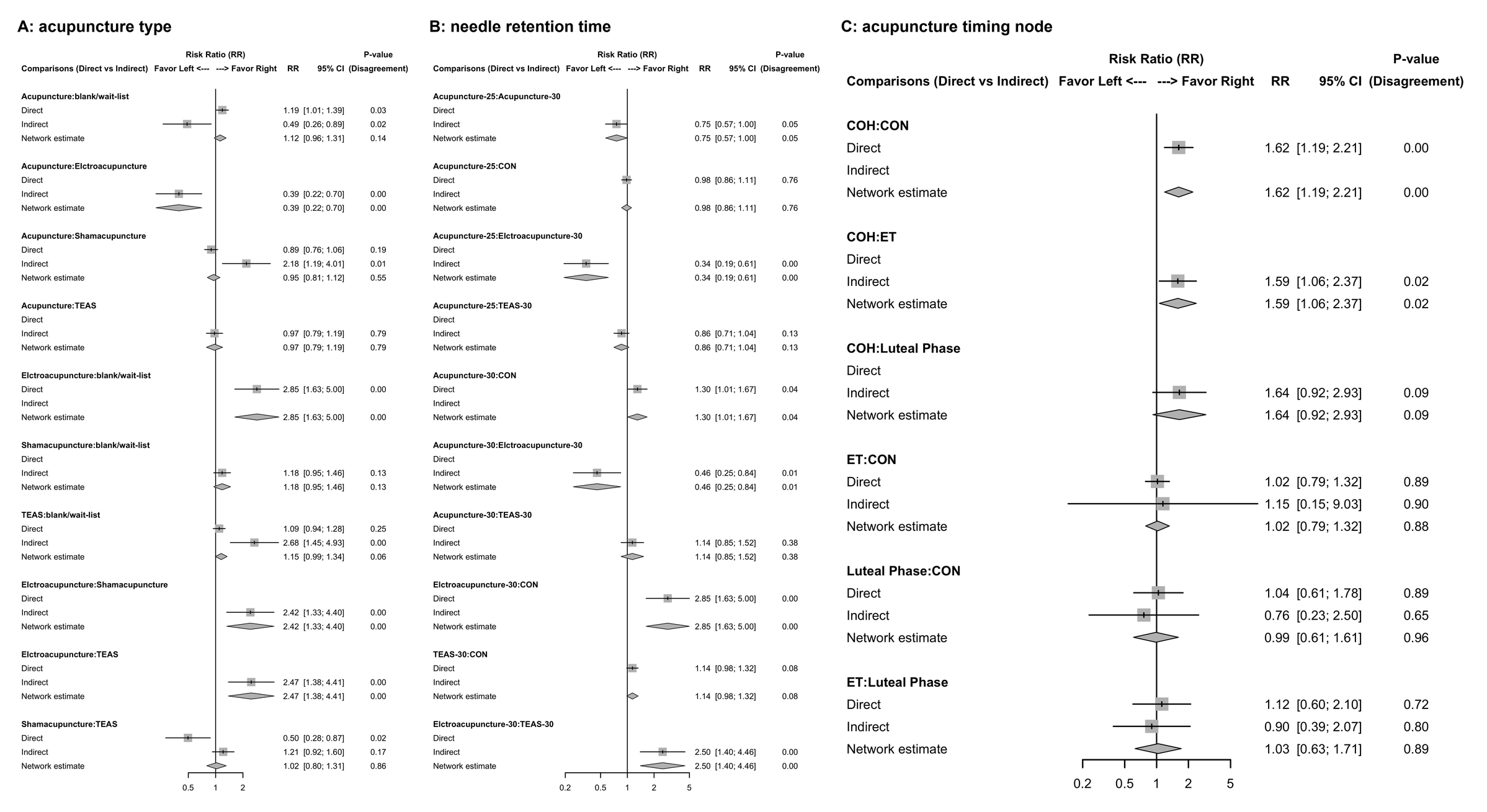


Supplemental Figure 41. Consistency assessment plots for MR (miscarriage rate). A: acupuncture type; B: needle retention time; C: acupuncture timing node.


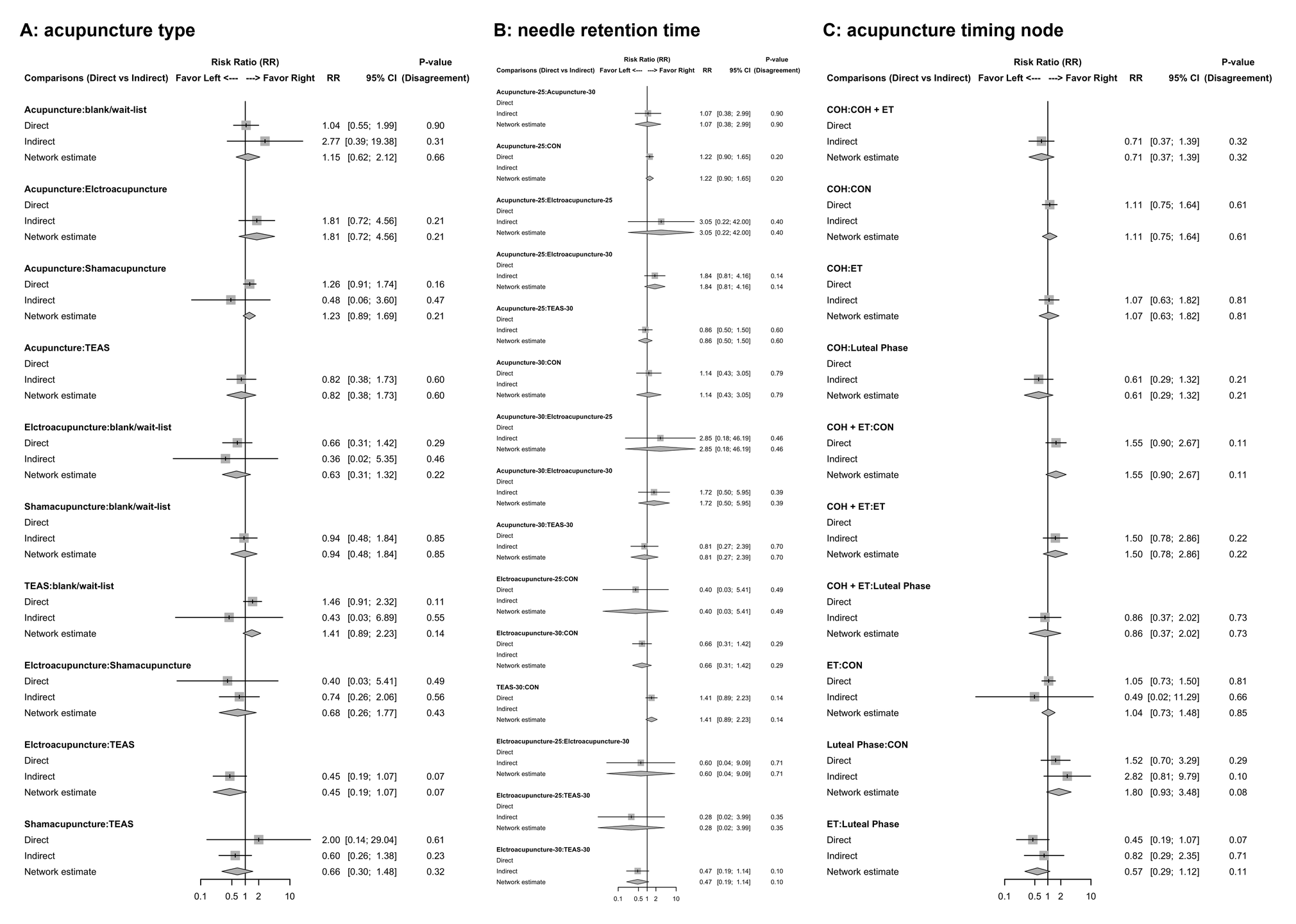


1. **Supplemental File 17. Transitivity assumption assessment for network meta-analysis by acupuncture type, needle retention time, and acupuncture timing node.**

Supplemental Figure 42. Transitivity assumption assessment plots for CPR (clinical pregnancy rate). A: acupuncture type; B: needle retention time; C: acupuncture timing node.


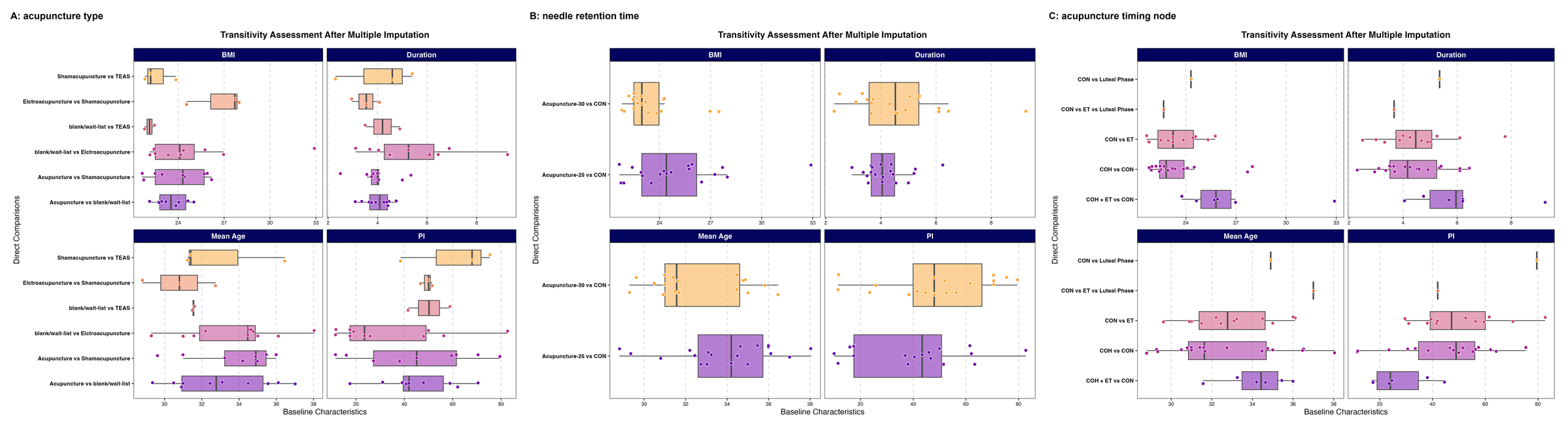


Supplemental Figure 43. Transitivity assumption assessment plots for LBR (live birth rate). A: acupuncture type; B: needle retention time; C: acupuncture timing node.


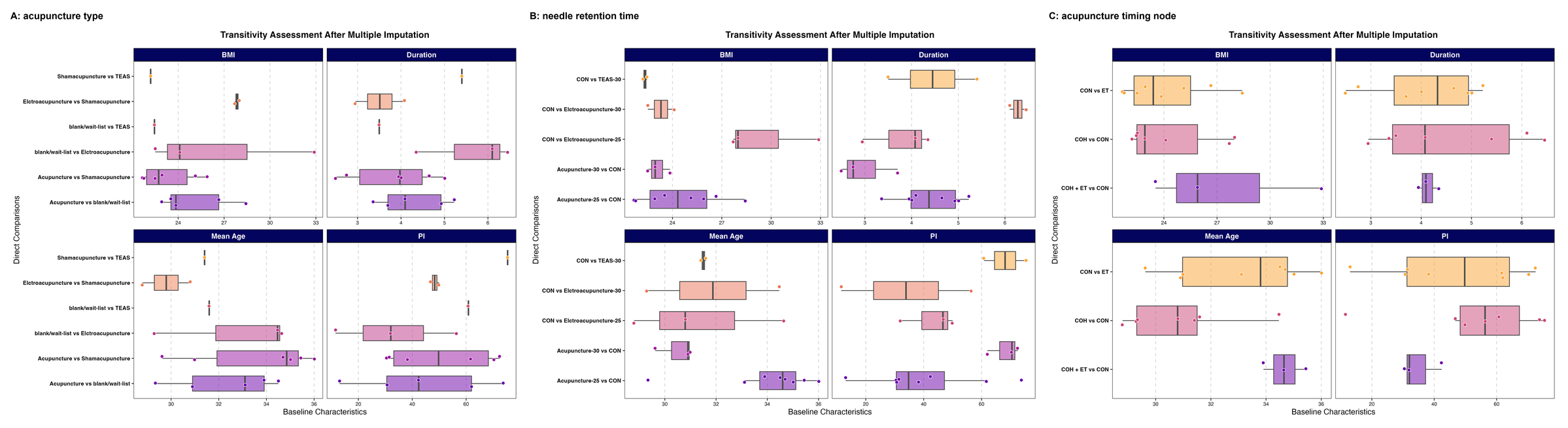


Supplemental Figure 44. Transitivity assumption assessment plots for OPR (ongoing pregnancy rate). A: acupuncture type; B: needle retention time; C: acupuncture timing node.


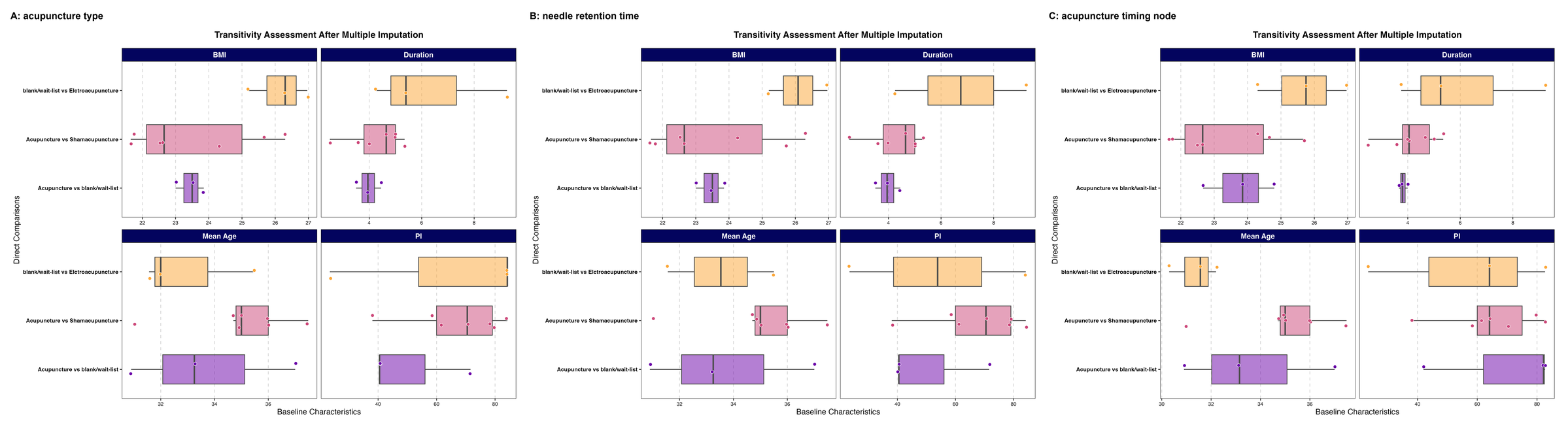


Supplemental Figure 45. Transitivity assumption assessment plots for BPR (biochemical pregnancy rate). A: acupuncture type; B: needle retention time; C: acupuncture timing node.


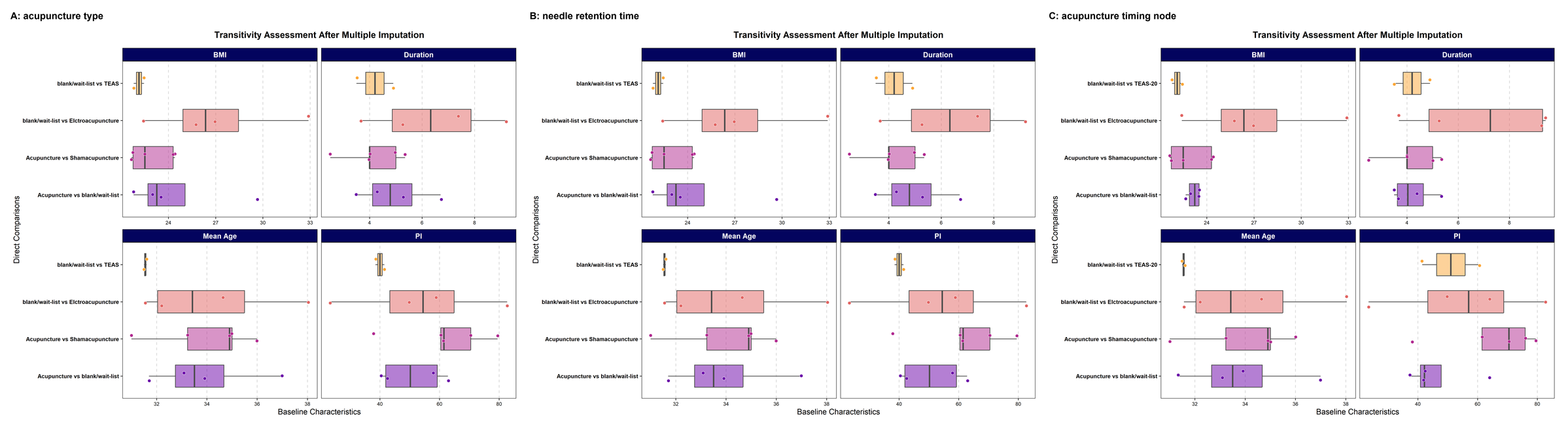


Supplemental Figure 46. Transitivity assumption assessment plots for IR (implantation rate). A: acupuncture type; B: needle retention time; C: acupuncture timing node.


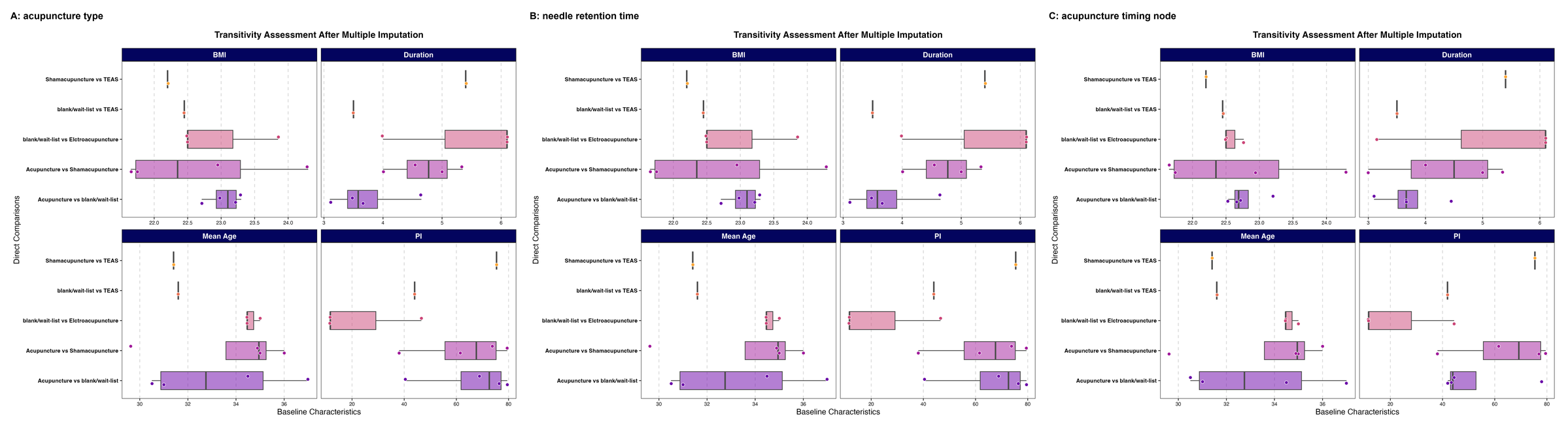


Supplemental Figure 47. Transitivity assumption assessment plots for MR (miscarriage rate). A: acupuncture type; B: needle retention time; C: acupuncture timing node.


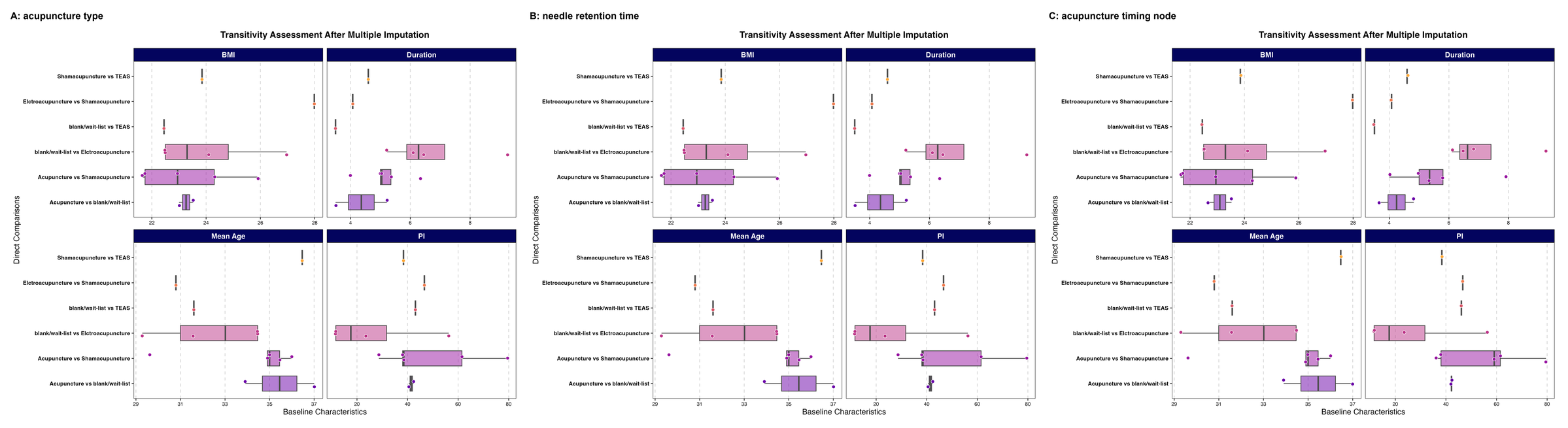


Additional transitivity assessment based on the distribution of clinical and methodological effect modifiers across intervention timing nodes is provided below.

**Supplemental Table 11. Distribution of potential effect modifiers across intervention timing nodes.**

| **Timing node** | **No. of studies** | **Infertility diagnosis / diagnostic restriction** | **Transfer type** | **Acupuncture modality** | **Sessions / treatment intensity** | **Comparator class** | **Implication for transitivity** |
| --- | --- | --- | --- | --- | --- | --- | --- |
| COH | 19 | PCOS: 6/19 (31.6%); unselected infertility population: 6/19 (31.6%); poor ovarian response: 2/19 (10.5%); advanced reproductive age: 2/19 (10.5%); diminished ovarian reserve: 2/19 (10.5%); recurrent implantation failure: 1/19 (5.3%) | Fresh embryo transfer: 12/19 (63.2%); Fresh and/or frozen embryo transfer: 5/19 (26.3%); Frozen-thawed embryo transfer: 2/19 (10.5%) | Manual acupuncture: 5/19 (26.3%); Electroacupuncture: 10/19 (52.6%); TEAS: 4/19 (21.1%) | median 5.5 sessions (range 2-10); NR: 9/19 | inactive control: 12/19 (63.2%); nonpenetrating sham acupuncture: 4/19 (21.1%); sham TEAS: 3/19 (15.8%) | Potential transitivity concern due to mixed embryo-transfer settings, heterogeneous comparator designs, wide treatment-intensity range; indirect estimates involving this node should be interpreted cautiously. |
| ET | 13 | unselected infertility population: 13/13 (100.0%) | Fresh embryo transfer: 9/13 (69.2%); Fresh and/or frozen embryo transfer: 4/13 (30.8%) | Manual acupuncture: 11/13 (84.6%); TEAS: 2/13 (15.4%) | median 2 sessions (range 1-4); NR: 1/13 | inactive control: 8/13 (61.5%); nonpenetrating sham acupuncture: 3/13 (23.1%); superficial sham acupuncture: 2/13 (15.4%) | Potential transitivity concern due to mixed embryo-transfer settings, heterogeneous comparator designs; indirect estimates involving this node should be interpreted cautiously. |
| COH + ET | 7 | unselected infertility population: 6/7 (85.7%); PCOS: 1/7 (14.3%) | Fresh embryo transfer: 4/7 (57.1%); Fresh and/or frozen embryo transfer: 3/7 (42.9%) | Manual acupuncture: 5/7 (71.4%); Electroacupuncture: 2/7 (28.6%) | median 3 sessions (range 3-10); NR: 0/7 | inactive control: 4/7 (57.1%); nonpenetrating sham acupuncture: 2/7 (28.6%); superficial sham acupuncture: 1/7 (14.3%) | Potential transitivity concern due to mixed embryo-transfer settings, heterogeneous comparator designs, wide treatment-intensity range; indirect estimates involving this node should be interpreted cautiously. |
| Luteal phase | 3 | unselected infertility population: 3/3 (100.0%) | Fresh embryo transfer: 1/3 (33.3%); Fresh and/or frozen embryo transfer: 1/3 (33.3%); Frozen-thawed embryo transfer: 1/3 (33.3%) | Manual acupuncture: 3/3 (100.0%) | median 1.5 sessions (range 1-2); NR: 1/3 | inactive control: 1/3 (33.3%); nonpenetrating sham acupuncture: 1/3 (33.3%); superficial sham acupuncture: 1/3 (33.3%) | Potential transitivity concern due to sparse evidence, mixed embryo-transfer settings, heterogeneous comparator designs; indirect estimates involving this node should be interpreted cautiously. |

*Note. Values are summarized at the study level. Percentages are calculated within each intervention timing node. NR indicates not reported. Laser acupuncture was reclassified as manual acupuncture according to author clarification. The table is intended to support clinical assessment of the transitivity assumption rather than to prove transitivity statistically.*

**Supplemental Table 12. Long-format distribution of potential effect modifiers by intervention timing node.**

| **Effect modifier** | **Timing node** | **Category** | **Studies** | **Denominator** | **Percent** |
| --- | --- | --- | --- | --- | --- |
| Infertility diagnosis / diagnostic restriction | COH | PCOS | 6 | 19 | 31.6 |
| Infertility diagnosis / diagnostic restriction | COH | unselected infertility population | 6 | 19 | 31.6 |
| Infertility diagnosis / diagnostic restriction | COH | poor ovarian response | 2 | 19 | 10.5 |
| Infertility diagnosis / diagnostic restriction | COH | advanced reproductive age | 2 | 19 | 10.5 |
| Infertility diagnosis / diagnostic restriction | COH | diminished ovarian reserve | 2 | 19 | 10.5 |
| Infertility diagnosis / diagnostic restriction | COH | recurrent implantation failure | 1 | 19 | 5.3 |
| Infertility diagnosis / diagnostic restriction | ET | unselected infertility population | 13 | 13 | 100 |
| Infertility diagnosis / diagnostic restriction | COH + ET | unselected infertility population | 6 | 7 | 85.7 |
| Infertility diagnosis / diagnostic restriction | COH + ET | PCOS | 1 | 7 | 14.3 |
| Infertility diagnosis / diagnostic restriction | Luteal phase | unselected infertility population | 3 | 3 | 100 |
| Transfer type | COH | Fresh embryo transfer | 12 | 19 | 63.2 |
| Transfer type | COH | Fresh and/or frozen embryo transfer | 5 | 19 | 26.3 |
| Transfer type | COH | Frozen-thawed embryo transfer | 2 | 19 | 10.5 |
| Transfer type | ET | Fresh embryo transfer | 9 | 13 | 69.2 |
| Transfer type | ET | Fresh and/or frozen embryo transfer | 4 | 13 | 30.8 |
| Transfer type | COH + ET | Fresh embryo transfer | 4 | 7 | 57.1 |
| Transfer type | COH + ET | Fresh and/or frozen embryo transfer | 3 | 7 | 42.9 |
| Transfer type | Luteal phase | Fresh and/or frozen embryo transfer | 1 | 3 | 33.3 |
| Transfer type | Luteal phase | Frozen-thawed embryo transfer | 1 | 3 | 33.3 |
| Transfer type | Luteal phase | Fresh embryo transfer | 1 | 3 | 33.3 |
| Acupuncture modality | COH | Electroacupuncture | 10 | 19 | 52.6 |
| Acupuncture modality | COH | Manual acupuncture | 5 | 19 | 26.3 |
| Acupuncture modality | COH | TEAS | 4 | 19 | 21.1 |
| Acupuncture modality | ET | Manual acupuncture | 11 | 13 | 84.6 |
| Acupuncture modality | ET | TEAS | 2 | 13 | 15.4 |
| Acupuncture modality | COH + ET | Manual acupuncture | 5 | 7 | 71.4 |
| Acupuncture modality | COH + ET | Electroacupuncture | 2 | 7 | 28.6 |
| Acupuncture modality | Luteal phase | Manual acupuncture | 3 | 3 | 100 |
| Comparator class | COH | inactive control | 12 | 19 | 63.2 |
| Comparator class | COH | nonpenetrating sham acupuncture | 4 | 19 | 21.1 |
| Comparator class | COH | sham TEAS | 3 | 19 | 15.8 |
| Comparator class | ET | inactive control | 8 | 13 | 61.5 |
| Comparator class | ET | nonpenetrating sham acupuncture | 3 | 13 | 23.1 |
| Comparator class | ET | superficial sham acupuncture | 2 | 13 | 15.4 |
| Comparator class | COH + ET | inactive control | 4 | 7 | 57.1 |
| Comparator class | COH + ET | nonpenetrating sham acupuncture | 2 | 7 | 28.6 |
| Comparator class | COH + ET | superficial sham acupuncture | 1 | 7 | 14.3 |
| Comparator class | Luteal phase | superficial sham acupuncture | 1 | 3 | 33.3 |
| Comparator class | Luteal phase | nonpenetrating sham acupuncture | 1 | 3 | 33.3 |
| Comparator class | Luteal phase | inactive control | 1 | 3 | 33.3 |
| Treatment intensity group | COH | NR | 9 | 19 | 47.4 |
| Treatment intensity group | COH | Higher (>5 sessions) | 5 | 19 | 26.3 |
| Treatment intensity group | COH | Moderate (3-5 sessions) | 4 | 19 | 21.1 |
| Treatment intensity group | COH | Low (1-2 sessions) | 1 | 19 | 5.3 |
| Treatment intensity group | ET | Low (1-2 sessions) | 10 | 13 | 76.9 |
| Treatment intensity group | ET | Moderate (3-5 sessions) | 2 | 13 | 15.4 |
| Treatment intensity group | ET | NR | 1 | 13 | 7.7 |
| Treatment intensity group | COH + ET | Moderate (3-5 sessions) | 6 | 7 | 85.7 |
| Treatment intensity group | COH + ET | Higher (>5 sessions) | 1 | 7 | 14.3 |
| Treatment intensity group | Luteal phase | Low (1-2 sessions) | 2 | 3 | 66.7 |
| Treatment intensity group | Luteal phase | NR | 1 | 3 | 33.3 |

*Note. This long-format table provides the numerator, denominator, and percentage for each effect-modifier category within each timing node. It complements Supplemental Table 11 by allowing readers to trace individual distribution components used in the transitivity assessment.*

**Supplemental Figure 48. Distribution of selected clinical and methodological effect modifiers across intervention timing nodes.**


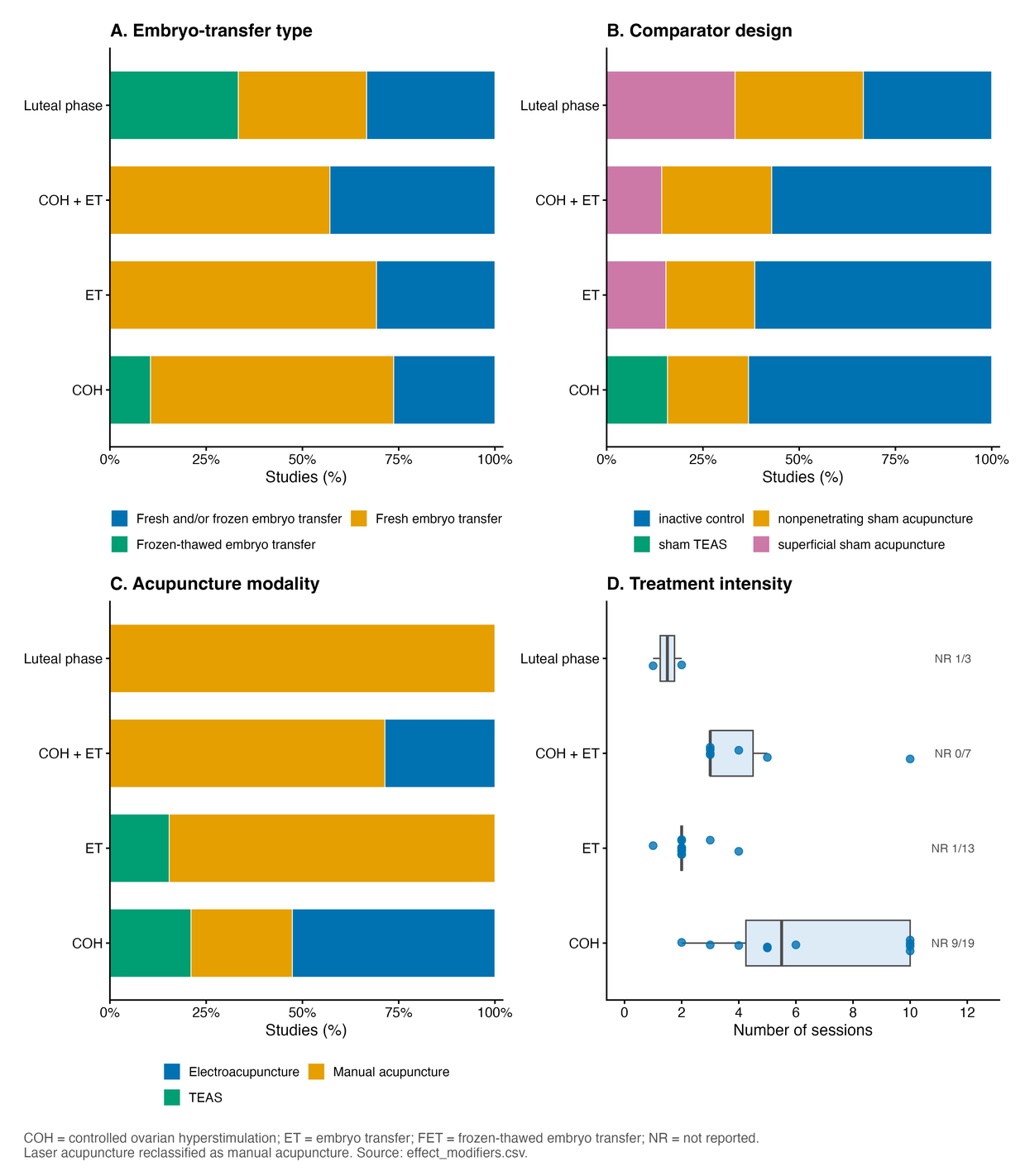


*Note. The figure visually summarizes the distribution of embryo-transfer type, comparator design, acupuncture modality, and treatment intensity across intervention timing nodes. It directly addresses whether important effect modifiers are sufficiently comparable across nodes for indirect comparisons. COH, controlled ovarian hyperstimulation; ET, embryo transfer; FET, frozen-thawed embryo transfer; NR, not reported.*

1. **Supplemental File 18. Funnel plots for network meta-analysis by acupuncture type, needle retention time, and acupuncture timing node.**

Supplemental Figure 49. Funnel plots for CPR (clinical pregnancy rate). A: acupuncture type; B: needle retention time; C: acupuncture timing node.


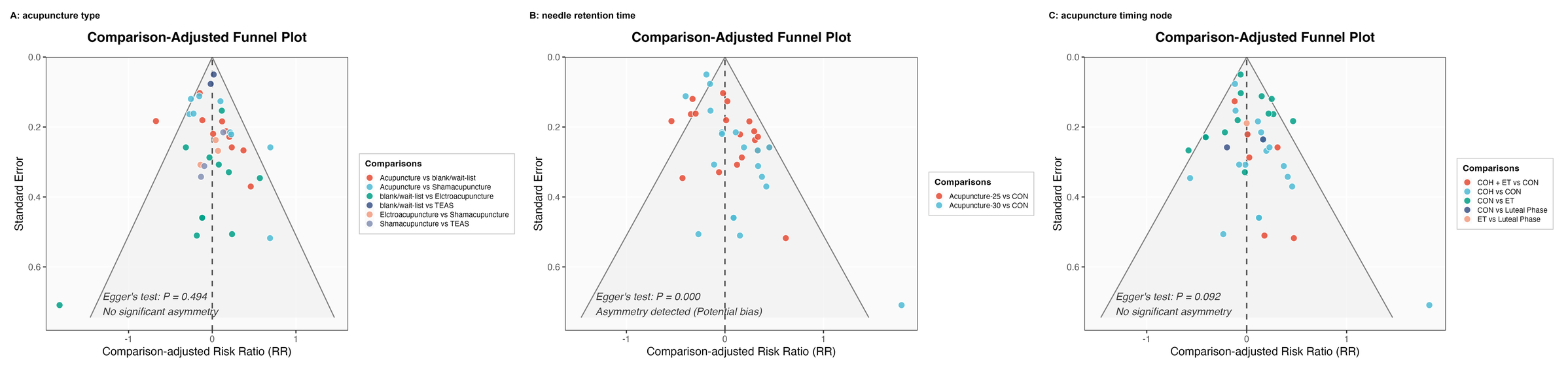


Supplemental Figure 50. Funnel plots for LBR (live birth rate). A: acupuncture type; B: needle retention time; C: acupuncture timing node.


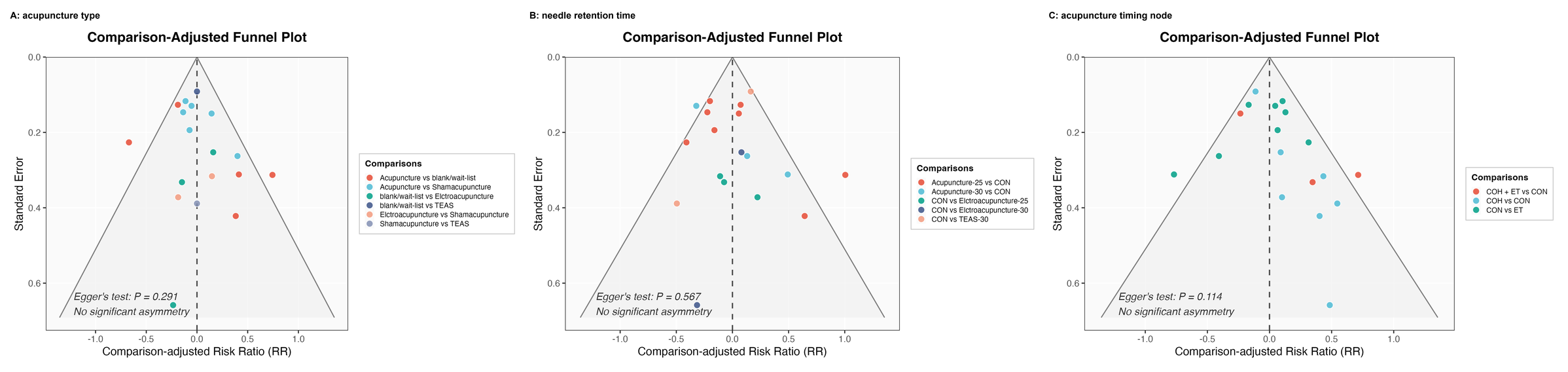


Supplemental Figure 51. Funnel plots for OPR (ongoing pregnancy rate). A: acupuncture type; B: needle retention time; C: acupuncture timing node.


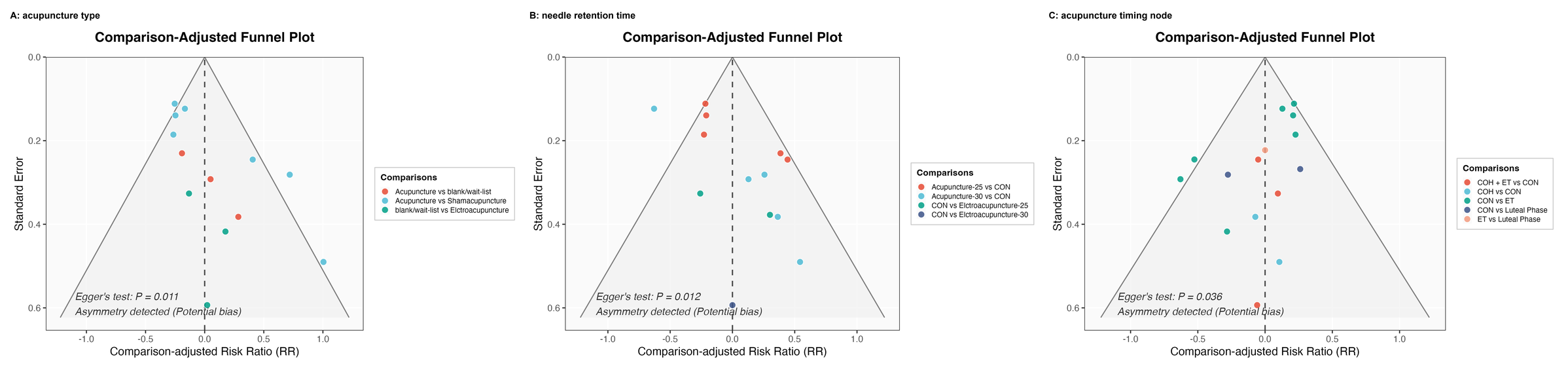


Supplemental Figure 52. Funnel plots for BPR (biochemical pregnancy rate). A: acupuncture type; B: needle retention time; C: acupuncture timing node.


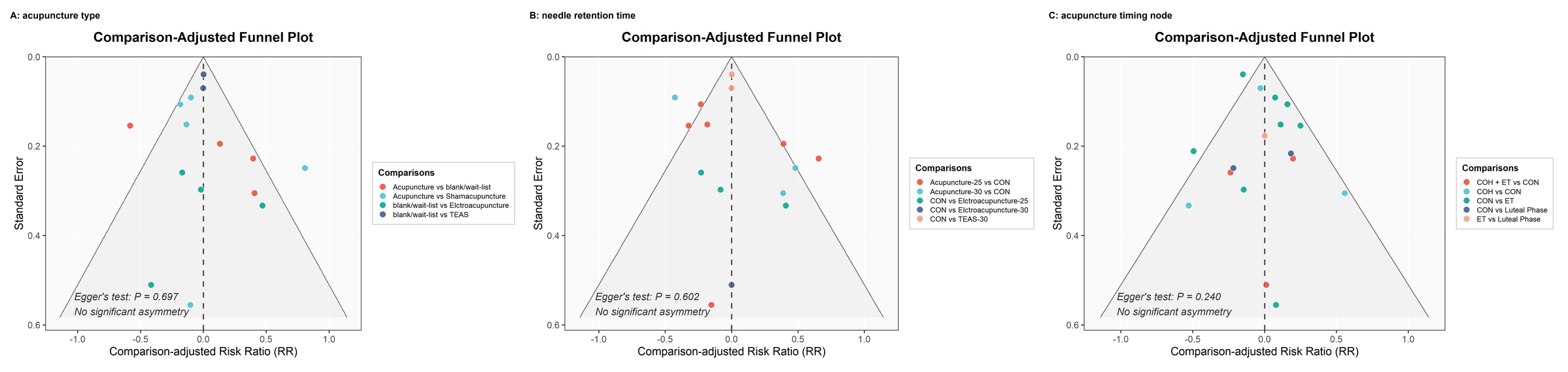


Supplemental Figure 53. Funnel plots for IR (implantation rate). A: acupuncture type; B: needle retention time; C: acupuncture timing node.


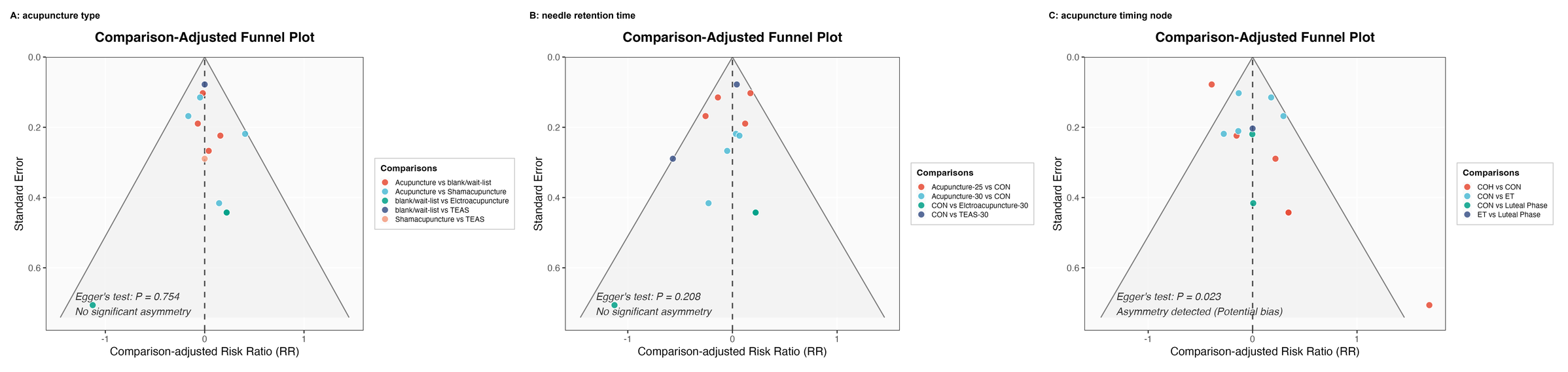


Supplemental Figure 54. Funnel plots for MR (miscarriage rate). A: acupuncture type; B: needle retention time; C: acupuncture timing node.


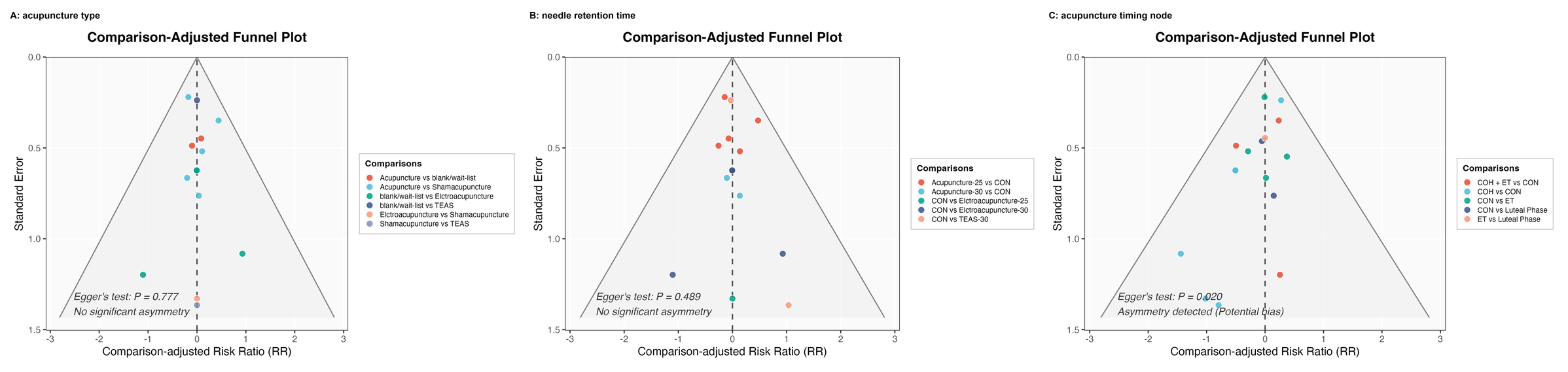


1. **Supplemental File 19. SUCRA ranking plots for network meta-analysis by acupuncture type, needle retention time, and acupuncture timing node.**

Supplemental Figure 55. SUCRA ranking plots for CPR (clinical pregnancy rate). A: acupuncture type; B: needle retention time; C: acupuncture timing node.


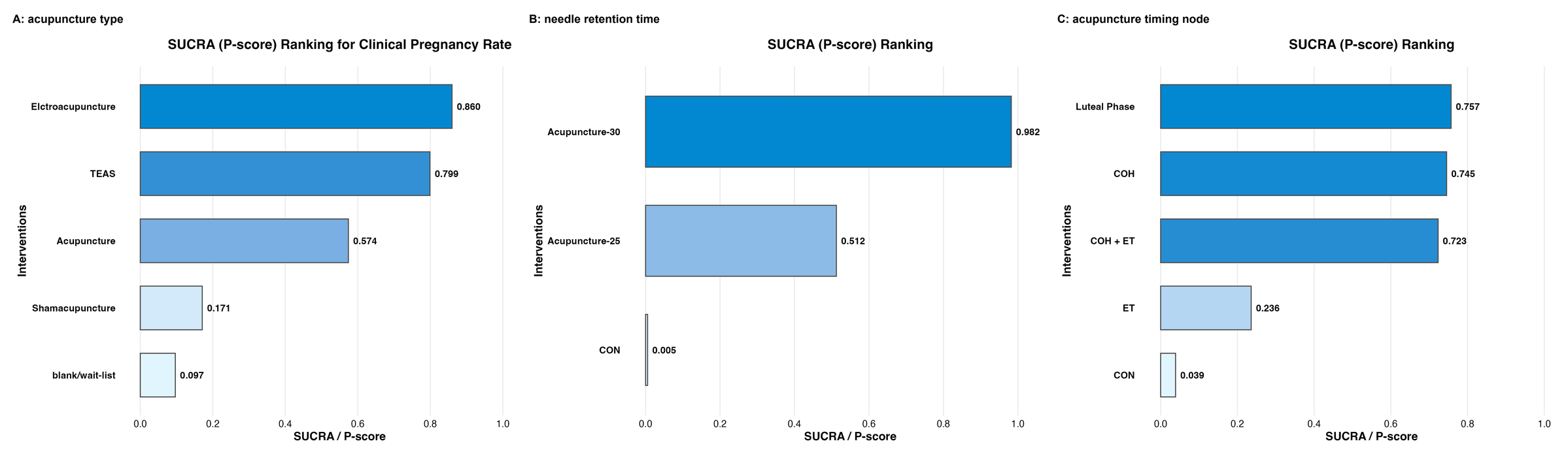


Supplemental Figure 56. SUCRA ranking plots for LBR (live birth rate). A: acupuncture type; B: needle retention time; C: acupuncture timing node.


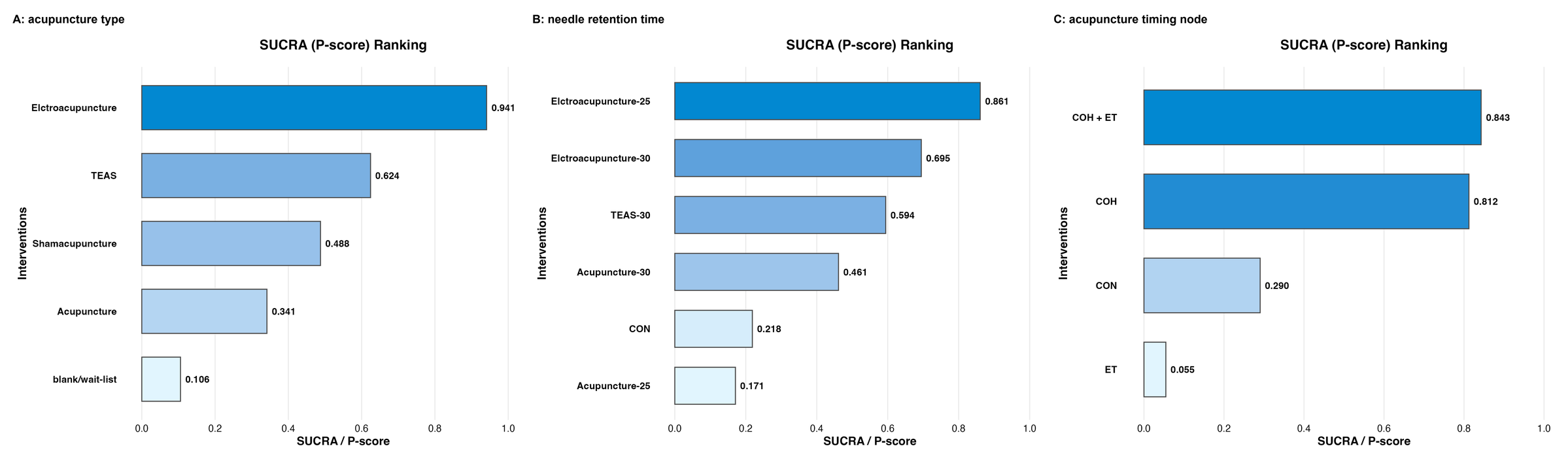


Supplemental Figure 57. SUCRA ranking plots for OPR (ongoing pregnancy rate). A: acupuncture type; B: needle retention time; C: acupuncture timing node.


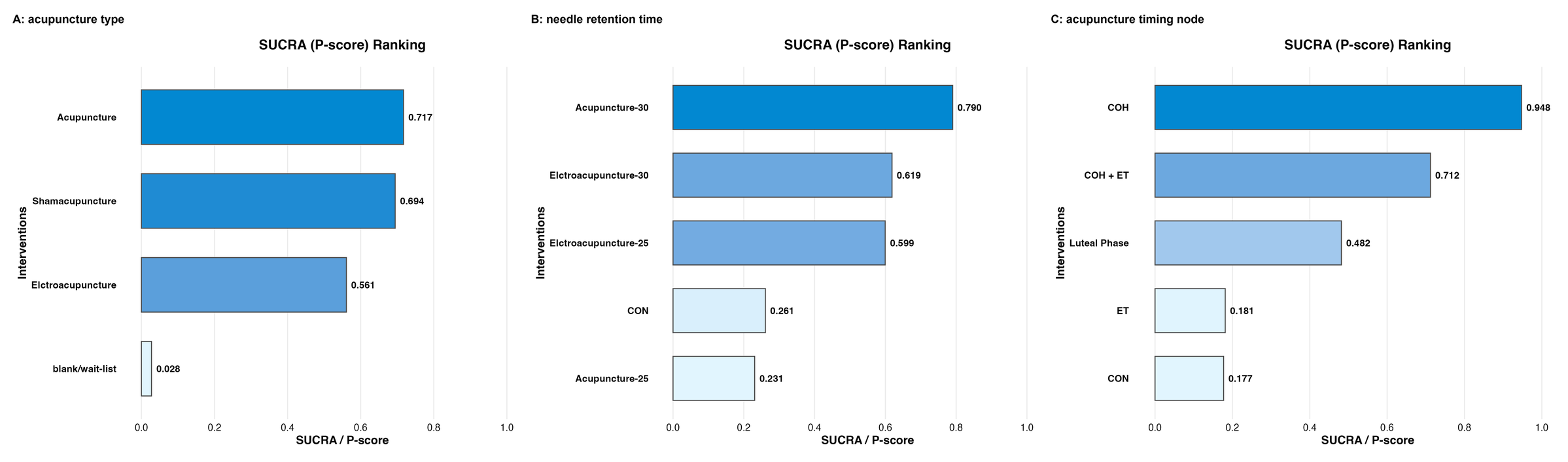


Supplemental Figure 58. SUCRA ranking plots for BPR (biochemical pregnancy rate). A: acupuncture type; B: needle retention time; C: acupuncture timing node.


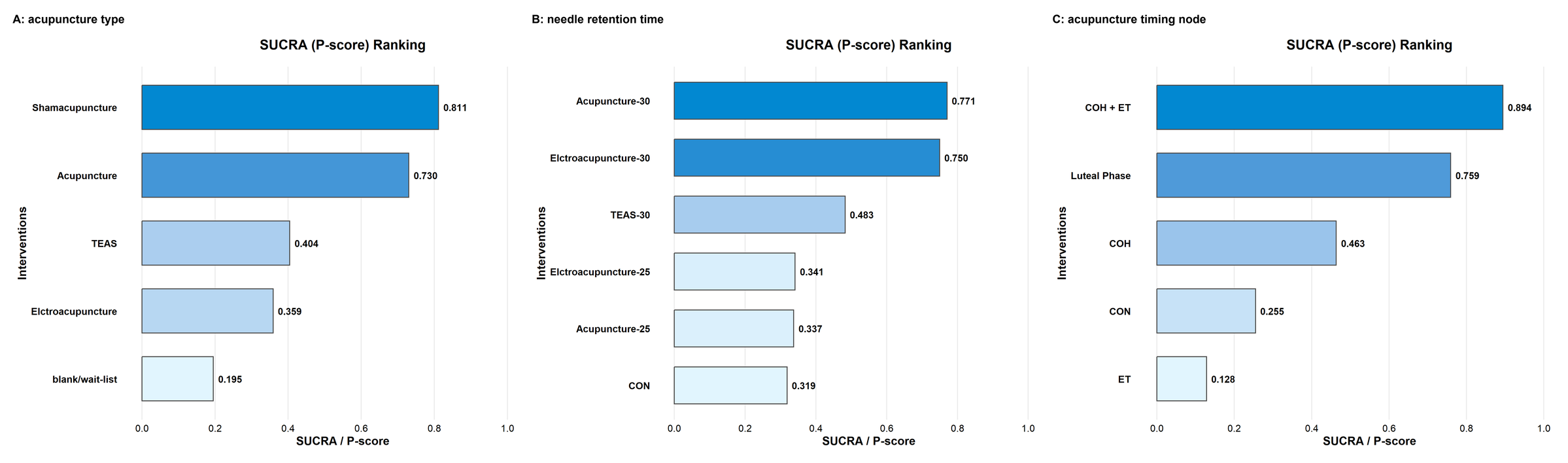


Supplemental Figure 59. SUCRA ranking plots for IR (implantation rate). A: acupuncture type; B: needle retention time; C: acupuncture timing node.


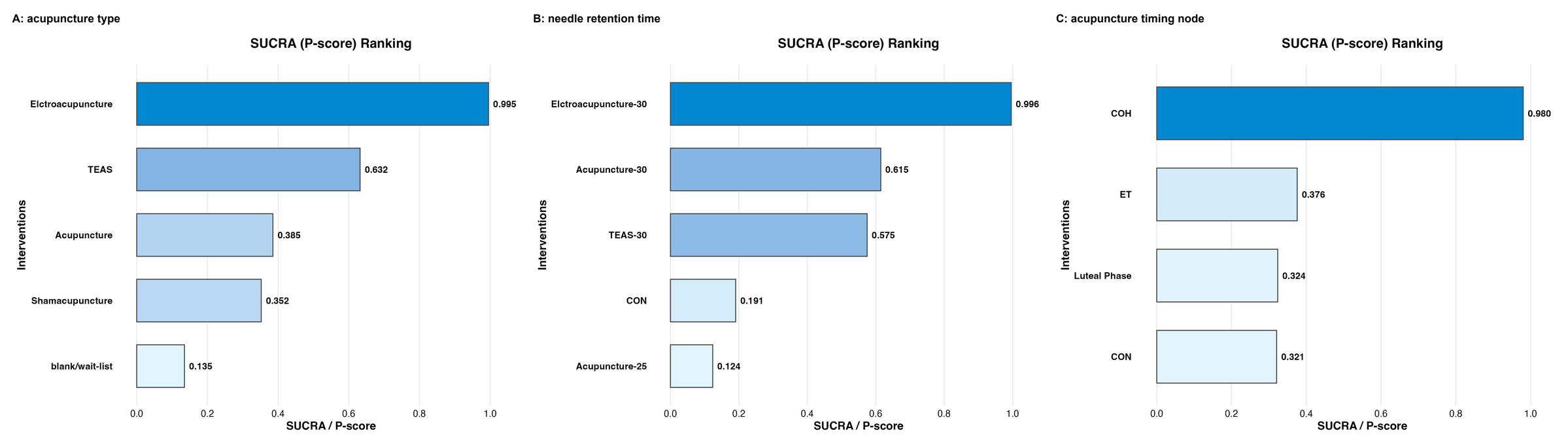


Supplemental Figure 60. SUCRA ranking plots for MR (miscarriage rate). A: acupuncture type; B: needle retention time; C: acupuncture timing node.


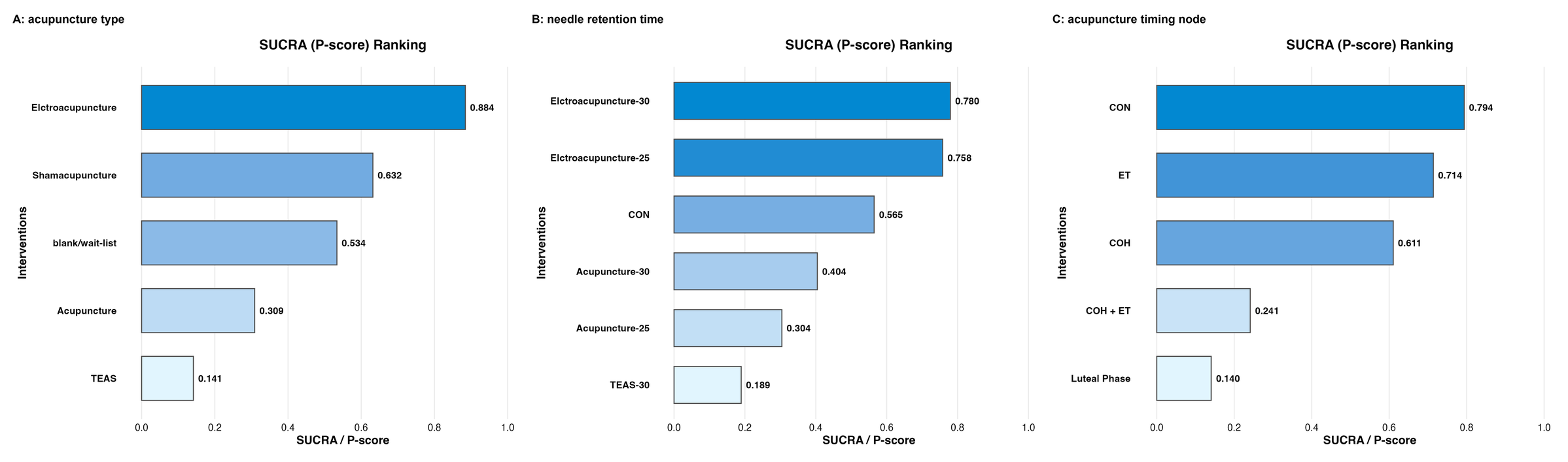


1. **Supplemental File 20. Forest plots for network meta-analysis by acupuncture type, needle retention time, and acupuncture timing node.**

Supplemental Figure 61. Forest plots for CPR (clinical pregnancy rate). A: acupuncture type; B: needle retention time; C: acupuncture timing node.


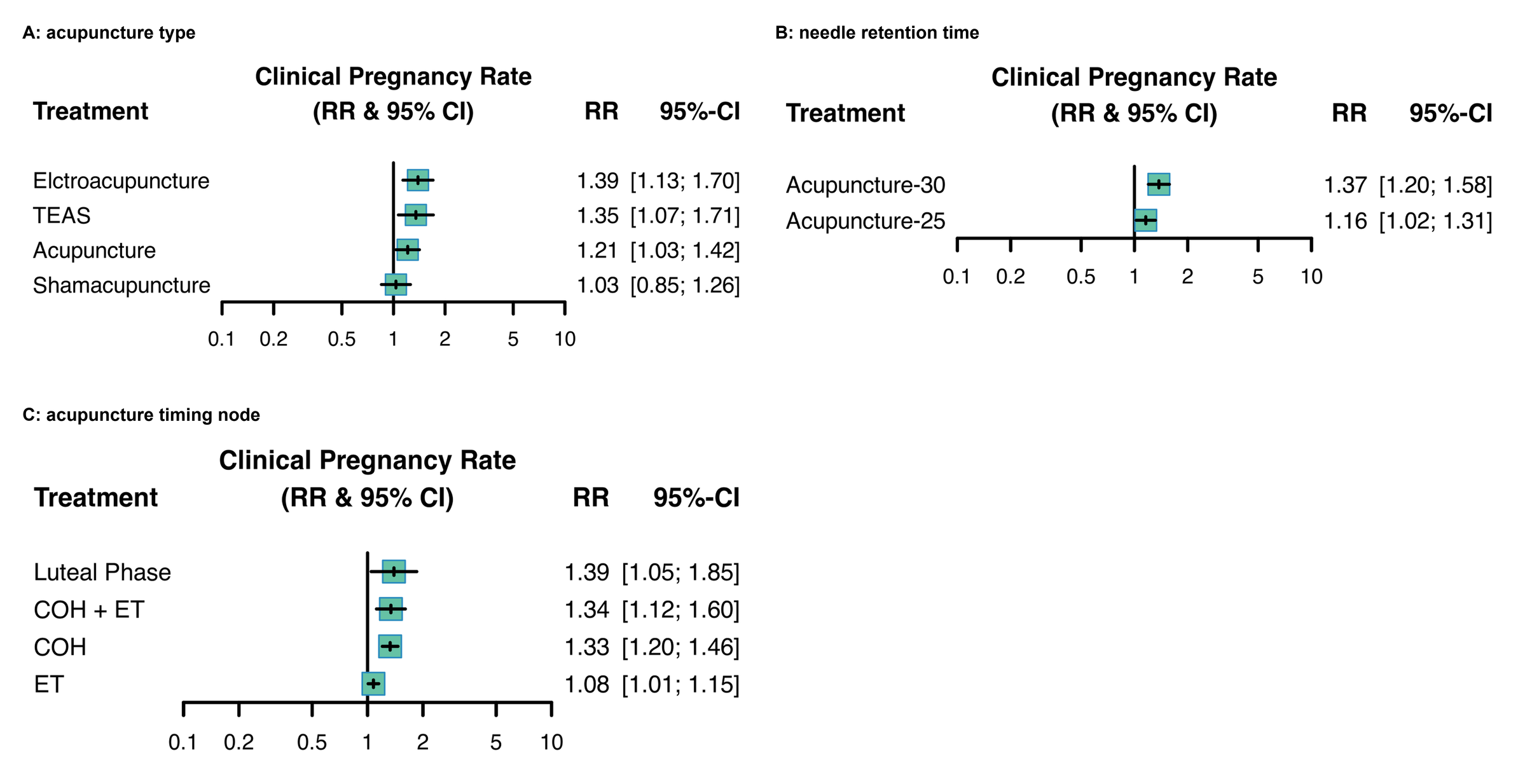


Supplemental Figure 62. Forest plots for LBR (live birth rate). A: acupuncture type; B: needle retention time; C: acupuncture timing node.


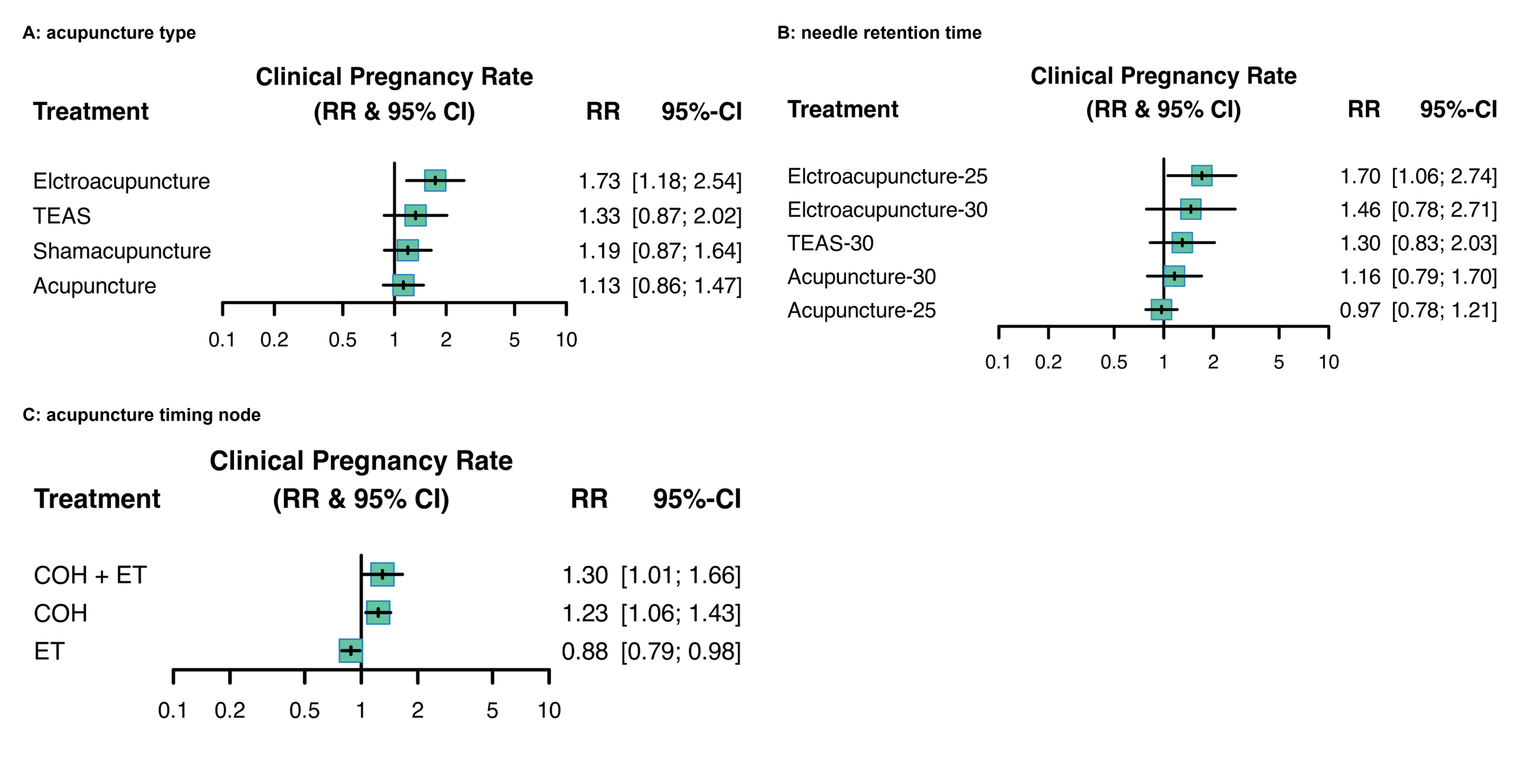


Supplemental Figure 63. Forest plots for OPR (ongoing pregnancy rate). A: acupuncture type; B: needle retention time; C: acupuncture timing node.

Supplemental Figure 64. Forest plots for BPR (biochemical pregnancy rate). A: acupuncture type; B: needle retention time; C: acupuncture timing node.

Supplemental Figure 65. Forest plots for IR (implantation rate). A: acupuncture type; B: needle retention time; C: acupuncture timing node.

Supplemental Figure 66. Forest plots for MR (miscarriage rate). A: acupuncture type; B: needle retention time; C: acupuncture timing node.

1. **Supplemental File 21. League tables for network meta-analysis by acupuncture type and needle retention time.**

Supplemental Figure 67. League tables for CPR (clinical pregnancy rate). A: acupuncture type; B: needle retention time.

Supplemental Figure 68. League tables for LBR (live birth rate). A: acupuncture type; B: needle retention time.

Supplemental Figure 69. League tables for OPR (ongoing pregnancy rate). A: acupuncture type; B: needle retention time.

Supplemental Figure 70. League tables for BPR (biochemical pregnancy rate). A: acupuncture type; B: needle retention time.

Supplemental Figure 71. League tables for IR (implantation rate). A: acupuncture type; B: needle retention time.

Supplemental Figure 72. League tables for MR (miscarriage rate). A: acupuncture type; B: needle retention time.

1. **Supplemental File 22. SUCRA ranking plots for dose-response network meta-analysis by acupuncture type and acupuncture timing node.**

Supplemental Figure 73. Dose-response SUCRA ranking plots for CPR (clinical pregnancy rate). A: acupuncture type; B: acupuncture timing node.

Supplemental Figure 74. Dose-response SUCRA ranking plots for LBR (live birth rate). A: acupuncture type; B: acupuncture timing node.

Supplemental Figure 75. Dose-response SUCRA ranking plots for OPR (ongoing pregnancy rate). A: acupuncture type; B: acupuncture timing node.

Supplemental Figure 76. Dose-response SUCRA ranking plots for BPR (biochemical pregnancy rate). A: acupuncture type; B: acupuncture timing node.

Supplemental Figure 77. Dose-response SUCRA ranking plot for IR (implantation rate) by acupuncture timing node.

Supplemental Figure 78. Dose-response SUCRA ranking plots for MR (miscarriage rate). A: acupuncture type; B: acupuncture timing node.

1. **Supplemental File 23. League tables for dose-response network meta-analysis by acupuncture type and acupuncture timing node.**

Supplemental Figure 79. Dose-response league tables for CPR (clinical pregnancy rate). A: acupuncture type; B: acupuncture timing node.

Supplemental Figure 80. Dose-response league tables for LBR (live birth rate). A: acupuncture type; B: acupuncture timing node.

Supplemental Figure 81. Dose-response league tables for OPR (ongoing pregnancy rate). A: acupuncture type; B: acupuncture timing node.

Supplemental Figure 82. Dose-response league tables for BPR (biochemical pregnancy rate). A: acupuncture type; B: acupuncture timing node.

Supplemental Figure 83. Dose-response league table for IR (implantation rate) by acupuncture timing node.

Supplemental Figure 84. Dose-response league tables for MR (miscarriage rate). A: acupuncture type; B: acupuncture timing node.

1. **Supplemental File 24. Dose-response curves for dose-response network meta-analysis by acupuncture type.**

Supplemental Figure 85. Dose-response curve for CPR (clinical pregnancy rate) by acupuncture type.

Supplemental Figure 86. Dose-response curve for LBR (live birth rate) by acupuncture type.

Supplemental Figure 87. Dose-response curve for OPR (ongoing pregnancy rate) by acupuncture type.

Supplemental Figure 88. Dose-response curve for BPR (biochemical pregnancy rate) by acupuncture type.

Supplemental Figure 89. Dose-response curve for IR (implantation rate) by acupuncture type.

Supplemental Figure 90. Dose-response curve for MR (miscarriage rate) by acupuncture type.

# **Supplemental File 25 Classification of principal findings according to evidential basis.**

| **Evidence classification** | **Finding** | **Evidence basis** | **Recommended interpretation** |
| --- | --- | --- | --- |
| Direct evidence supports benefit | Acupuncture was associated with a higher CPR than control | Direct pairwise comparisons of randomized controlled trials in the primary pairwise meta-analysis | Direct evidence supports a possible improvement in CPR, subject to heterogeneity, risk of bias, small-study effects, and very-low GRADE certainty. |
| Direct evidence supports benefit | Acupuncture was associated with a higher IR than control | Direct pairwise comparisons of randomized controlled trials in the primary pairwise meta-analysis | Direct evidence supports a possible improvement in IR, but this should not be interpreted as definitive efficacy because certainty is limited. |
| Direct evidence inconclusive | No clear benefit was identified for LBR in the primary analysis | Direct pairwise comparisons, with imprecision, heterogeneity, and possible small-study effects | Direct evidence did not confirm an improvement in LBR; this should not be interpreted as equivalence or proof of no effect. |
| Direct evidence inconclusive | No clear differences were identified for BPR, OPR, or MR | Direct pairwise comparisons with imprecise estimates and limited certainty | Direct evidence remains inconclusive and does not establish either benefit or harm. |
| Hypothesis-generating | LBR became statistically significant after excluding studies with concerns in RoB 2 Domain 5 | Sensitivity analysis differing from the primary analysis | This sensitivity finding requires confirmation in prospectively registered, low-risk trials and should not be taken as confirmed evidence of improved live birth. |
| Hypothesis-generating | Certain intervention timings appeared to provide greater benefits | Network estimates combining direct and indirect evidence, including COH, ET, COH + ET, and luteal-phase nodes | Relative timing effects should be interpreted as exploratory because of low certainty, residual transitivity concerns, and timing-dose collinearity. |
| Hypothesis-generating | MA, EA, and TEAS showed different predicted effects across outcomes | Modality-specific subgroup, network, or dose-response models with limited direct head-to-head evidence | Potential modality differences require direct head-to-head trials and should not be described as proven superiority. |
| Hypothesis-generating | Treatment effects varied according to the number of acupuncture sessions | Model-based dose-response predictions from MBNMA | The dose-response pattern is exploratory and should not be used as a treatment-dose recommendation. |
| Hypothesis-generating | Specific session numbers showed the highest model-predicted treatment effects | Model-predicted values, with sparse evidence at some doses | These values should not be interpreted as definitive optimal doses, treatment targets, or confirmed best session numbers. |
| Hypothesis-generating | Treatment effects may differ according to embryo-transfer type, comparator type, treatment timing, modality, treatment sessions, age, BMI, or infertility duration | Subgroup analysis, study-level meta-regression, Meta-CART, or related exploratory moderator analysis | Potential effect modifiers require prospective confirmation because these analyses are vulnerable to residual confounding and multiple comparisons. |

*Note. Findings supported by direct evidence were derived primarily from randomized pairwise comparisons. Findings were considered hypothesis-generating when they were based mainly on indirect network comparisons, subgroup or meta-regression analyses, sensitivity analyses that differed from the primary analysis, or model-based dose-response predictions. The presence of direct evidence does not necessarily indicate a confirmed treatment benefit; some directly evaluated outcomes remained inconclusive because of imprecision, heterogeneity, or limited certainty.*
